# Profiling Sulfur(VI) Fluorides as Reactive Functionalities for Chemical Biology Tools and Expansion of the Ligandable Proteome

**DOI:** 10.1021/acschembio.2c00633

**Published:** 2023-01-17

**Authors:** Katharine E. Gilbert, Aini Vuorinen, Arron Aatkar, Peter Pogány, Jonathan Pettinger, Emma K. Grant, Joanna M. Kirkpatrick, Katrin Rittinger, David House, Glenn A. Burley, Jacob T. Bush

**Affiliations:** † GlaxoSmithKline, Gunnels Wood Road, Stevenage, HertfordshireSG1 2NY, United Kingdom; ‡ University of Strathclyde, 295 Cathedral Street, GlasgowG11XL, United Kingdom; § Crick-GSK Biomedical LinkLabs, GlaxoSmithKline, Gunnels Wood Road, StevenageSG1 2NY, United Kingdom; ∥ The Francis Crick Institute, 1 Midland Road, LondonNW1 1AT, United Kingdom

## Abstract

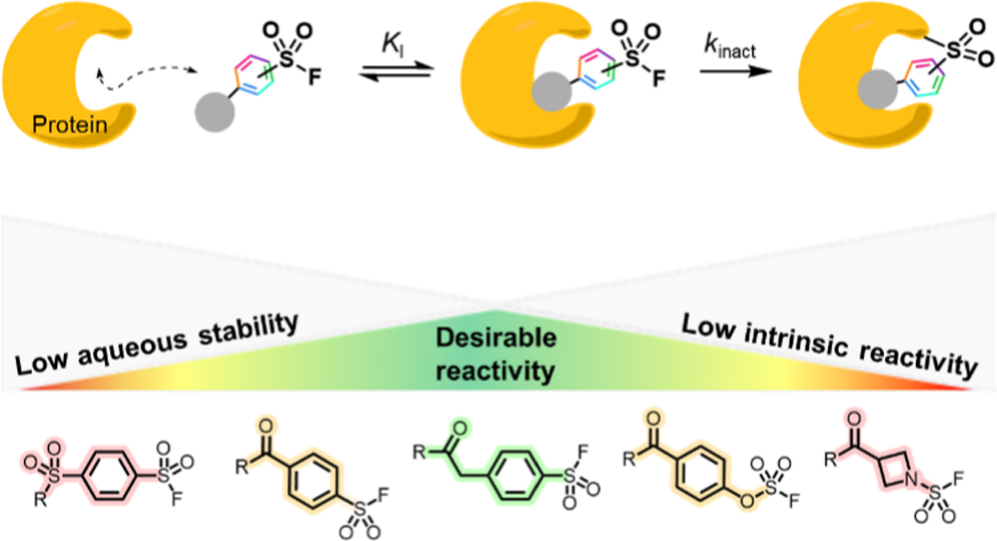

Here, we report a comprehensive profiling of sulfur(VI) fluorides (S^VI^-Fs) as reactive groups for chemical biology applications. S^VI^-Fs are reactive functionalities that modify lysine, tyrosine, histidine, and serine sidechains. A panel of S^VI^-Fs were studied with respect to hydrolytic stability and reactivity with nucleophilic amino acid sidechains. The use of S^VI^-Fs to covalently modify carbonic anhydrase II (CAII) and a range of kinases was then investigated. Finally, the S^VI^-F panel was used in live cell chemoproteomic workflows, identifying novel protein targets based on the type of S^VI^-F used. This work highlights how S^VI^-F reactivity can be used as a tool to expand the liganded proteome.

## Introduction

**Figure 1:**
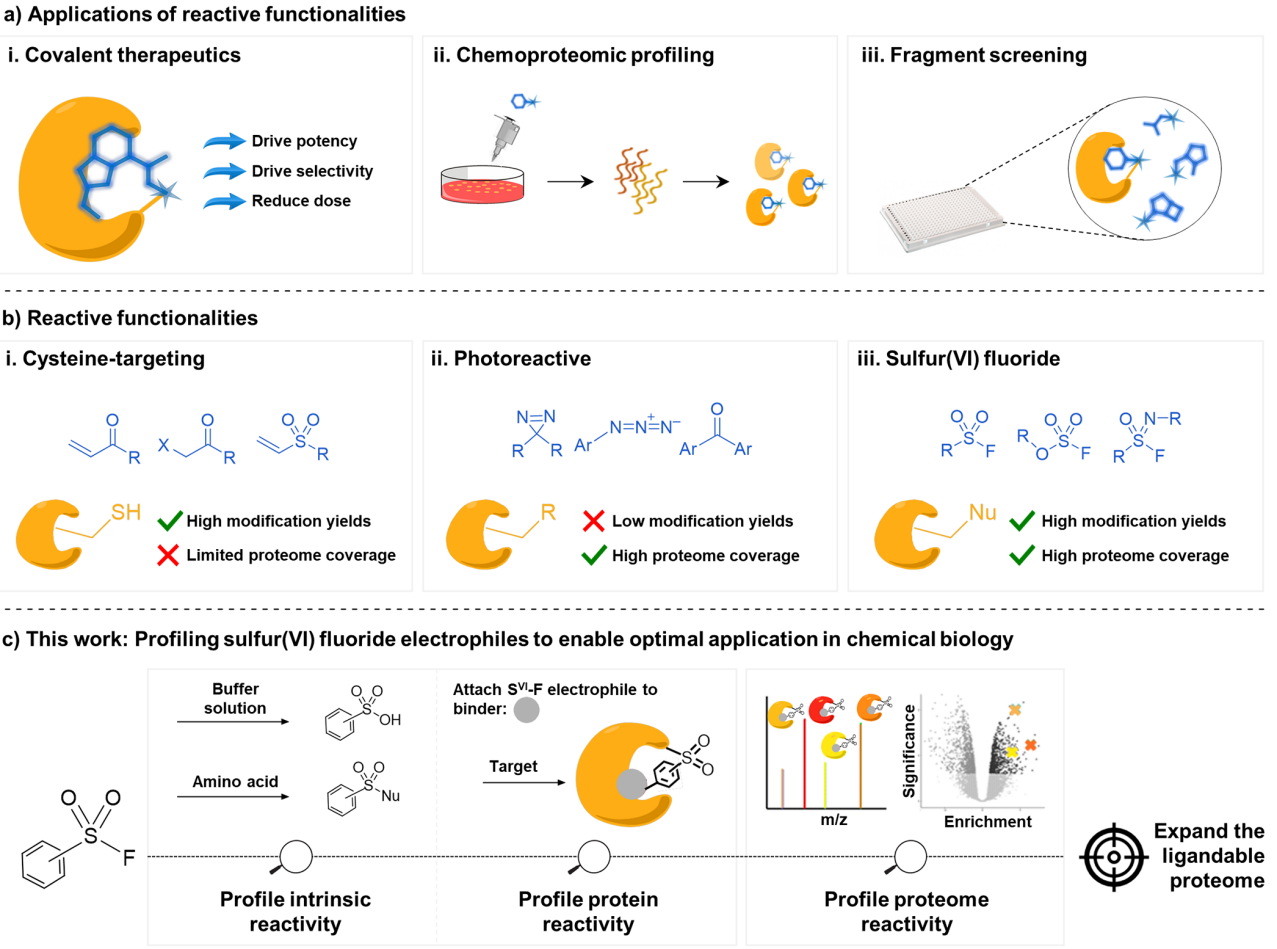
Overview of reactive functionalities in chemical biology. (a) Reactive functionalities hold valuable application in (i) covalent drugs for reduced dosing, (ii) chemoproteomic profiling to assess global target engagement, and (iii) fragment screening to detect transient fragment–protein interactions. (b) S^VI^-Fs offer utility beyond other functionalities typically employed in chemical biology by modifying with high efficiency (cf. Cys-reactive) and broadening proteome coverage (cf. photoreactive). (c) This work: profiling S^VI^-F electrophiles, which will enable their knowledge-guided design for optimal chemical biology application.

Chemical probes offer a molecular toolkit for the study of the proteome and validation of potential therapeutic targets.^1^ Probes comprising reactive functionalities are particularly powerful for the study of protein targets via the covalent modification of selected amino acids.^2,3^ Covalent inhibitors and therapeutics are a proven strategy to enhance potency and selectivity and to reduce dosing frequency (Figure 1a(i)).^4−7^ Reactive tools have also been employed broadly in chemical biology, enabling robust protein capture and providing access to a suite of techniques, including chemoproteomic mapping of ligand–protein interactions across the proteome (Figure 1a(ii)).^8,9^ More recently, reactive fragment-based screening platforms have been developed for streamlined and robust detection of hits, both with purified proteins of interest and in proteome-wide screening, which offers a route to expand the liganded proteome (Figure 1a(iii)).^10−16^

Existing reactive approaches have traditionally utilized cysteine-targeting electrophiles, exploiting the enhanced nucleophilicity of these residues to enable quantitative modification, and often inhibition, of target proteins (Figure 1b(i)).^17−19^ There are, however, a limited number of protein pockets that contain an accessible cysteine. While photoreactive functionalities can provide improved proteome coverage by potentially modifying any residue, the low levels of modification can limit their sensitivity (Figure 1b(ii)).^20−22^ Reactive functionalities that enable robust, high-yielding covalent capture of an expanded set of amino acid residues will diversify the proteome that can be targeted by covalent tools.

Sulfur(VI) fluorides (S^VI^-F) have emerged as a promising electrophilic group for the covalent modification of proteins, reacting with multiple nucleophilic amino acid residues including Tyr, Lys, His, Arg, Ser, and Thr (Figure 1b(iii)).^4,23−37^ Examples of applications include covalent inhibitors, crosslinkers for protein–protein interactions, and chemoproteomic profiling.^9,38−42^ Optimized S^VI^-F inhibitors include the BCL6 inhibitor TMX-2164, TTR stabilizers, and cereblon inhibitor EM12-FS targeting Tyr, Lys and His, respectively.^4,28,43^

S^VI^-F functionalities can achieve a balance of broad protein reactivity alongside high yields of modification. Therefore, S^VI^-F electrophiles present an opportunity to extend the technologies offered by cysteine electrophilic strategies to a considerably broader range of protein targets. Despite their potential both in drug discovery and as chemical biology tools, a consolidated and strategic approach to tuning S^VI^-F reactivity is currently lacking. Multiple factors must be considered for the incorporation of S^VI^-F electrophiles, including susceptibility to competing hydrolysis, reactivity with target proteins, and proteome-wide promiscuity.^44^ A thorough understanding of S^VI^-F reactivity in the context of chemical biology and drug discovery workflows is therefore crucial to the optimal application of these functionalities.

Herein, we present a series of detailed studies on S^VI^-F electrophiles in the context of hydrolytic stability and reactivity, protein modification, and proteome-wide reactivity in live cells (Figure 1c). This profiling workflow revealed that S^VI^-F electrophiles exhibit diverse, yet tunable reactivity across these systems, providing rich insights to guide the strategic design of S^VI^-F reactive tools.

## Results and Discussion

**Figure 2:**
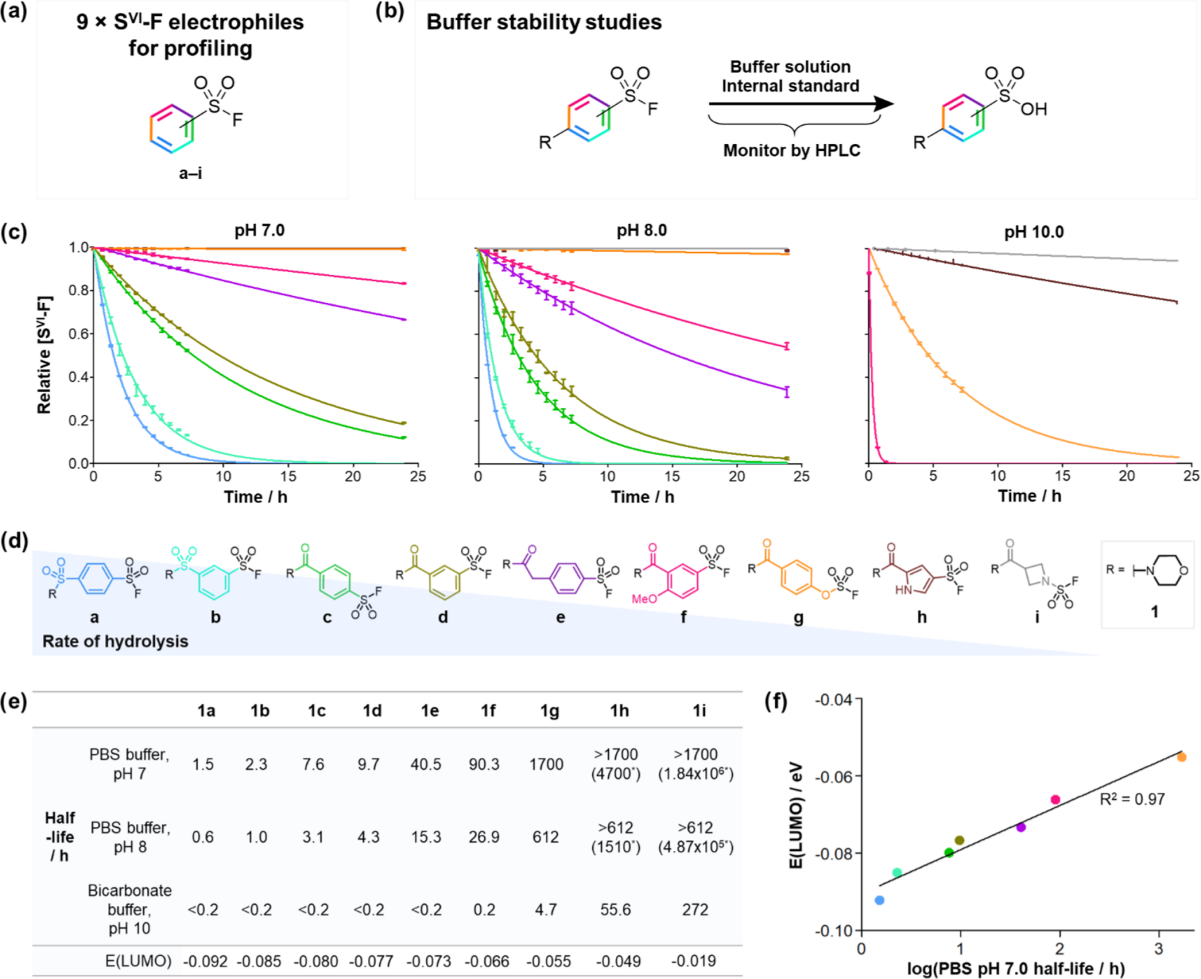
Profiling the hydrolytic stability of S^VI^-F fragments. (a) General structure of S^VI^-F electrophiles (**a**–**i**). (b) Hydrolytic stability measurements using morpholine-substituted S^VI^-Fs **1a**–**i**. (c) Hydrolytic stability profile of S^VI^-Fs **1a**–**i** under various buffer conditions. Line colors correspond to the S^VI^-F electrophiles in (d). (d) Structures of fragments **1a**–**i** ordered by their rates of hydrolysis in buffer solutions. (e) Calculated LUMO energies and experimentally measured half-lives of **1a**–**i** in aqueous buffer solution. *Half-lives predicted using LUMO energy models. (f) Correlation of calculated LUMO energies with measured aqueous half-lives (*R*^2^ = 0.97).

To explore the opportunity to use S^VI^-F electrophiles as tools in chemical biology, we designed a panel of nine S^VI^-F electrophiles (**a**–**i**) (Figure 2a). The S^VI^-F electrophiles contained a carboxylic acid or sulfonyl chloride functionality for conjugation with privileged scaffolds to build reactive tools. Primarily aryl S^VI^-Fs were selected on the basis of their hydrolytic stability compared to aliphatic analogues, which typically undergo facile elimination via a sulfene intermediate.^45^ Heteroatom-linked S^VI^-F functionalities were also included, e.g., fluorosulfate (**g**) and sulfamoyl fluoride (**i**). The set included a range of electron-withdrawing and electron-donating substituents to assess the influence of electronics on reactivity and consisted of multiple matched pairs to enable the investigation of point electronic changes upon intrinsic reactivity.

### Stability, Reactivity, and Biochemical Protein Modification

Hydrolytic stability and target reactivity are key parameters in determining the suitability of an electrophile for application in biochemical and cellular studies. We first investigated both the hydrolytic stability of S^VI^-F electrophiles and the reactivity toward amino acids using a morpholine ring as a representative building block of a druglike molecule. S^VI^-F electrophiles (**a**–**i**) were coupled to morpholine by nucleophilic substitution chemistry or HATU-mediated amide couplings to furnish the S^VI^-F fragment panel **1a**–**i**.

The hydrolytic stability of S^VI^-F fragments **1a**–**i** was investigated by incubation with PBS and HEPES buffers at pH 7 and 8 to span the physiological pH range. An additional experiment was undertaken in carbonate-bicarbonate buffer at pH 10 to further differentiate S^VI^-F reactivity. Rates and half-lives of hydrolysis were determined by monitoring the depletion of S^VI^-F fragments using high-performance liquid chromatography (HPLC) (Figure 2b).

S^VI^-F fragments **1a**–**i** exhibited a considerable range of aqueous stabilities, with measured half-lives from 35 min to >600 h (Figures 2c–e and S1–S5 and Table S1). Hydrolysis rates were accelerated under basic conditions, with half-lives at pH 8 approximately 2-fold lower than at pH 7 (PBS) and dramatically reduced at pH 10 (carbonate-bicarbonate buffer). Hydrolytic stability was approximately 2-fold greater in HEPES vs PBS for all fragments at pH 7 and 8, indicating an influence of buffer identity on S^VI^-F stability. The aqueous stability of the fragments was found to be independent of NaCl concentration.

The order of intrinsic reactivity of **1a**–**i** correlated well with the electronic factors that influence the electrophilicity of the sulfur center. *Para*-amide and -sulfonamide S^VI^-F electrophiles hydrolyzed faster than the *meta* analogues (**1a** vs **1b**, **1c** vs **1d**). Substituents that increased the electron density on the phenyl ring imparted marked stabilization, as observed with the addition of a methylene spacer (**1e** vs **1c**) and *para*-methoxy moiety (**1f** vs **1d**). The fluorosulfate **1g**, pyrrole **1h**, and N-linked S^VI^-F electrophile **1i** displayed the greatest stability, undergoing negligible hydrolysis over 24 h at pH 8. The experiment performed at pH 10 (bicarbonate buffer) revealed reactivities in the order fluorosulfate **1g** > pyrrole **1h** > sulfamoyl fluoride **1i**.

It was anticipated that methods for the prediction of hydrolytic stability would be valuable to guide the design of novel S^VI^-F reactive tools. Previous reports have observed a correlation between S^VI^-F half-life and Hammett values.^44^ Here, we sought a more generalizable approach by employing energy calculations to determine whether a correlation exists between hydrolytic reactivity and lowest unoccupied molecular orbital (LUMO) energy. Three quantum mechanical approaches were employed. First, a semiempirical method was used (AM1), which gave poor correlation with the half-lives of S^VI^-Fs **1a**–**g** (*R*^2^ = 0.31).^46^ Subsequently, two higher-level DFT methods were employed to improve accuracy: B3LYP-D3 with 6-31+G** and B3LYP-D3 with aug-cc-PVTZ, which provided the best accuracy.^47,48^ LUMO energies directly correlated with the aqueous half-lives of S^VI^-F electrophiles **1a**–**g** (*R*^2^ = 0.97) (Figure 2f). This relationship provides an accessible route to the prospective design of S^VI^-F modalities that occupy the desired reactivity space, enabling the prioritization of S^VI^-Fs for direct synthetic incorporation into chemical tools.

**Figure 3:**
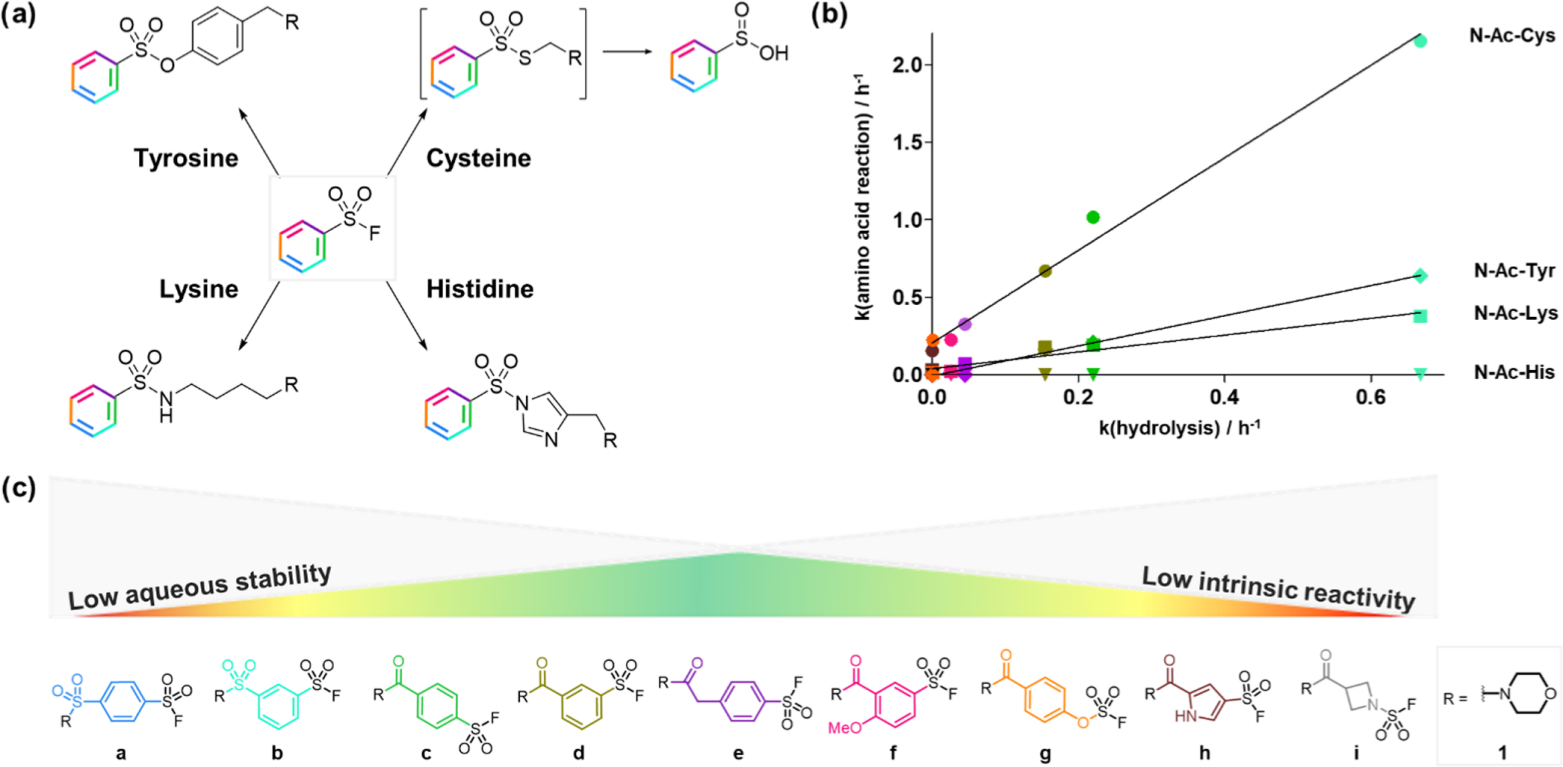
Profiling the amino acid reactivity of S^VI^-F fragments. (a) Amino acid adducts formed with S^VI^-F fragments and nucleophilic amino acids. (b) Correlation between rate constants for hydrolysis and for reaction with *N*-acyl-protected nucleophilic amino acids in PBS at pH 8.0. The rate of reaction of fragment **1a** with amino acids was too high for accurate rate constant measurement. (c) S^VI^-F electrophiles will ideally possess a balance between aqueous stability and intrinsic reactivity: (**d**–**f**).

Hydrolytic stability has provided key insight into the behavior of S^VI^-F functionalities, though a balance must be achieved with amino acid reactivity to ensure that protein modification occurs at an appropriate rate. S^VI^-F electrophiles have been reported to react with Lys, Tyr, and His residues to form stable covalent adducts. While the nucleophilicity and p*K*_a_ of amino acid residues are known to be perturbed in protein environments, we initially studied the reactivity of the S^VI^-F electrophiles with individual amino acids to establish benchmarking data.^44^ We initially studied the reactivity of **1a**–**i** with monomeric amino acids: *N*-acetyltyrosine, *N*_α_-acetyllysine, *N*-acetylhistidine, and *N*-acetylcysteine (Figures 3a and S6). Reaction rates were monitored by HPLC and fit using pseudo-first-order kinetics, as established by the 10-fold excess of amino acid.^49^

The reactivity of fragments **1a**–**i** with the amino acids was found to closely correlate with the rate of hydrolysis (Figure 3b). This correlation indicated that there was no opportunity within this set of S^VI^-F electrophiles, to tailor an S^VI^-F for preferential reactivity with an amino acid vs hydrolysis (Figure 3c). The amino acid reactivity increased in the order *N*-Ac-His <*N*_α_-Ac-Lys <*N*-Ac-Tyr <*N*-Ac-Cys, which is consistent with the nucleophilicity of the amino acids at physiological pH.^50^ It is important to note the fastest reaction with cysteine; however, this affords an unstable thiosulfonate ester adduct that collapses to the corresponding sulfinic acid.^44,51^ Reactions with *N*-Ac-Tyr and *N*_α_-Ac-Lys afforded the expected sulfonate ester and sulfonamide respectively, with reaction at tyrosine occurring at approximately twice the rate of lysine. *N*-Ac-His did not form any adduct and only hydrolysis was observed, which occurred at the same rate as previously measured in our hydrolysis studies.

**Figure 4:**
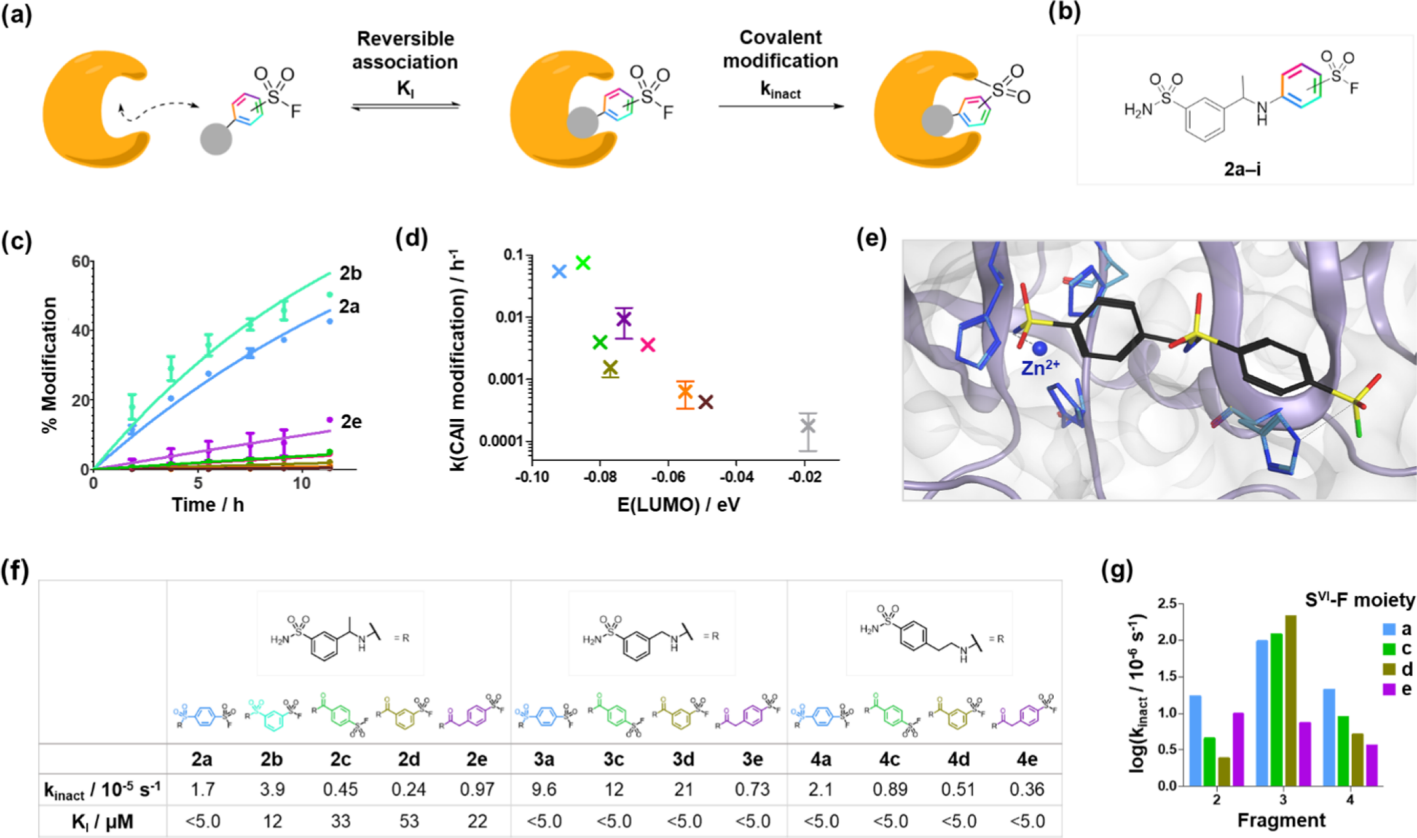
Profiling the protein reactivity of S^VI^-F fragment binders. (a) Covalent modification of a protein target by an S^VI^-F electrophilic inhibitor. (b) S^VI^-F electrophiles (**a**–**i**) were substituted onto a CAII hit fragment to afford analogues **2a**–**i**. (c) Modification of CAII (1 μM) by fragments **2a**–**i** (10 μM) over time. (d) Correlation between the rate of CAII modification and intrinsic reactivity (LUMO energy). (e) X-ray crystal structure of CAII (PDB: 2VVB) virtually docked with fragment **4a**. (f) Measured *k*_inact_ and *K*_I_ parameters for the irreversible modification of CAII by S^VI^-F fragments at 4 °C. (g) Measured *k*_inact_ values showed relatively poor correlation with S^VI^-F intrinsic reactivity.

We subsequently profiled the performance of S^VI^-F electrophiles in the context of covalent modification of a target protein. Covalent modification of protein targets by electrophilic inhibitors occurs in two steps: (i) The inhibitor and target engage in a reversible binding interaction (*K*_I_) and (ii) the electrophile reacts irreversibly with the target to form a covalent adduct (*k*_inact_) (Figure 4a). Carbonic anhydrase II (CAII) was selected as a model system to investigate the rate of protein crosslinking by the panel of S^VI^-F electrophiles. A recent screen within our group had identified a reactive fragment hit for CAII based on an aryl sulfonamide. The nine S^VI^-F electrophiles were coupled to the CAII hit fragment to afford analogues **2a**–**i** (Figure 4b). The rate of CAII modification by fragments **2a**–**i** was monitored by intact-protein liquid chromatography–mass spectrometry (LC–MS) (Figure 4c). Rates followed the approximate order of electrophile intrinsic reactivity (Figure 4d), with the two highly reactive sulfonamide-linked fragments **2a** and **2b** displaying the highest rate of modification, while the least reactive fragments **2g**, **2h**, and **2i** gave the lowest rates. Interestingly, the remaining fragments **2c**–**f** displayed modification rates that diverged from intrinsic reactivity.

S^VI^-F electrophiles (**a**), (**c**), (**d**), and (**e**) were selected for further kinetics studies to deconvolute the contributions of the reversible binding (*K*_I_) and the covalent reaction (*k*_inact_). Two additional CAII binding sulfonamides **3** and **4** were also coupled to these electrophiles to explore how the variation of fragment structure and orientation can affect the kinetic parameters. Fragment **3e** was previously found to covalently modify a histidine proximal to the Zn(II) active site.^52^ The rates of covalent modification were measured over a range of concentrations to enable the determination of the kinetic parameters, *K*_I_ and *k*_inact_.

The reversible affinity of the fragments was typically beyond the limit of the assay (*K*_I_ <5 μM), consistent with previous reports of aryl primary sulfonamides showing sub-micromolar affinity for CAII (Figure 4e,f).^53,54^ The rates of covalent modification (*k*_inact_) showed little correlation with the intrinsic reactivity of the S^VI^-F electrophile (Figure 4g). The results showed some correlation for a given fragment series, e.g., **4a**,**c**,**d**,**e** and **2a**,**c**,**d**; however, there were several outliers where *k*_inact_ did not meet the expected value based on the measured intrinsic reactivity, e.g., **2e** and **3a**,**c**,**d**. Interestingly, significant changes in *k*_inact_ were observed on variation of the sulfonamide fragment. S^VI^-F electrophiles (**a**), (**c**), and (**d**) showed much greater reactivity when appended to sulfonamide **3** vs sulfonamides **2** and **4**, suggesting that **3** positioned the S^VI^-F functionality more optimally for crosslinking.

The intrinsic and protein reactivity profiling highlights the opportunity to tailor S^VI^-F electrophiles to span a profound range of reactivities. S^VI^-F functionalities that occupy an ideal reactivity space for application to chemical probes will demonstrate sufficient reactivity, while maintaining good aqueous stability at physiological pH (>4 h aqueous half-life). Among the set of S^VI^-F electrophiles, **1d**–**f** fit these criteria with half-lives spanning 10–90 h and 4–27 h at pH 7 and 8, respectively (Figure 3b,c).

This corresponds to LUMO energies between −0.08 and −0.06 eV, providing guidance for the design of S^VI^-F functionalities prior to incorporation in chemical tools.

### Proteome Reactivity

**Figure 5:**
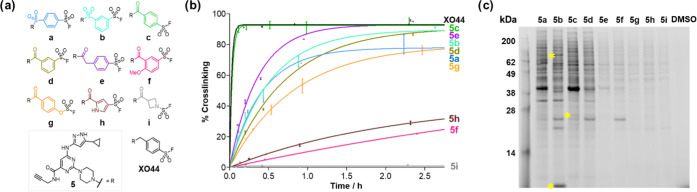
Profiling the protein and lysate reactivity of S^VI^-F kinase probes. (a) Structures of S^VI^-F kinase probe analogues **5a**–**i** and **XO44**. (b) Modification of recombinant CDK2 protein by S^VI^-F kinase probes over time. (c) Gel electrophoresis showing the proteome-wide modification by probes **5a**–**i** in cell lysate. Yellow asterisks indicate examples of bands corresponding to potentially selective protein modifications. The lanes have been reordered according to intrinsic reactivity. The original gel is shown in Figure S7.

Next, we investigated the ability of these electrophiles to capture proteins in cells using chemoproteomics. Recent work from the Taunton laboratory reported **XO44** as an S^VI^-F probe for kinase proteins, which was shown to capture kinases from cells by chemoproteomics.^34^**XO44** was then used to quantify kinase target enrichment by the approved drug, dasatinib. The S^VI^-F electrophile was positioned to react with the conserved catalytic lysine residue in the kinase ATP-binding pocket. We anticipated that this would provide a system to explore the reactivity of our S^VI^-F electrophiles against the kinome and wider proteome in live cells. A panel of **XO44** analogues were synthesized incorporating each of the nine S^VI^-F electrophiles: **5a**–**i** (Figure 5a).

An initial assessment of kinase reactivity of probes **5a**–**i** was performed with recombinant CDK2 protein by intact-protein LC–MS (Figure 5b). Probes **5c** (probe **1** in ref (34)) and **XO44** achieved rapid, quantitative modification, suggesting that *para*-substituted S^VI^-Fs were optimal probe structures for CDK2. The majority of the remaining probes exhibited similar *k*_obs_ (0.3–0.8 × 10^–3^ s^–1^) despite the variance in intrinsic reactivity, highlighting influences of sterics and orientation on the rates of modification. The conversion by the most reactive probes **5a** and **5b** plateaued at ∼75 and 90% respectively, potentially reflecting the impact of competing hydrolysis with highly reactive S^VI^-F functionalities. The impact of intrinsic reactivity was only apparent for three low reactivity electrophiles: (**f**) and (**h**) that underwent the slowest modification, and **5i** that did not yield any modification of CDK2.

Subsequently, the proteome-wide reactivity of the S^VI^-F probes was investigated by gel electrophoresis. Probes **5a**–**i** were incubated with lysate for 2.5 h, followed by click conjugation with Cyanin5.5 azide, gel electrophoresis, and fluorescence visualization (Figures 5c and S7). Probes **5a**–**d** exhibited high levels of proteome modification while weak labeling was observed for probes **5e**–**i**, in agreement with the intrinsic reactivity of the S^VI^-F electrophiles. Several unique bands were observed for various probes, which are indicative of selective target–probe interactions.

**Figure 6:**
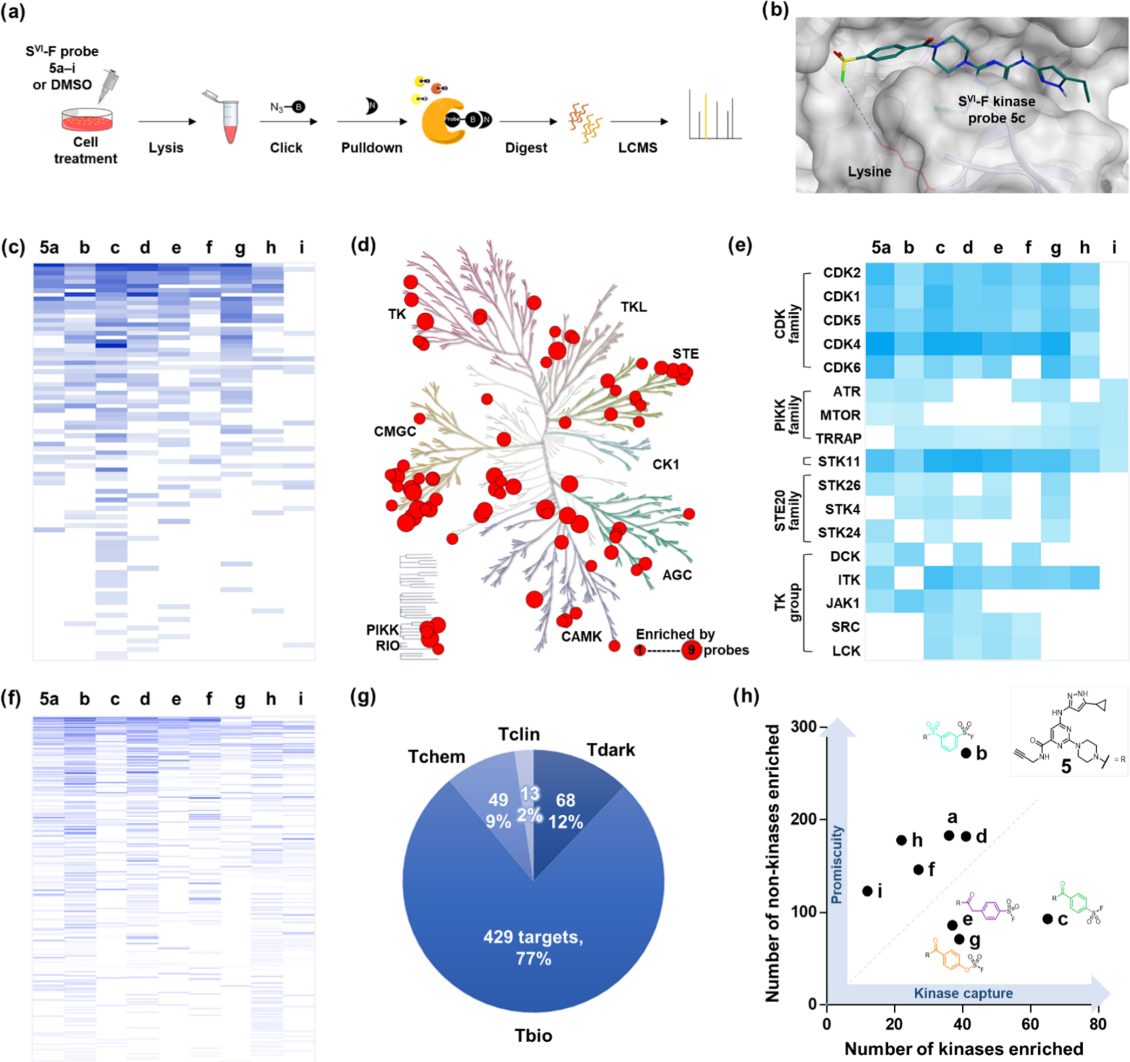
Chemoproteomic profiling of S^VI^-F kinase probes. (a) Chemoproteomic workflow undertaken to identify proteins modified by probes **5a**–**i** in live cells. (b) X-ray crystal structure of CDK2 (PDB: 6INL) virtually docked with probe **5c**. (c) Heatmap of the kinases enriched by probes **5a**–**i** (*q* < 0.05, log_2_-fold > 0.58). The color scale indicates magnitude of log_2_-fold change: 0.58–5.0. (d) Phylogenetic kinome tree, showing the number of probes that enriched various kinases. (e) Heatmap describing the enrichment of particular protein targets by probes **5a**–**i**. (f) Heatmap of the nonkinases enriched by probes **5a**–**i** (*q* < 0.05, log_2_-fold > 0.58). The color scale indicates magnitude of log_2_-fold change: 0.58–5.0. (g) Summary of the target development levels of non-kinase proteins. (h) Numbers of kinases and nonkinases enriched by each probe.

Proteome-wide target engagement was analyzed in further detail by live cell chemoproteomics. Jurkat T cells were incubated for 1 h with alkyne-tagged probes **5a**–**i** and **XO44** or DMSO vehicle in biological triplicate, before cell lysis and CuAAC reaction with biotin-PEG3-azide. Labeled proteins were enriched, digested, and analyzed by LC–MS/MS using label-free quantification and data-independent acquisition (Figure 6a). Enriched kinases were identified by comparison to the DMSO control (two-sample *t*-test, *q* < 0.05, log_2_-fold change >0.58). In total, probes **5a**–**i** enriched 94 kinases, among which 51 were engaged by three or more probes and 33 were enriched by just one probe (Figure 6b,c). An additional 29 kinases were enriched by **XO44**, highlighting kinases where capture perhaps benefits from greater linker flexibility. Conversely, 20 kinases were detected by probes **5a**–**i** that were not enriched by **XO44**. The number of enriched kinases for each probe displayed poor correlation with intrinsic reactivity. Probes **5c** and **5g** enriched high numbers of kinases (65 and 39) relative to electrophiles with similar intrinsic reactivity (**5b** and **d**, **5f** and **h**, respectively). The structural similarity of these two probes (*para*-amide sulfonyl fluoride and fluorosulfate, respectively) points to reversible recognition and/or the trajectory of the reacting species to the conserved lysine as being key determinants of covalent capture. The impact of S^VI^-F intrinsic reactivity was only apparent for the low reactivity probes **5f**, **5h**, and **5i**, which enriched the fewest kinases (Figure 6c).

The coverage of the kinase phylogenetic tree by our probe set revealed high representation of certain subgroups of kinases, such as the CMGC group (17/34 kinases), and TK group, including ITK, JAK1, SRC, and LCK that are of therapeutic relevance.^55^ Other classes were poorly represented, such as the CAMK group (6/31) and CK1 groups (0/10), highlighting opportunities for further probe development (Figure 6d).^56^

Analysis of the number of probes that captured each kinase provided insights into the specificity and SAR of kinase capture. Many proteins were enriched by a number of probes, e.g., CDK1,2,4,5 were enriched by all probes except **5i**, which was consistent with our recombinant CDK2 studies (Figures 5b and 6e). Conversely, the three detected PIKK family kinases were all enriched by **5i** (Figure 6e), indicating that sulfamoyl fluorides can perform covalent capture in specific environments. Notably, the tumor suppressor serine/threonine kinase, STK11, was the only kinase enriched by all nine probes.^57^ S^VI^-F orientation appears to influence protein capture, as observed with the enrichment of STK24 by only the three *para*-substituted S^VI^-F electrophiles and DCK enrichment by *meta*-substituted probes **5b**, **5d**, and **5f**. Certain enrichment profiles suggested S^VI^-F reactivity-driven target modification, where engagement is observed by only the highly reactive probes **5a**–**d**, e.g., JAK1. Additionally, the modification of SRC and LCK by probes **5c**–**f** may indicate reactivity-dependent modification in combination with poor tolerance for the sulfonamide linkage present in probes **5a** and **5b**.

Enrichment analyzes were subsequently performed across the remaining proteome to determine the promiscuity of probes (Figure 6f). A total of 559 nonkinases were found to be enriched by probes **5a**–**i** (*q* < 0.05, log_2_-fold change > 0.58). We assessed the opportunity to expand the liganded proteome using S^VI^-F electrophiles by examining the target development level (TDL) of captured proteins that classifies the extent of knowledge around tools for each protein (Figure 6g).^58^ Of the enriched proteins, 497 (89%) were categorized as Tdark and Tbio, for which no quality binders are known. Probes **5a**–**i** may help address the knowledge deficit concerning Tdark and Tbio targets. It should be noted however that further work will be required to validate these interactions to confirm that modification is specific and selective. We also compared our enrichment profile with those of six published cysteine-targeting proteomics studies.^3,17,19,59−61^ It was found that 217 (39%) non-kinase proteins captured here were not robustly identified across the six Cys-targeting studies (0/6 or 1/6 studies), demonstrating the potential for S^VI^-Fs to contribute to expanding the liganded proteome. Inspection of our enriched proteins identified 12 hydrolases and 11 serine proteases that possess nucleophilic binding site residues, which may be expected to react with S^VI^-F electrophiles. Further, many protein targets that are traditionally challenging to target were captured, including 21 mRNA splicing factors, 10 ligases, 17 ribosomal proteins, and 13 transcription factors. Three nonkinases were enriched by all nine probes, including RBMX and ERCC4 that both have therapeutic relevance, while 192 nonkinases (34%) were modified by three or more probes, highlighting a potential opportunity to develop selective S^VI^-F chemical probes for these proteins.^62,63^ The least reactive probe **5i** modified few proteins, but interestingly, it was the sole probe to modify a set of 20 nonkinases, suggesting the presence of privileged interactions.

Proteome promiscuity showed some correlation with S^VI^-F intrinsic reactivity, with probes **5a**–**e** generally enriching more proteins than probes **5f**–**i** (Figure 6f). The probes appeared to display an inverse relationship between kinome and proteome enrichment whereby probes that labeled large numbers of kinases, e.g., (**c**) and (**g**), were not observed to modify other proteins in the proteome (Figure 6h). This highlights a competition between kinome and off-target labeling among the panel and illustrates that subtle changes in S^VI^-F electrophile structure can alter the covalent capture profile.

## Conclusions

Sulfur(VI) fluorides enable covalent capture of multiple amino acid residues, thus offering profound utility in the development of tools for chemical biology and expansion of the liganded proteome. To fully realize the potential of these “beyond cysteine” covalent capture approaches, an in-depth understanding of the parameters that determine protein capture is required. The assessments of S^VI^-F stability, reactivity, protein modification kinetics, and chemoproteomic performance have provided insights into how these functionalities can be deployed for the prospective design and application of S^VI^-F reactive tools.

The panel of S^VI^-Fs studied here displayed a large range of reactivities and stabilities, highlighting that the electrophiles are highly tunable for reaction with a target of interest while minimizing aqueous hydrolysis and off-target reactivity. Three S^VI^-Fs (**d**–**f**) exhibited desirable properties for application in biological systems and provide starting points for the generation of reactive tools. It is anticipated that as ligands are developed with increasing reversible affinity, it will be possible to substitute less reactive S^VI^-Fs such as (**h**) and (**i**), to enhance selectivity. Furthermore, this work has enabled the prediction of S^VI^-F reactivity via LUMO energy calculations, providing a valuable method to tune S^VI^-F tools and offering a foundation for the design of novel S^VI^-F ligands with desirable reactivity.

Studies on protein modification kinetics revealed some correlation between *k*_inact_ and S^VI^-F intrinsic reactivity, particularly when comparing across a large range of LUMO energies. Where the probes exhibited intermediate intrinsic reactivity (e.g., **d**–**f**), the protein kinetics were more nuanced, highlighting the influence of structural and orientational effects on modification rates, such as the trajectory of the electrophile toward the residue, and local protein–electrophile interactions that perturb reactivity.^11,64,65^ For inhibitor optimization, it will likely be necessary to incorporate multiple different S^VI^-Fs electrophiles to identify those that form protein interactions that optimize *K*_I_ and *k*_inact_.

Live cell proteomic profiling of the S^VI^-F panel linked to a pan-kinase inhibitor indicated that all electrophiles gave a high selectivity toward capture of kinases and good coverage of the kinome. This highlights a tolerance of a broad range of S^VI^-F electrophiles in chemoproteomic profiling, perhaps facilitated by the use of a potent kinase scaffold. Further studies to explore how this observation varies with less potent compounds will be informative, particularly with respect to the application of S^VI^-F fragment-based approaches in live cells.^66^

Evaluation of the nonkinome proteins that were enriched identified hundreds of additional targets, including many that have not been liganded to date (categorized as Tdark and Tbio). These probe–protein interactions may provide useful starting points for the development of S^VI^-F reactive tools. The diversity of protein classes captured highlights the capacity for S^VI^-Fs to modify a broad scope of the proteome, much of which is not traditionally considered to be tractable.

Together, these results provide confidence that S^VI^-Fs are highly complementary to cysteine-reactive approaches, enabling translation of the opportunities offered by traditional reactive tools to a broader proportion of the proteome.

## Methods

Full experimental details including synthesis and data processing are provided in the Supporting Information.

### Hydrolysis Studies

To each S^VI^-F compound (60 μL, 10 mM in DMSO) was added DMSO (80 μL) and either 1,4-dicyanobenzene solution or methyl *p*-tolyl sulfone solution (as an internal standard, 60 μL, 10 mM in DMSO), then finally, buffer solution (0.1 M, 800 μL). The vial was mixed and then analyzed by HPLC (2 μL injection volume) at intervals of approximately 40 min.

### Amino Acid Reactivity Studies

To each S^VI^-F compound (20 μL, 10 mM in DMSO) was added amino acid stock solution (980 μL, composition as described in Tables S7 and S8), then the solution was mixed with a pipette. The vial was analyzed by HPLC (2 μL injection volume) at intervals of approximately 40 min.

### LUMO Energy Calculations

Three-dimensional (3D) structures were created using LigPrep and conformer generation was performed with Schrödinger Macromodel. Geometry optimization of the resulting conformations was carried out using Gaussian16 using an aug-cc-PVTZ basis set and a polarizable continuum model (PCM) solvation model with water. Geometry optimization was used to select the most stable conformer for each structure (at each level). The LUMO orbital energy was calculated for the lowest-energy conformer of each structure.

### Recombinant Protein Reactivity Studies

Experiments were performed in duplicate. S^VI^-F compound (5–100 μM) or DMSO (FAC 1%) was combined with recombinant protein (1 μM) in buffer (pH 7.5, 25 mM HEPES, 150 mM NaCl) with mixing. The plate was centrifuged (1000 rpm, 1 min) and then subjected to intact-protein LC–MS analysis across a time course.

### Gel Electrophoresis

Cleared lysate (25 μL, 1 mg mL^–1^) in lysis buffer was mixed with each S^VI^-F probe (0.5 μL, 0.5 mM, FAC 10 μM) or DMSO (FAC 1%). The aliquots were incubated on ice for 2.5 h. To 15 μL of each solution was added Cy5.5-N_3_ (0.125 mM, 2 μL), followed by THPTA click mix (1 μL). To 450 μL of Invitrogen Gel Loading Buffer II was added DTT (50 μL, 1 mM). The samples were left on ice for 1 h before DTT-loading buffer solution (5 μL) was added. A NuPAGE 12% Bis-Tris gel was prepared in 1× NuPAGE MES SDS running buffer and SeeBlue Plus2 Pre-stained Protein Standard (10 μL) in the first column. Each probe sample (8 μL) was added in separate lanes of the gel. The gel was run for 45 min at constant voltage (200 V, 120 mA, 25.0 W) and analyzed on an LI-COR gel reader using Image Studio Lite.

### Chemoproteomic Studies

Jurkat cells (2 × 10^6^ cells mL^–1^ in serum-free RPMI media) were treated in triplicate for 1 h with each S^VI^-F probe (FAC 2 μM) or DMSO at 37 °C. Treated cells were pelleted and washed with PBS and then sonicated (5 s × 1 s) in lysis buffer. Each lysate (376 μL, concentrations adjusted to 2.3 μg μL^–1^) was treated with click mixture (24 μL) for 1 h and then EDTA was added (8 μL, 500 mM, FAC 10 mM). Proteins were precipitated using ice-cold acetone, and the resulting pellets were washed (2× in ice-cold 80% acetone). The air-dried pellets were dissolved in SDS (400 μL, 0.2%) in HEPES (50 mM, pH 8.0) by vortexing and sonicating. Samples were incubated with acetylated NeutrAvidin agarose resin for 2 h. The plate was centrifuged, then the beads were washed with lysis buffer (3× each), urea (4 M in 50 mM HEPES pH 8.0), and HEPES (50 mM pH 8.0). The proteins were digested on-bead overnight at 37 °C with LysC (60 μL, 0.004 μg μL^–1^ in 50 mM HEPES pH 8.0). The supernatants were collected and incubated with trypsin (40 μL, 0.006 μg μL^–1^ in 50 mM HEPES pH 8.0) for 4 h at 37 °C, then formic acid (1 μL) was added. TFA (0.1% in water, 100 μL) was added to each sample before loading onto a preconditioned C18 96-well plate. The plate was centrifuged, then the samples were washed twice with TFA (0.1% in water, 200 μL), and centrifuged twice. The peptides were eluted twice with TFA (0.1% in 50% MeCN, 150 μL) into a collection plate and centrifuged. The plate was frozen, and the samples were dried in a Labconco CentriVap Benchtop Vacuum Concentrator at 35 °C. Peptides were redissolved in formic acid (0.1% in water), then 40% of each digested sample and iRT standards (Biognosys AG) were loaded onto Evotips, followed by loading onto the Evosep One system. Data were acquired in data-independent acquisition (DIA) mode. The data were searched against a generated spectral library, then run-wise imputation (*Q*-value percentile = 30%) was applied to the dataset. A two-sample *t*-test was carried out in Spectronaut software, then filters were applied to the data (*q* ≤ 0.05, avg log_2_-fold change ≥ 0.58, no. unique total peptides ≥ 2).

## Supporting Information Available

Materials and methods; compound synthesis and characterization data (Figures S1–S7 and Table S1); and biological studies (PDF)
