# Supplementary material for: Profiling Sulfur(VI) Fluorides as Reactive Functionalities for Chemical Biology Tools and Expansion of the Ligandable Proteome
Source: ACS Chem Biol. 2023 Jan 17;18(2):285–95. doi: 10.1021/acschembio.2c00633 (PMC9942091; doi:10.1021/acschembio.2c00633)
Supplement: Supplementary file 1 — cb2c00633_si_001.pdf [file cb2c00633_si_001.pdf]

SUPPORTING INFORMATION

Profiling Sulfur(VI) Fluorides as Reactive Functionalities for Chemical Biology Tools and Expansion of the Ligandable Proteome

Katharine E. Gilbert[†‡], Aini Vuorinen[ª], Arron Aatkar[†‡], Peter Pogány[†], Jonathan Pettinger[ª], Emma K. Grant[†], Joanna M. Kirkpatrick[||], Katrin Rittinger[||], David House[†ª], Glenn A. Burley[‡]*, Jacob T. Bush[†ª]*.

[†]GlaxoSmithKline, Gunnels Wood Road, Stevenage, Hertfordshire, SG1 2NY, United Kingdom. [‡]University of Strathclyde, 295 Cathedral Street, Glasgow, G11XL, United Kingdom. [ª]Crick-GSK Biomedical LinkLabs, GlaxoSmithKline, Gunnels Wood Road, Stevenage SG1 2NY, United Kingdom. [||]The Francis Crick Institute, 1 Midland Road, London, NW1 1AT, United Kingdom.

*Corresponding authors: glenn.burley@strath.ac.uk, jacob.x.bush@gsk.com

1. Supplemental figures ... 3
2. General experimental ... 7
2.1. Solvents, reagents, and consumables ... 7
2.2. Nuclear Magnetic Resonance (NMR) spectroscopy ... 7
2.3. Liquid Chromatography-Mass Spectrometry (LC-MS) ... 7
2.4. Mass Directed Automated Preparative (MDAP) HPLC ... 7
2.5. High Performance Liquid Chromatography (HPLC) ... 8
2.6. Infrared (IR) spectroscopy ... 8
2.7. Flash column chromatography ... 8
2.8. Centrifuge ... 8
2.9. Molecular docking ... 8
2.10. Intact-Protein LC-MS ... 8
3. Synthesis of S^{VI}-F compounds ... 10
3.1. General synthetic procedures ... 10
3.2. Morpholine S^{VI}-F fragments ... 10
3.3. CAII S^{VI}-F fragment library ... 14
3.4. S^{VI}-F kinase probe precursor ... 18
3.5. S^{VI}-F kinase probe analogues ... 20
4. Intrinsic reactivity studies ... 25
4.1. Buffer composition ... 25
4.2. Hydrolysis study protocol ... 25
4.3. Amino acid reactivity study protocol ... 26
5. LUMO energy calculations ... 27
6. Protein reactivity studies ... 28
6.1. Protein stock solutions ... 28
6.2. Protein modification study protocols ... 28
6.2.1. CAII modification kinetics study ... 28
6.3. CDK2 modification study ... 28
7. Gel electrophoresis with S^{VI}-F probe analogues ... 28
8. Chemoproteomic studies with S^{VI}-F probe analogues ... 29
8.1. Acetylation of NeutrAvidin agarose resin ... 29
8.2. Chemoproteomics workflow ... 29
8.3. LC-MS/MS analysis ... 30
8.4. Data analysis ... 30
9. ^{1}H NMR spectra ... 31
10. ^{19}F NMR spectra ... 45
11. ^{13}C NMR spectra ... 59
12. References ... 73

1. Supplemental figures

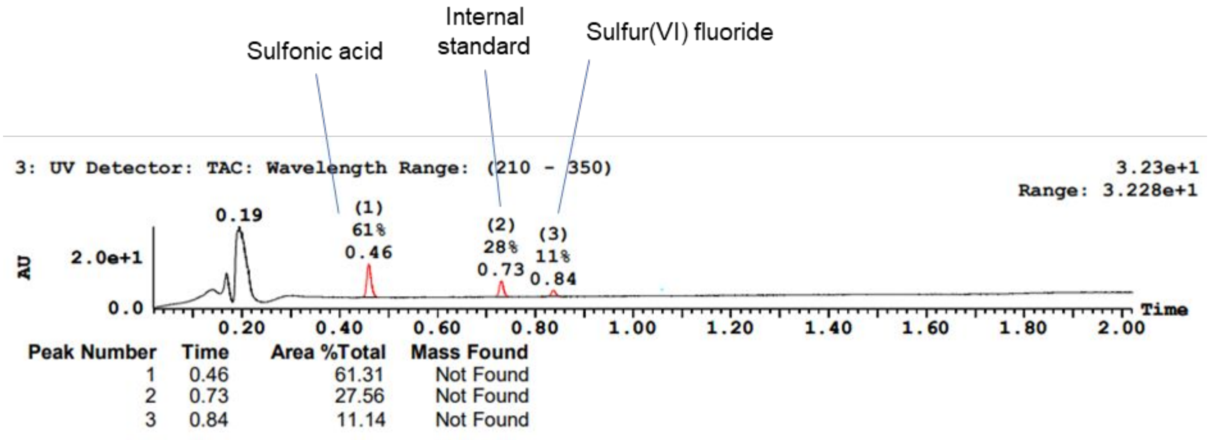

Figure S1. Example LC-MS spectra showing the conversion of sulfur(VI) fluoride compound to corresponding sulfonic acid.

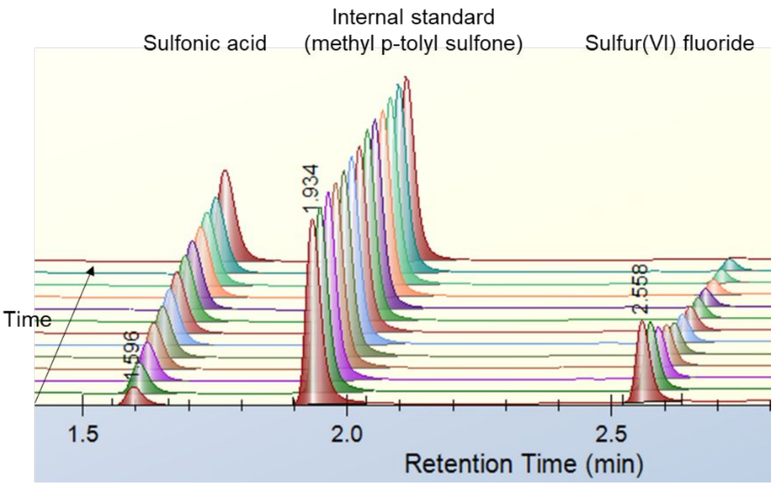

Figure S2. An example HPLC ChromView chromatogram showing the hydrolysis of sulfur(VI) fluoride compound to sulfonic acid over time.

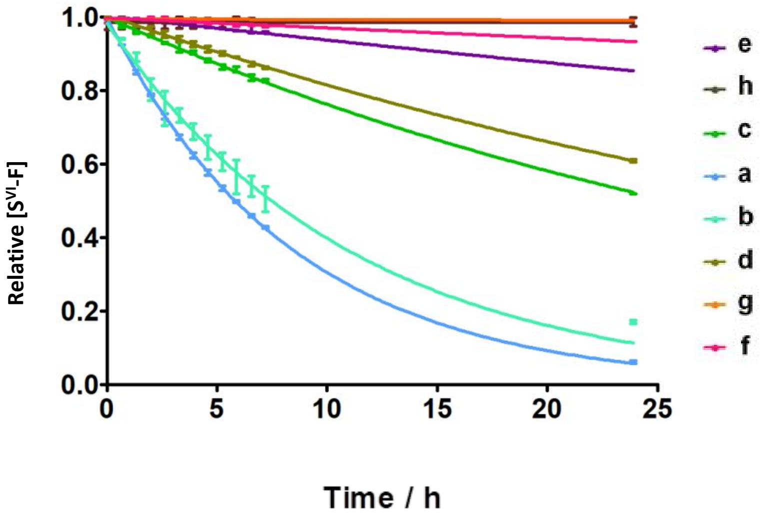

Figure S3. The hydrolysis of S^{VI}-F inhibitors **1a–h** in HEPES buffer, pH 7.0. Error bars represent duplicate data points.

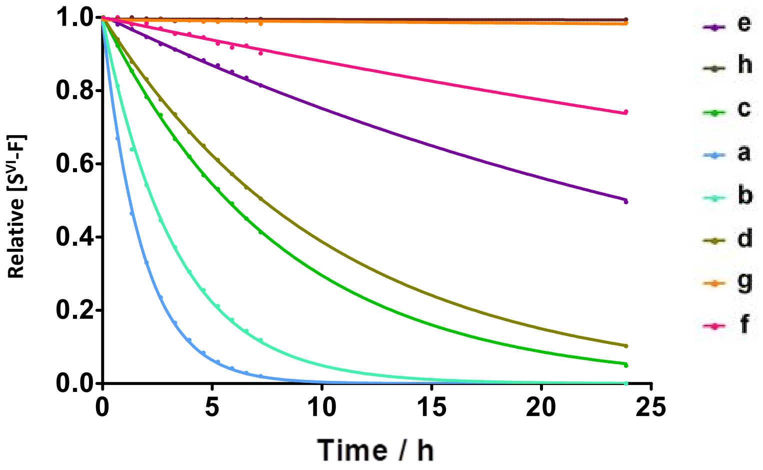

Figure S4. The hydrolysis of S^{VI}-F inhibitors **1a–h** in HEPES buffer, pH 8.0.

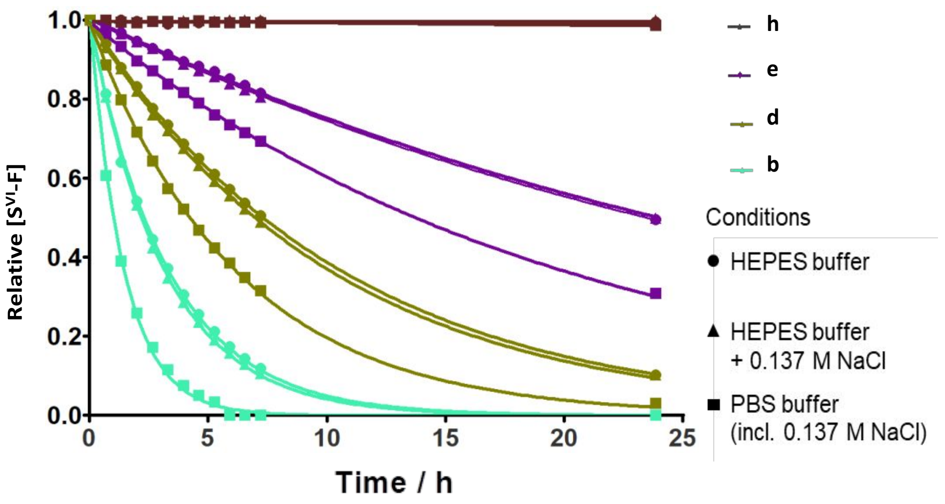

Figure S5. The hydrolysis of S^{VI}-F inhibitors **1b**, **1d**, **1e**, and **1h** under three different buffer conditions at pH 8.0 to assess the effect of NaCl upon hydrolysis rates. The standard PBS buffer contains 0.137 M NaCl.

Table S1. Experimentally measured aqueous half-lives in buffer solution (0.1 M).

Compound	Aqueous half-life / h					
	HEPES pH 7.0	HEPES pH 8.0	HEPES +0.137 M NaCl pH 8.0	PBS pH 7.0	PBS pH 8.0	Bicarbonate pH 10.0
**1a**	5.9	1.3	-	1.5	0.6	-
**1b**	7.7	2.3	2.2	2.3	1.0	-
**1c**	25.6	5.7	-	7.6	3.1	-
**1d**	33.1	7.3	7.0	9.7	4.3	-
**1e**	103	23.8	23.3	40.5	15.3	-
**1f**	248	54.2	-	90.3	26.9	0.2
**1g**	4310	1480	-	1700	612	4.7
**1h**	>4310	7990	>23.3 (high)	>1700 (4700*)	>612 (1510*)	55.6
**1i**	-	-	-	>1700 ($1.84x10^6$*)	>612 ($4.87x10^5$*)	272

*Half-lives predicted from the computational LUMO energy model as described in Section 5.

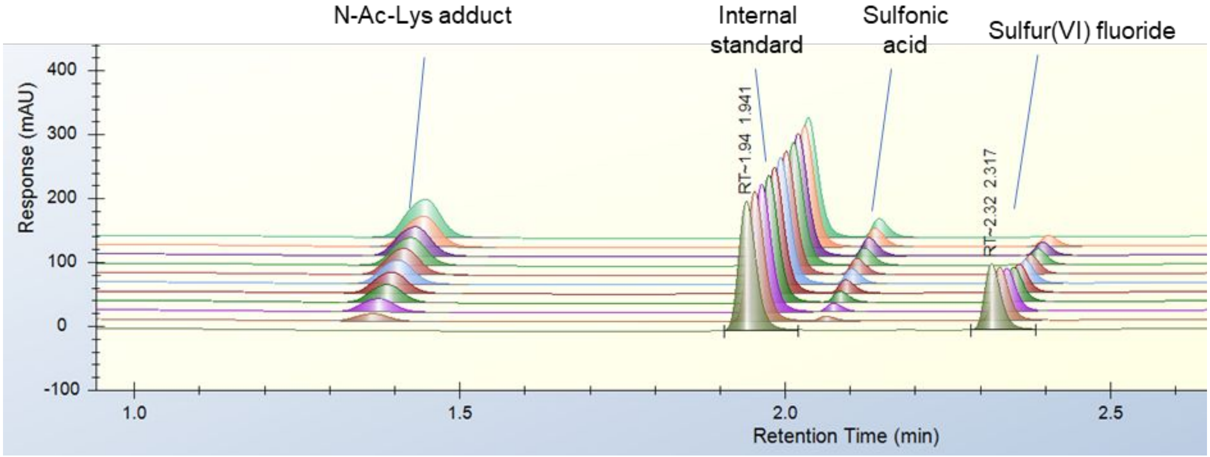

Figure S6. An example HPLC ChromView chromatogram displaying the reaction of compound **1a** with *N*-Ac-Lys amino acid in pH 8.0 PBS buffer solution over time.

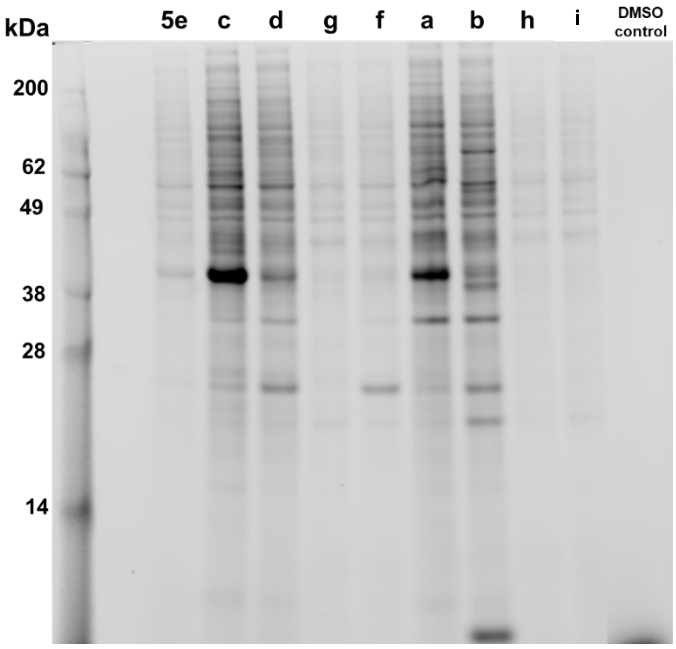

Figure S7. Gel fluorescence of compounds **5a–i** with lysate in the order as run.

2. General experimental

2.1. Solvents, reagents, and consumables

Solvents were anhydrous and reagents purchased from commercial suppliers were used as received.

2.2. Nuclear Magnetic Resonance (NMR) spectroscopy

Proton Nuclear Magnetic Resonance spectra were recorded at ambient temperature on Bruker AV-400 (^{1}H = 400 MHz, ^{13}C = 101 MHz, ^{19}F = 376 MHz) or AV-600 (^{1}H = 600 MHz, ^{13}C = 151 MHz, ^{19}F = 376 MHz) spectrometers in the stated deuterated solvent and referenced either to residual non-deuterated solvent or 0.03% (*v/v*) trimethylsilane (TMS). Chemical shifts are reported in parts per million (ppm) and coupling constants (*J*) are reported in Hertz (Hz). ^{13}C experiments were recorded with total proton decoupling.

2.3. Liquid Chromatography-Mass Spectrometry (LC-MS)

LC-MS was used to monitor reaction progress and analyse final compound purity using the solvent system as stated below. For these methods, samples were prepared in methanol unless otherwise stated. The chromatography was carried out on an Acquity UPLC CSH C-18 column (internal diameter: 50 mm × 2.1 mm, packing diameter: 1.7 µm) at 40 °C with a 0.5 µL injection volume. The UV detection was a summed signal from wavelengths between 210 nm and 350 nm. Mass detection was performed with alternate-scan positive and negative electrospray on a Waters QDa instrument, with a scan range of 100–1000 Da or 100–1200 Da (high mass range method). Scan time was 0.27 s with an inter-scan delay of 0.10 s.

Sample was eluted using a gradient shown in Table S2 with a flow rate of 1.0 mL min^{-1}.

Solvent A: 0.1% (*v/v*) solution of formic acid in water.

Solvent B: 0.1% (*v/v*) solution of formic acid in acetonitrile.

Table S2. Low pH elution gradient for LC-MS analysis.

Time (min)	Solvent A (%)	Solvent B (%)
**0.0**	97	3
**1.5**	5	95
**1.9**	5	95
**2.0**	97	3

2.4. Mass Directed Automated Preparative (MDAP) HPLC

Mass directed Autoprep was conducted on a Waters® ZQ MS using alternate scan positive and negative electrospray ionisation and a summed UV wavelength of 210–350 nm. Mass detection was over the range 150–1000 Da. The scan time was 0.5 s with an inter-scan delay of 0.2 s.

Sunfire® C18 column (100 mm × 19.0 mm, 5.00 µm packing diameter, 20.0 mL/min flow rate) using a gradient elution at ambient temperature with the mobile phases of water with 0.1% formic acid by volume (*v/v*) and acetonitrile containing 0.1% formic acid by volume (*v/v*).

The gradient of acetonitrile required to elute product was determined by the LC-MS retention time. The following methods were selected according to the retention time (t_R) of the desired material:

Table S3. Methods for MDAP purification.

Method	Flow rate / mL min^{-1}	% Acetonitrile	LC-MS t_R / min
A	40	0–30	0.40–0.65
B	40	15–55	0.65–0.90
C	40	30–85	0.90–1.16
D	40	50–99	1.16–1.40
E	40	80–99	1.40–2.00

2.5. High Performance Liquid Chromatography (HPLC)

The liquid chromatography (LC) analysis was conducted on a Waters CSH column (30 mm × 2.1 mm, particle size: 2.5 µm) at 37 °C using a 2 µL injection volume. Sample was eluted using a gradient shown in Table S4 with a flow rate of 1.0 mL min^{-1}. The UV detection wavelength was 235 nm. The total run time was 5.5 min. Solvent A: 0.05% (*v/v*) solution of trifluoroacetic acid in water. Solvent B: 0.05% (*v/v*) solution of trifluoroacetic acid in MeCN.

Table S4. Low pH elution gradient for HPLC analysis.

Time (min)	Solvent A (%)	Solvent B (%)
**0.0**	97	3
**3.7**	5	95
**4.0**	5	95
**4.1**	97	3

2.6. Infrared (IR) spectroscopy

IR spectra were recorded using a Perkin Elmer® spectrum 1 machine. Absorption maxima (ν_{max}) are reported in wavenumbers (cm^{-1}).

2.7. Flash column chromatography

Column chromatography was carried out using the Teledyne ISCO CombiFlash® Rf+ apparatus with RediSep® silica cartridges.

2.8. Centrifuge

Plates were centrifuged using a Sorvall Legend RT (401198833) model at 1000 rpm for 1 minute.

2.9. Molecular docking

Virtual molecular docking was carried out in Molecular Operating Environment (MOE) (Version 2019.0101).

CAII with compound **4a**: PDB file 2VVB (human CAII complexed with bicarbonate) was used. The docking tool was used to map **4a** in the Zn^{2+} binding site. The methods used were:

- Placement: Triangle Matcher; Score: London dG
- Refinement: Rigid Receptor; Score: GBVI/WSA dG

CDK2 with compound **5c**: PDB file 6INL (CDK2 complexed with inhibitor CVT-313) was used. The docking tool was used to map probe **5c** in the ATP binding site using CVT-313 as a template. The methods used were:

- Placement: Triangle Matcher; Score: London dG
- Refinement: Rigid Receptor; Score: GBVI/WSA dG

2.10. Intact-Protein LC-MS

Intact-protein masses were recorded by LC-MS using an Agilent G6230B time-of-flight (ToF) Accurate Mass Series mass spectrometer, interfaced with an Agilent 1290 infinity II series column oven (G7116B) and an Agilent 1290 infinity II series liquid chromatography high speed binary pump (G7120A). The protein sample was injected using an Agilent 1290 infinity II series multisampler with dual needles (Model No. G7167B) with a 10 µL injection volume and maintained at a temperature of 4 °C. Chromatography was carried out on an Agilent Bio-HPLC PLRP-S (1000 Å, 5 µm × 50 mm × 1.0 mm, PL1312-1502) reverse phase HPLC column at 70 °C. The sample was eluted at 0.5 mL min^{-1} using a gradient system from Solvent A (water, 0.2% (*v/v*) formic acid) to Solvent B (acetonitrile, 0.2% (*v/v*) formic acid) according to the conditions described in Table S5. The eluent was injected directly into an Agilent ToF mass spectrometer (Model No. G6230B) using a dual AJS ESI source and scanning between 600–3200 Da with a scan rate of 1.20 s in positive mode. The following MS parameters were used: 4000 V capillary voltage limit, 350 °C desolvation temperature, 10 L min^{-1} drying gas flow.

Table S5. Intact-protein LC-MS solvent gradient. Solvent A = water, 0.2% (*v/v*) formic acid, Solvent B = acetonitrile, 0.2% (*v/v*) formic acid.

Time (min)	Flow rate (mL min^{-1})	Solvent A (%)	Solvent B (%)
0.60	0.5	80	20
0.61	0.5	50	50
1.00	0.5	0	100
1.20	0.5	0	100
1.21	0.5	80	20

Data acquisition was carried out in 2 GHz Extended Dynamic range mode. Spectra were processed using Mass Hunter Qualitative Analysis™ B06.00 (Agilent) software with the Maximum Entropy method employed. The total ion chromatograms (TIC) were extracted (region containing protein) and the summed scans were deconvoluted (using a maximum entropy algorithm) over a *m/z* range with an expected mass range dependent on the protein.

Table S6. The deconvolution conditions for recombinant proteins studied in this work.

Protein	Expected mass range	*m/z* range
**CAII**	20000–40000	350–2000
**CDK2**	35000–40000	350–2000

The deconvoluted spectra were exported as csv files and analysed using R Studio software to generate pdf and excel files of the spectra.

The peak height for unmodified and modified protein were recorded and used to calculate percentage modification using Equation S1.

% = ((intensity of modified protein)/(intensity of protein only+(intensity of modified protein)))*100

Equation S1. Relating the percentage crosslinking to the intensities of protein and modified protein, as quantified by intact protein LC-MS.

3. Synthesis of S^{VI}-F compounds

S^{VI}-F carboxylic acid and sulfonyl chloride precursors were purchased from Enamine.

3.1. General synthetic procedures

General procedure A: nucleophilic substitution reaction to afford compounds containing S^{VI}-F electrophiles **a** and **b**.

A solution of amine (1 eq.), triethylamine (1 eq.) and sulfonyl chloride (1 eq.) was stirred in anhydrous acetonitrile (1.5 mL) at rt for 1 h. The solution was concentrated in vacuo, then purified by MDAP or by flash column chromatography (0–100% EtOAc/cyclohexane) to afford the desired product.

General procedure B: amide coupling reaction to afford compounds containing S^{VI}-F electrophiles **c**–**i**.

DIPEA (1.5 eq) was added to a stirred solution of amine (1.3 eq.), carboxylic acid (1 eq.) and HATU (1.7 eq.) in DMF (3 mL) and stirred at rt for 1 h. The solution was concentrated in vacuo, then purified by MDAP or by flash column chromatography (0–100% EtOAc/cyclohexane) to afford the desired product.

3.2. Morpholine S^{VI}-F fragments

4-(Morpholinosulfonyl)benzenesulfonyl fluoride

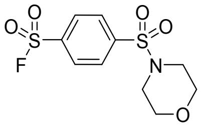

1a

Following the general procedure A, treatment of morpholine (0.020 mL, 0.232 mmol), triethylamine (0.048 mL, 0.348 mmol), 4-(chlorosulfonyl)benzenesulfonyl fluoride (60 mg, 0.232 mmol) and MeCN (1.5 mL) afforded 4-(morpholinosulfonyl)benzenesulfonyl fluoride **1a** (55.9 mg, 0.181 mmol, 78% yield) as a white solid.

ν_{max} (neat)/cm^{-1}	3110, 2919, 2865, 1459, 1407, 1351, 1265, 1214, 1161, 1095, 1069, 942, 805, 762, 723, 629, 608, 546, 515
δ_H ppm (400 MHz, $CDCl_3$)	8.25–8.18 (2 H, m), 8.06–7.98 (2 H, m), 3.81–3.73 (4 H, m), 3.13–3.05 (4 H, m)
δ_F ppm (376 MHz, $CDCl_3$)	66.02 (1 F, s)
δ_C ppm (101 MHz, $CDCl_3$)	142.8**,** 137.3, 129.4**,** 128.9**,** 66.0**,** 45.9
LC-MS	t_r = 0.96 min, 99% by UV, $[M+H]^+$ not found, poor ionisation
HRMS	($C_{10}H_{12}FNO_5S_2$) $[M+H]^+$ requires 310.0141, found $[M+H]^+$ = 310.0219

3-(Morpholinosulfonyl)benzenesulfonyl fluoride

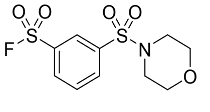

1b

Following the general procedure A, treatment of morpholine (0.022 mL, 0.252 mmol), triethylamine (0.035 mL, 0.252 mmol), 3-(chlorosulfonyl)benzenesulfonyl fluoride (65.3 mg, 0.252 mmol) and MeCN (1.5 mL), afforded 3-(morpholinosulfonyl)benzenesulfonyl fluoride **1b** (43.8 mg, 0.142 mmol, 56% yield) as a white solid.

ν_{max} (neat)/cm^{-1}	3077, 2916, 2870, 1455, 1405, 1351, 1265, 1215, 1168, 1110, 1071, 818, 772, 727, 675, 596, 512

δ_H ppm (400 MHz, $CDCl_3$)	8.41–8.39 (1 H, m) 8.31–8.27 (1 H, m) 8.19–8.15 (1 H, m) 7.93–7.87 (1 H, m), 3.81–3.76 (4 H, m), 3.11–3.06 (4 H, m)
δ_F ppm (376 MHz, $CDCl_3$)	66.36 (1 F, s)
δ_C ppm (101 MHz, $CDCl_3$)	138.3, 134.6, 134.2, 132.3, 130.9, 127.6, 66.0, 45.9
LC-MS	t_r = 0.95 min, 100% by UV, $[M+H]^+$ not found, poor ionisation
HRMS	($C_{10}H_{12}FNO_5S_2$) $[M+H]^+$ requires 310.0141, found $[M+H]^+$ = 310.0213

4-(Morpholine-4-carbonyl)benzenesulfonyl fluoride

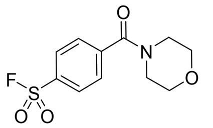

1c

Following the general procedure B, treatment of morpholine (0.030 mL, 0.344 mmol), HATU (193 mg, 0.508 mmol), 4-(fluorosulfonyl)benzoic acid (61 mg, 0.299 mmol), DMF (3 mL) and DIPEA (0.078 mL, 0.448 mmol) afforded 4-(morpholine-4-carbonyl)benzenesulfonyl fluoride **1c** (24.4 mg, 0.089 mmol, 30% yield) as a white solid.

ν_{max} (neat)/cm^{-1}	2920, 1648, 1633, 1434, 1404, 1269, 1210, 1111, 1067, 1025, 894, 841, 784, 750, 559, 597, 559, 515
δ_H ppm (400 MHz, $CDCl_3$)	8.13–8.08 (2 H, m), 7.71–7.65 (2 H, m), 3.90–3.75 (4 H, m), 3.72–3.56 (2 H, m), 3.48–3.31 (2 H, m)
δ_F ppm (376 MHz, $CDCl_3$)	65.94 (1 F, s)
δ_C ppm (101 MHz, $CDCl_3$)	167.7, 142.5, 134.1, 128.9, 128.3, 66.7
LC-MS	t_r = 0.77 min, 97% by UV, $[M+H]^+$ found: 274
HRMS	($C_{11}H_{12}FNO_4S$) [M+H]+ requires 274.0471, found [M+H]+ = 274.0550.

3-(Morpholine-4-carbonyl)benzenesulfonyl fluoride

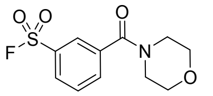

1d

Following the general procedure B, treatment of morpholine (0.032 mL, 0.360 mmol), HATU (203 mg, 0.533 mmol, 3-(fluorosulfonyl)benzoic acid (64 mg, 0.313 mmol), DMF (3 mL) and DIPEA (0.082 mL, 0.470 mmol) afforded 3-(morpholine-4-carbonyl)benzenesulfonyl fluoride **1d** (52.1 mg, 0.191 mmol, 61% yield) as a white solid.

ν_{max} (neat)/cm^{-1}	2867, 1633, 1600, 1436, 1404, 1261, 1210, 1111, 1022, 797, 724, 683, 590, 513
δ_H ppm (400 MHz, $CDCl_3$)	8.12–8.05 (2 H, m), 7.86–7.80 (1 H, m), 7.76–7.69 (1 H, m), 3.93–3.63 (6 H, m), 3.57–3.35 (2 H, m)
δ_F ppm (376 MHz, $CDCl_3$)	66.01 (1 F, s)
δ_C ppm (101 MHz, $CDCl_3$)	167.4, 137.3, 134.1, 133.96, 130.2, 129.6, 127.2, 66.7
LC-MS	t_r = 0.77 min, 100% by UV, $[M+H]^+$ found: 274
HRMS	($C_{11}H_{12}FNO_4S$) [M+H]+ requires 274.0471, found [M+H]+ = 274.0541

4-(2-Morpholino-2-oxoethyl)benzenesulfonyl fluoride

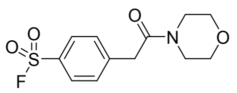

1e

Following the general procedure B, treatment of morpholine (0.023 mL, 0.264 mmol), HATU (148 mg, 0.390 mmol), 2-(4-(fluorosulfonyl)phenyl)acetic acid (50 mg, 0.229 mmol), DMF (3 mL) and DIPEA (0.060 mL, 0.344 mmol) afforded 4-(2-morpholino-2-oxoethyl)benzenesulfonyl fluoride **1e** (20.4 mg, 0.071 mmol, 31% yield) as a white solid.

ν_{max} (neat)/cm^{-1}	2904, 2861, 1633, 1440, 1403, 1303, 1205, 1183, 1036, 779, 734, 677, 577, 535
δ_H ppm (400 MHz, $CDCl_3$)	8.03–7.97 (2 H, m), 7.63–7.53 (2 H, m), 3.88–3.83 (2 H, m), 3.74–3.64 (6 H, m) 3.52–3.49 (2 H, m)
δ_F ppm (376 MHz, $CDCl_3$)	66.10 (1 F, s)
δ_C ppm (101 MHz, $CDCl_3$)	167.9, 143.4, 131.6, 130.4, 128.7, 66.8, 46.4, 42.4, 40.1
LC-MS	t_r = 0.81 min, 100% by UV, $[M+H]^+$ found: 288
HRMS	[$C_{12}H_{14}FNO_4S$] [M+H]+ requires 288.0627, found [M+H]+ = 288.0692

4-Methoxy-3-(morpholine-4-carbonyl)benzenesulfonyl fluoride

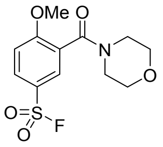

1f

Following the general procedure B, treatment of morpholine (0.026 mL, 0.295 mmol), HATU (166 mg, 0.436 mmol), 2-(4-(fluorosulfonyl)phenyl)acetic acid (60 mg, 0.256 mmol), DMF (3 mL) and DIPEA (0.067 mL, 0.384 mmol) afforded 4-methoxy-3-(morpholine-4-carbonyl)benzenesulfonyl fluoride **1f** (36.2 mg, 0.119 mmol, 47% yield) as a white solid.

ν_{max} (neat)/cm^{-1}	3323, 1600, 1470, 1429, 1390, 1269, 1089, 1030, 1012, 980, 814, 785, 670, 554
δ_H ppm (400 MHz, $CDCl_3$)	8.05 (1 H, dd, *J*=8.9, 2.5 Hz), 7.94 (1 H, d, *J*=2.5 Hz), 7.13 (1 H, d, *J*=8.9 Hz), 4.00 (3 H, s), 3.87–3.77 (4 H, m), 3.68–3.62 (2 H, m), 3.30–3.23 (2 H, m)
δ_F ppm (376 MHz, $CDCl_3$)	67.30 (1 F, s)
δ_C ppm (101 MHz, $CDCl_3$)	164.8, 131.9, 129.1, 127.0, 125.2, 125.0, 111.7, 66.7, 56.5, 47.3, 42.3
LC-MS	t_r = 0.78 min, 100% by UV, $[M+H]^+$ found: 304
HRMS	($C_{12}H_{14}FNO_5S$) [M+H]+ requires 304.0577, found [M+H]+ = 304.0652.

4-(Morpholine-4-carbonyl)phenyl sulfurofluoridate

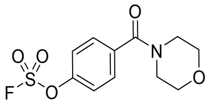

1g

Following the general procedure B, treatment of morpholine (0.088 mL, 1.00 mmol), HATU (562 mg, 1.48 mmol), crude 4-((fluorosulfonyl)oxy)benzoic acid (191.5 mg, 0.870 mmol), DMF (3 mL) and DIPEA (0.228 mL, 1.31 mmol) afforded 4-(morpholine-4-carbonyl)phenyl sulfurofluoridate **1g** (75.1 mg, 0.260 mmol, 30% yield) as a white solid.

ν_{max} (neat)/cm^{-1}	2873, 1644, 1634, 1434, 1445, 1273, 1261, 1232, 1135, 1111, 1013, 916, 810, 756, 605, 577, 542
δ_H ppm (400 MHz, $CDCl_3$)	7.59–7.54 (2 H, m), 7.46–7.40 (2 H, m), 4.01–3.61 (6 H, m) 3.59–3.30 (2 H, m)
δ_F ppm (376 MHz, $CDCl_3$)	38.29 (1 F, s)
δ_C ppm (101 MHz, $CDCl_3$)	168.4, 150.6, 136.0, 129.5, 121.3, 66.8
LC-MS	t_r = 0.84 min, 100% by UV, $[M+H]^+$ found: 290
HRMS	($C_{11}H_{12}FNO_5S$) [M+H]+ requires 290.042, found [M+H]+ = 290.0489

5-(Morpholine-4-carbonyl)-1H-pyrrole-3-sulfonyl fluoride

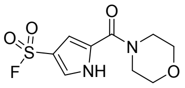

1h

Following the general procedure B, treatment of morpholine (0.026 mL, 0.297 mmol), HATU (167 mg, 0.438 mmol), 4-(fluorosulfonyl)-1H-pyrrole-2-carboxylic acid (49.8 mg, 0.258 mmol), DMF (3 mL) and DIPEA (0.068 mL, 0.387 mmol) afforded 5-(morpholine-4-carbonyl)-1H-pyrrole-3-sulfonyl fluoride **1h** (28.9 mg, 0.110 mmol, 43% yield) as a white solid.

ν_{max} (neat)/cm^{-1}	3188, 2919, 1593, 1549, 1464, 1435, 1400, 1151, 1194, 1130, 1108, 1029, 961, 889, 796, 741, 616, 598, 535, 509
δ_H ppm (400 MHz, $CDCl_3$)	10.93 (1 H, br s), 7.69–7.60 (1 H, m), 6.96–6.93 (1 H, m), 3.87 (4 H, m), 3.82–3.77 (4 H, m)
δ_F ppm (376 MHz, $CDCl_3$)	69.98 (1 F, s)
δ_C ppm (101 MHz, $CDCl_3$)	159.9, 126.6, 126.4, 116.3, 111.5, 66.6
LC-MS	t_r = 0.71 min, 100% by UV, $[M+H]^+$ found: 263
HRMS	($C_9H_{11}FN_2O_4S$) [M+H]+ requires 263.0424, found [M+H]+ = 263.0493

3-(Morpholine-4-carbonyl)azetidine-1-sulfonyl fluoride

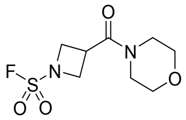

1i

Following the general procedure B, treatment of morpholine (0.033 mL, 0.379 mmol, HATU (213 mg, 0.560 mmol), 1-(fluorosulfonyl)azetidine-3-carboxylic acid (60.3 mg, 0.329 mmol), DMF (3 mL) and DIPEA (0.086 mL, 0.494 mmol) afforded 3-(morpholine-4-carbonyl)azetidine-1-sulfonyl fluoride **1i** (67.0 mg, 0.266 mmol, 81% yield) as a white solid.

ν_{max} (neat)/cm^{-1}	2992, 2901, 2863, 1639, 1450, 1411, 1277, 1243, 1210, 1114, 1010, 844, 753, 740, 651, 630, 559, 529
δ_H ppm (400 MHz, $CDCl_3$)	4.52–4.45 (2 H, m), 4.32–4.23 (2 H, m), 3.76–3.63 (7 H, m), 3.33–3.25 (2 H, m)
δ_F ppm (376 MHz, $CDCl_3$)	29.89 (1 F, s)
δ_C ppm (101 MHz, $CDCl_3$)	167.9, 66.7, 54.4, 45.6, 42.5
LC-MS	t_r = 0.65 min, 100% by UV, $[M+H]^+$ found: 253
HRMS	($C_8H_{13}FN_2O_4S$) $[M+H]^+$ requires 253.0580, found $[M+H]^+$ = 253.0710

3.3. CAII S^{VI}-F fragment library

4-(*N*-(1-(3-Sulfamoylphenyl)ethyl)sulfamoyl)benzenesulfonyl fluoride

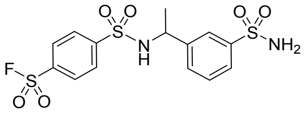

2a

Following the general procedure A, treatment 3-(1-aminoethyl)benzenesulfonamide (24.8 mg, 0.124 mmol) with 4-(chlorosulfonyl)benzenesulfonyl fluoride (32.0 mg, 0.124 mmol) and triethylamine (17.2 µL, 0.124 mmol) afforded 4-(*N*-(1-(3-sulfamoylphenyl)ethyl)sulfamoyl)benzenesulfonyl fluoride **2a** (29.5 mg, 0.070 mmol, 56% yield) as a white solid.

ν_{max} (neat)/cm^{-1}	3264, 3103, 1414, 1326, 1285, 1214, 1154, 1083, 1028, 909, 871, 783, 745, 700, 608, 595, 520
δ_H ppm (400 MHz, DMSO-d$_6$))	8.81 (1 H, d, *J*=7.9 Hz) 8.20–8.14 (2 H, m) 7.95–7.91 (2 H, m) 7.72–7.70 (1 H, m) 7.61–7.56 (1 H, m) 7.38–7.34 (2 H, m) 7.32–7.28 (2 H, m) 4.61–4.50 (1 H, m) 1.31 (3 H, d, *J*=6.9 Hz)
δ_F ppm (471 MHz, DMSO-d$_6$))	66.09 (1 F, s)
δ_C ppm (101 MHz, DMSO-d$_6$)	148.6, 144.5, 143.9, 134.9, 134.7, 129.7, 129.3, 128.5, 124.8, 124.0, 53.4, 23.6
LC-MS	t_r = 0.90 min, 100% by UV, [M-H]$^-$ found: 421
HRMS	($C_{14}H_{15}FN_2O_6S_3$) [M+H]+ requires 423.0076, found [M+H]$^+$ = 423.0151

3-(*N*-(1-(3-Sulfamoylphenyl)ethyl)sulfamoyl)benzenesulfonyl fluoride

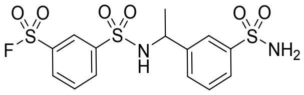

2b

Following the general procedure A, treatment of 3-(1-aminoethyl)benzenesulfonamide (23.0 mg, 0.116 mmol) with 3-(chlorosulfonyl)benzenesulfonyl fluoride (30.0 mg, 0.116 mmol) and triethylamine (16.2 µL, 0.116 mmol) afforded 3-(*N*-(1-(3-sulfamoylphenyl)ethyl)sulfamoyl)benzenesulfonyl fluoride **2b** (30.5 mg, 0.072 mmol, 62% yield) as a white solid.

ν_{max} (neat)/cm^{-1}	3306, 3280, 3212, 2604, 2497,1410, 1327, 1214, 1156, 1103, 1078, 996, 878, 814, 797, 711, 676, 616, 590, 574, 520
δ_H ppm (400 MHz, DMSO-d$_6$))	8.84 (1 H, d, *J*=8.4 Hz) 8.27–8.20 (2 H, m) 8.12–8.06 (1 H, m) 7.85–7.79 (1 H, m) 7.67–7.64 (1 H, m) 7.60–7.56 (1 H, m) 7.40–7.36 (1 H, m) 7.34–7.28 (3 H, m) 4.62–4.52 (1 H, m) 1.30 (3 H, d, *J*=6.9 Hz)
δ_F ppm (471 MHz, DMSO-d$_6$))	66.71 (1 F, s)
δ_C ppm (101 MHz, DMSO-d$_6$)	144.6, 143.7, 134.2, 132.8, 132.6, 132.2, 132.1, 129.8, 129.3, 126.3, 124.9, 123.9, 45.9, 23.6
LC-MS	t_r = 0.90 min, 100% by UV, [M-H]$^-$ found: 421
HRMS	($C_{14}H_{15}FN_2O_6S_3$) [M+H]$^+$ requires 423.0076, found [M+H]+ = 423.0154

4-((1-(3-Sulfamoylphenyl)ethyl)carbamoyl)benzenesulfonyl fluoride

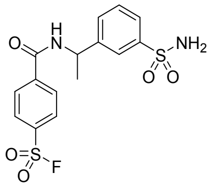

2c

Following the general procedure B, treatment of 3-(1-aminoethyl)benzenesulfonamide (33.3 mg, 0.167 mmol) with 4-(fluorosulfonyl)benzoic acid (30.0 mg, 0.147 mmol), HATU (95.0 mg, 0.250 mmol) and DIPEA (0.039 mL, 0.220 mmol) afforded 4-((1-(3-sulfamoylphenyl)ethyl)carbamoyl)benzenesulfonyl fluoride **2c** (38.2 mg, 0.099 mmol, 67% yield) as a white solid.

ν_{max} (neat)/cm^{-1}	3244, 1646, 1537, 1407, 1330, 1211, 1157, 1015, 843, 781, 731, 693, 611, 589, 513
δ_H ppm (400 MHz, DMSO-d$_6$))	9.34 (1 H, d, *J*=7.4 Hz) 8.31–8.26 (2 H, m) 8.25–8.21 (2 H, m) 7.90–7.85 (1 H, m) 7.76–7.70 (1 H, m) 7.66–7.61 (1 H, m) 7.59–7.52 (1 H, m) 7.34 (2 H, br s) 5.31–5.19 (1 H, m) 1.54 (3 H, d, *J*=6.9 Hz)
δ_F ppm (471 MHz, DMSO-d$_6$))	66.15 (1 F, s)
δ_C ppm (101 MHz, DMSO-d$_6$)	164.3, 145.9, 144.7, 141.7, 134.2, 130.0, 129.8, 129.5, 129.1, 124.7, 123.6, 49.3, 22.4
LC-MS	t_r = 0.89 min, 100% by UV, [M+H]$^+$ found: 387
HRMS	($C_{15}H_{15}FN_2O_5S_2$) [M+H]+ requires 387.0406, found [M+H]+ = 387.0477.

3-((1-(3-Sulfamoylphenyl)ethyl)carbamoyl)benzenesulfonyl fluoride

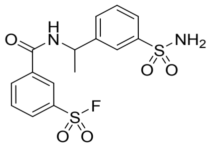

2d

Following the general procedure B, treatment of 3-(1-aminoethyl)benzenesulfonamide (38.3 mg, 0.191 mmol) with 3-(fluorosulfonyl)benzoic acid (30.0 mg, 0.147 mmol), HATU (95.0 mg, 0.250 mmol) and DIPEA (38.0 µL, 0.220 mmol) afforded 3-((1-(3-sulfamoylphenyl)ethyl)carbamoyl)benzenesulfonyl fluoride **2d** (18.6 mg, 0.048 mmol, 33% yield) as a white solid.

ν_{max} (neat)/cm^{-1}	3263, 1641, 1538, 1409, 1309, 1211, 1153, 1089, 899, 784, 737, 679, 588, 520
δ_H ppm (400 MHz, DMSO-d$_6$))	9.37 (1 H, d, *J*=7.4 Hz) 8.66–8.60 (1 H, m) 8.48–8.41 (1 H, m) 8.36–8.30 (1 H, m) 7.97–7.90 (1 H, m) 7.89–7.85 (1 H, m) 7.76–7.70 (1 H, m) 7.65–7.62 (1 H, m) 7.58–7.53 (1 H, m) 7.35 (2 H, br s) 5.32–5.19 (1 H, m) 1.55 (3 H, d, *J*=6.9 Hz)
δ_F ppm (471 MHz, DMSO-d$_6$))	66.43 (1 F, s)
δ_C ppm (101 MHz, DMSO-d$_6$)	163.6, 145.9, 144.7, 136.4, 136.0, 132.3, 131.4, 131.3, 130.1, 129.5, 127.4, 124.7, 123.6, 49.4, 22.4
LC-MS	t_r = 0.90 min, 96% by UV, [M+H]$^+$ found: 387
HRMS	($C_{15}H_{15}FN_2O_5S_2$) [M+H]+ requires 387.0406, found [M+H]+ = 387.0478.

4-(2-Oxo-2-((1-(3-sulfamoylphenyl)ethyl)amino)ethyl)benzenesulfonyl fluoride

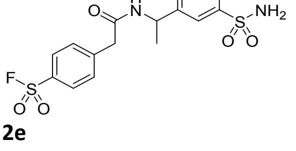

2e

Following the general procedure B, treatment of 3-(1-aminoethyl)benzenesulfonamide (33.3 mg, 0.179 mmol) with 2-(4-(fluorosulfonyl)phenyl)acetic acid (30.0 mg, 0.137 mmol), HATU (89.0 mg, 0.234 mmol) and DIPEA (33.0 µL, 0.206 mmol) afforded 4-(2-oxo-2-((1-(3-sulfamoylphenyl)ethyl)amino)ethyl)benzenesulfonyl fluoride **2e** (33.1 mg, 0.083 mmol, 60% yield) as a white solid.

ν_{max} (neat)/cm^{-1}	3256, 1638, 1538, 1411, 1310, 1209, 1156, 1098, 902, 785, 735, 684, 590, 533
δ_H ppm (400 MHz, DMSO-d$_6$))	8.80 (1 H, d, *J*=7.4 Hz) 8.10–8.03 (2 H, m) 7.81–7.78 (1 H, m) 7.73–7.69 (1 H, m) 7.68–7.64 (2 H, m) 7.55–7.50 (2 H, m) 7.34 (2 H, br s) 5.02–4.90 (1 H, m) 3.70 (2 H, s) 1.40 (3 H, d, *J*=6.9 Hz)
δ_F ppm (471 MHz, DMSO-d$_6$))	66.63 (1 F, s)
δ_C ppm (101 MHz, DMSO-d$_6$)	168.5, 146.3, 146.0, 144.7, 131.5, 129.9, 129.5, 128.8, 124.6, 123.4, 48.5, 22.7
LC-MS	t_r = 0.87 min, 95% by UV, [M+H]$^+$ found: 401
HRMS	($C_{16}H_{17}FN_2O_5S_2$) [M+H]+ requires 401.0563, found [M+H]+ = 401.0627.

4-Methoxy-3-((1-(3-sulfamoylphenyl)ethyl)carbamoyl)benzenesulfonyl fluoride

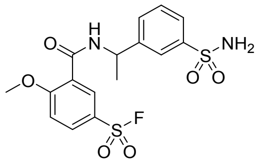

2f

Following the general procedure B, treatment of 3-(1-aminoethyl)benzenesulfonamide (33.3 mg, 0.167 mmol) with 5-(fluorosulfonyl)-2-methoxybenzoic acid (30.0 mg, 0.128 mmol), HATU (83.0 mg, 0.218 mmol) and DIPEA (0.034 mL, 0.192 mmol) afforded 4-methoxy-3-((1-(3-sulfamoylphenyl)ethyl)carbamoyl)benzenesulfonyl fluoride **2f** (35.0 mg, 0.084 mmol, 66% yield) as a white solid.

ν_{max} (neat)/cm^{-1}	3222, 1643, 1596, 1529, 1483, 1401, 1330, 1207, 1156, 1011, 904, 763, 693, 569, 512
δ_H ppm (400 MHz, DMSO-d$_6$))	8.87 (1 H, d, *J*=7.9 Hz) 8.24 (1 H, dd, *J*=9.4, 2.5 Hz) 8.11 (1 H, d, *J*=2.5 Hz) 7.92–7.88 (1 H, m) 7.76–7.72 (1 H, m) 7.66–7.61 (1 H, m) 7.60–7.54 (1 H, m) 7.50 (1 H, d, *J*=9.4 Hz) 7.35 (2 H, br s) 5.26–5.15 (1 H, m) 4.04 (3 H, s) 1.49 (3 H, d, *J*=6.9 Hz)
δ_F ppm (471 MHz, DMSO-d$_6$))	67.86 (1 F, s)
δ_C ppm (101 MHz, DMSO-d$_6$)	163.0, 145.8, 144.7, 133.0, 130.3, 130.0, 129.5, 127.0, 124.6, 123.5, 122.9, 122.7, 114.3, 57.5, 49.1, 22.7
LC-MS	t_r = 0.91 min, 94% by UV, [M+H]$^+$ found: 417
HRMS	($C_{16}H_{17}FN_2O_6S_2$) [M+H]+ requires 417.0512, found [M+H]+ = 417.0589.

4-((1-(3-Sulfamoylphenyl)ethyl)carbamoyl)phenyl sulfurofluoridate

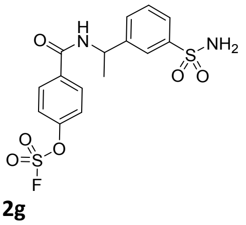

2g

Following the general procedure B, treatment of 3-(1-aminoethyl)benzenesulfonamide (35.5 mg, 0.177 mmol) with 4-((fluorosulfonyl)oxy)benzoic acid (30.0 mg, 0.136 mmol), HATU (88.0 mg, 0.232 mmol) and DIPEA (33.0 µL, 0.204 mmol) afforded a white solid. The white solid was purified by MDAP to afford 4-((1-(3-sulfamoylphenyl)ethyl)carbamoyl)phenyl sulfurofluoridate **2g** (29.0 mg, 0.072 mmol, 53% yield) as a white solid.

ν_{max} (neat)/cm^{-1}	3267, 1642, 1537, 1493, 1448, 1328, 1233, 1142, 912, 811, 693, 583, 541
δ_H ppm (400 MHz, DMSO-d$_6$))	9.13 (1 H, d, *J*=7.9 Hz) 8.15–8.03 (2 H, m) 7.87 (1 H, m) 7.77–7.70 (3 H, m) 7.66–7.60 (1 H, m) 7.58–7.51 (1 H, m) 7.35 (2 H, br s) 5.28–5.17 (1 H, m) 1.52 (3 H, d, *J*=6.9 Hz)
δ_F ppm (471 MHz, DMSO-d$_6$))	39.26 (1 F, s)
δ_C ppm (101 MHz, DMSO-d$_6$)	164.7, 151.7, 146.2, 144.7, 135.6, 130.6, 130.0, 129.5, 124.6, 123.5, 121.6, 49.2, 22.5

LC-MS	t_r = 0.93 min, 95% by UV, $[M+H]^+$ found: 403
HRMS	($C_{15}H_{15}FN_2O_6S_2$) [M+H]+ requires 403.0356, found [M+H]+ = 403.0427.

5-((1-(3-Sulfamoylphenyl)ethyl)carbamoyl)-1H-pyrrole-3-sulfonyl fluoride

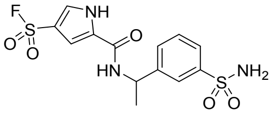

2h

Following the general procedure B, treatment of 3-(1-aminoethyl)benzenesulfonamide (40.4 mg, 0.202 mmol) with 4-(fluorosulfonyl)-1H-pyrrole-2-carboxylic acid (30.0 mg, 0.155 mmol), HATU (100 mg, 0.264 mmol) and DIPEA (0.042 mL, 0.233 mmol) afforded a white solid. The white solid was purified by MDAP to afford 5-((1-(3-sulfamoylphenyl)ethyl)carbamoyl)-1H-pyrrole-3-sulfonyl fluoride **2h** (40.7 mg, 0.108 mmol, 70% yield) as a white solid.

ν_{max} (neat)/cm^{-1}	3330, 3257, 1638, 1538, 1413, 1323, 1311, 1209, 1157, 1099, 903, 841, 782, 734, 685, 589, 535, 501
δ_H ppm (400 MHz, DMSO-d$_6$))	13.04 (1 H, br s) 8.88 (1 H, d, *J*=7.9 Hz) 7.98–7.93 (1 H, m) 7.86–7.83 (1 H, m) 7.74–7.70 (1 H, m) 7.62–7.57 (2 H, m) 7.57–7.52 (1 H, m) 7.34 (2 H, br s) 5.23–5.15 (1 H, m) 1.50 (3 H, d, *J*=6.9 Hz)
δ_F ppm (471 MHz, DMSO-d$_6$))	71.11 (1 F, s)
δ_C ppm (101 MHz, DMSO-d$_6$)	158.6, 146.0, 144.7, 130.0, 129.6, 129.5, 128.4, 124.7, 124.4, 123.5, 110.7, 48.5, 22.5
LC-MS	t_r = 0.82 min, 98% by UV, $[M+H]^+$ found: 376
HRMS	($C_{13}H_{14}FN_3O_5S_2$) [M+H]+ requires 376.0359, found [M+H]+ = 376.0424.

3-((1-(3-Sulfamoylphenyl)ethyl)carbamoyl)azetidine-1-sulfonyl fluoride

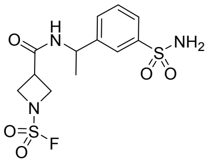

2i

Following the general procedure B, treatment of 3-(1-aminoethyl)benzenesulfonamide (42.6 mg, 0.213 mmol) with 1-(fluorosulfonyl)azetidine-3-carboxylic acid (30.0 mg, 0.164 mmol), HATU (106 mg, 0.278 mmol) and DIPEA (0.043 mL, 0.246 mmol) afforded 3-((1-(3-sulfamoylphenyl)ethyl)carbamoyl)azetidine-1-sulfonyl fluoride **2i** (24.5 mg, 0.067 mmol, 41% yield) as a white solid.

ν_{max} (neat)/cm^{-1}	3371, 3295, 3197, 1650, 1549, 1416, 1335, 1202, 1152, 992, 844, 753, 692, 646, 597, 528, 506, 528, 506
δ_H ppm (400 MHz, $CDCl_3$))	8.69 (1 H, d, *J*=7.9 Hz) 7.78–7.75 (1 H, m) 7.74–7.69 (1 H, m) 7.56–7.51 (2 H, m) 7.34 (2 H, br s) 5.06–4.96 (1 H, m) 4.36–4.27 (2 H, m) 4.27–4.21 (1 H, m) 4.21–4.15 (1 H, m) 3.61–3.51 (1 H, m) 1.39 (3 H, d, *J*=6.9 Hz)
δ_F ppm (471 MHz, $CDCl_3$)	30.64 (1 F, s)
δ_C ppm (101 MHz, $CDCl_3$)	169.3, 145.8, 144.7, 129.9, 129.5, 124.6, 123.4, 55.5, 48.6, 32.4, 22.6
LC-MS	t_r = 0.74 min, 100% by UV, $[M+H]^+$ found: 366
HRMS	($C_{12}H_{16}FN_3O_5S_2$) [M+H]+ requires 366.0515, found [M+H]+ = 366.0584.

Additional S^{VI}-F fragments were custom synthesised by Enamine: **3a,c–e, 4a,c–e**.

3.4. S^{VI}-F kinase probe precursor

Methyl 2-chloro-6-((5-cyclopropyl-1H-pyrazol-3-yl)amino)pyrimidine-4-carboxylate

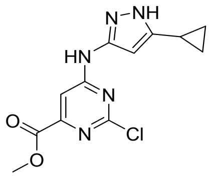

S1

To a solution of 5-cyclopropyl-1H-pyrazol-3-amine (10.00 g, 81.0 mmol) in THF (400 mL) at 0 °C (ice bath), was added methyl 2,6-dichloropyrimidine-4-carboxylate (16.81 g, 81.0 mmol) and DIPEA (15.6 mL, 89.0 mmol). The solution was allowed to warm to rt and stirred for 30 min, then was concentrated in vacuo and redissolved in MeOH. The solution was stirred at 80 °C for 30 min and a white precipitate formed. The solution was allowed to cool to rt, then to 0 °C (ice bath) for 1 hr. The mixture was filtered in vacuo, then the solid washed with MeOH, then dried under vacuum to afford methyl 2-chloro-6-((5-cyclopropyl-1H-pyrazol-3-yl)amino)pyrimidine-4-carboxylate **S1** (15.75 g, 53.6 mmol, 66% yield) as a yellow solid.

δ_H ppm (400 MHz, DMSO-d$_6$)	12.25 (1 H, br s), 10.80–10.62 (1 H, m), 8.28 (0.5 H, s), 7.34 (s, 0.5 H), 6.38 (0.5 H, s), 5.67 (0.5 H, s), 3.87 (3 H, s), 1.95–1.88 (1 H, m), 1.01–0.88 (2 H, m), 0.76–0.64 (2 H, m)
δ_C ppm (101 MHz, DMSO-d$_6$)	164.1, 162, 147.0, 107.5, 105.4, 94.0, 93.0, 53.3, 8.2, 7.2
LC-MS	t_r = 0.83 min, 100% by UV, $[M+H]^+$ found: 294

2-Chloro-6-((5-cyclopropyl-1H-pyrazol-3-yl)amino)pyrimidine-4-carboxylic acid

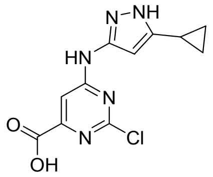

S2

To a solution of methyl 2-chloro-6-((5-cyclopropyl-1H-pyrazol-3-yl)amino)pyrimidine-4-carboxylate (115 mg, 0.39 mmol) **S1** in 1,4-dioxane (2 mL) was added aq. sodium hydroxide (2 M) (0.78 mL, 1.56 mmol) and the solution stirred at rt for 10 min. The mixture was acidified to pH 4 with aq. HCl (1 M) and a white precipitate formed. This was filtered and the solid washed with water, then dried under vacuum overnight to afford 2-chloro-6-((5-cyclopropyl-1H-pyrazol-3-yl)amino)pyrimidine-4-carboxylic acid **S2** (107 mg, 0.384 mmol, 98% yield) as an off-white solid.

δ_H ppm (400 MHz, DMSO-d$_6$)	13.53 (1 H, br s) 12.30 (1 H, br s) 10.97–10.40 (1 H, m) 8.24 (0.5 H, br s) 7.35 (0.5 H, br s) 6.37 (0.5 H, br s) 5.69 (0.5 H, br s) 2.00–1.83 (1 H, m) 1.00–0.90 (2 H, m) 0.76–0.65 (2 H, m)
LC-MS	t_r = 0.77 min, 94% by UV, $[M+H]^+$ found: 279

2-Chloro-6-((5-cyclopropyl-1H-pyrazol-3-yl)amino)-*N*-(prop-2-yn-1-yl)pyrimidine-4-carboxamide

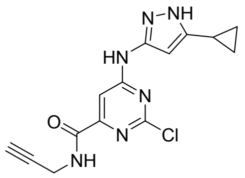

S3

To a mixture of **S2** (1.80 g, 6.44 mmol), prop-2-yn-1-amine (0.41 mL, 6.44 mmol), and DIPEA (4.50 mL, 25.7 mmol) in anhydrous DMF (32 mL), was added HATU (3.18 g, 8.37 mmol) over 10 min. The reaction was stirred at room temperature under nitrogen for 1 h. The mixture was diluted with water (50 mL) and extracted with EtOAc (100 mL). The organic phase was dried (frit) and concentrated in vacuo to afford a yellow solid. The yellow solid was purified by flash column chromatography to yield a yellow solid. Product **S3** taken through crude to next step.

Tert-butyl 4-(4-((5-cyclopropyl-1H-pyrazol-3-yl)amino)-6-(prop-2-yn-1-ylcarbamoyl)pyrimidin-2-yl)piperazine-1-carboxylate

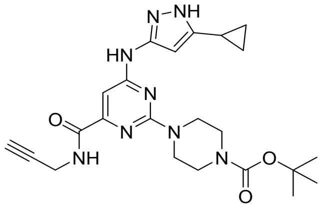

S4

To a solution of **S3** (0.894 g, 2.82 mmol) in DMF (15 mL), was added *N*-boc-piperazine (1.58 g, 8.47 mmol) and the solution was stirred at 100 °C for 2 h. The solution was concentrated in vacuo, then the resuting residue was dissolved in EtOAc (30 mL) and washed with sat. aq. ammonium chloride (30 mL) and brine (10 mL). The solution was dried (hydrophobic frit), concentrated in vacuo, then the residue purified by reverse phase column chromatography (5–100% MeCN/H_2O with 0.1% formic acid) to afford tert-butyl 4-(4-((5-cyclopropyl-1H-pyrazol-3-yl)amino)-6-(prop-2-yn-1-ylcarbamoyl)pyrimidin-2-yl)piperazine-1-carboxylate **S4** (287 mg, 0.615 mmol, 32% yield) as a pale brown solid. Impure fractions kept to side.

δ_H ppm (400 MHz, DMSO-d_6)	12.01 (1 H, s) 9.75 (1 H, br s) 8.92 (1 H, t, *J*=5.9 Hz) 6.88 (1 H, br s) 6.09 (1 H, br s) 4.02 (2 H, dd, *J*=5.9, 2.5 Hz) 3.83–3.77 (4 H, m) 3.44–3.40 (4 H, m) 3.09 (1 H, t, *J*=2.5 Hz), 1.95–1.87 (1 H, m) 1.45 (9 H, s) 0.97–0.91 (2 H, m) 0.72–0.67 (2 H, m)
δ_C ppm (101 MHz, DMSO-d_6)	154.5, 110.0, 93.2, 81.7, 79.5, 73.0, 43.8, 28.6, 8.3, 7.3
LC-MS	t_r = 1.10 min, 98% by UV, $[M+H]^+$ found: 467

6-((5-Cyclopropyl-1H-pyrazol-3-yl)amino)-2-(piperazin-1-yl)-*N*-(prop-2-yn-1-yl)pyrimidine-4-carboxamide trifluoroacetic acid salt

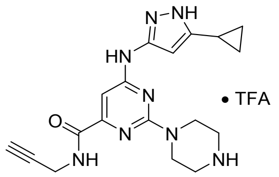

S5

To a solution of **S4** (270 mg, 0.579 mmol) in DCM (8 mL) was added trifluoroacetic acid (TFA) (3 mL, 39 mmol) and the reaction stirred at rt for 30 min. The mixture was concentrated in vacuo to afford 6-((5-cyclopropyl-1H-pyrazol-3-yl)amino)-2-(piperazin-1-yl)-*N*-(prop-2-yn-1-yl)pyrimidine-4-carboxamide trifluoroacetic acid salt **S5** (278 mg, 0.578 mmol, >99% yield) as a pale brown solid.

δ_H ppm (400 MHz, DMSO-d$_6$)	9.88 (1 H, br s) 8.98 (1 H, t, *J*=5.9 Hz) 8.85 (1 H, br s) 6.98 (1 H, br s) 6.13 (1 H, br s) 4.10–3.91 (6 H, m) 3.30–3.15 (4 H, m) 3.10 (1 H, t, *J*=2.5 Hz) 1.90 (1 H, m) 0.97–0.90 (2 H, m) 0.74–0.67 (2 H, m)
δ_C ppm (101 MHz, DMSO-d$_6$)	163.9, 160.8, 158.7, 156.9, 93.3, 81.7, 73.1, 51.4, 43.1, 41.1, 28.7, 8.3, 7.4
LC-MS	t_r = 0.47 min, 98% by UV, $[M+H]^+$ found: 367

3.5. S^{VI}-F kinase probe analogues

NB. Some peaks are missing from the ^{13}C NMR spectra of the compounds below.

4-((4-(4-((5-Cyclopropyl-1H-pyrazol-3-yl)amino)-6-(prop-2-yn-1-ylcarbamoyl)pyrimidin-2-yl)piperazin-1-yl)sulfonyl)benzenesulfonyl fluoride

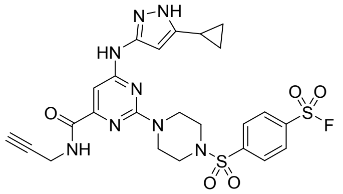

5a

Following the general procedure A, treatment of **S5** (16.0 mg, 0.033 mmol), with 4-(chlorosulfonyl)benzenesulfonyl fluoride (8.6 mg, 0.033 mmol) and triethylamine (4.6 µL, 0.033 mmol) afforded 4-((4-(4-((5-cyclopropyl-1H-pyrazol-3-yl)amino)-6-(prop-2-yn-1-ylcarbamoyl)pyrimidin-2-yl)piperazin-1-yl)sulfonyl)benzenesulfonyl fluoride **5a** (10.4 mg, 0.018 mmol, 53% yield) as a white solid.

δ_H ppm (400 MHz, DMSO-d$_6$)	12.03 (1 H, br s) 9.76 (1 H, br s) 8.89 (1 H, t, *J*=5.9 Hz) 8.40–8.34 (2 H, m) 8.16–8.11 (2 H, m) 6.85 (1 H, br s) 6.08 (1 H, br s) 4.03–3.98 (2 H, m) 3.97–3.90 (4 H, m) 3.13–3.07 (5 H, m) 1.94–1.85 (1 H, m) 0.97–0.90 (2 H, m) 0.73–0.65 (2 H, m)
δ_F ppm (471 MHz, DMSO-d$_6$)	63.82 (1 F, s)
δ_C ppm (101 MHz, DMSO-d$_6$)	163.9, 160.7, 142.8, 130.2, 129.7, 81.6, 73.1, 46.1, 43.2, 28.6, 8.3, 7.3
LC-MS	t_r = 1.12 min, 100% by UV, $[M+H]^+$ found: 589
HRMS	($C_{24}H_{25}FN_8O_5S_2$) [M+H]+ requires 589.1373, found [M+H]+ = 589.1373.

3-((4-(4-((5-Cyclopropyl-1H-pyrazol-3-yl)amino)-6-(prop-2-yn-1-ylcarbamoyl)pyrimidin-2-yl)piperazin-1-yl)sulfonyl)benzenesulfonyl fluoride

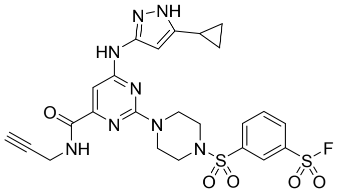

5b

Following the general procedure A, treatment of **S5** (15.0 mg, 0.031 mmol), with 3-(chlorosulfonyl)benzenesulfonyl fluoride (8.1 mg, 0.031 mmol) and triethylamine (4.4 µL, 0.031 mmol) afforded 3-((4-(4-((5-cyclopropyl-1H-pyrazol-3-yl)amino)-6-(prop-2-yn-1-ylcarbamoyl)pyrimidin-2-yl)piperazin-1-yl)sulfonyl)benzenesulfonyl fluoride **5b** (10.7 mg, 0.018 mmol, 58% yield) as a white solid.

δ_H ppm (400 MHz, DMSO-d$_6$)	12.01 (1 H, br s) 9.85–9.65 (1 H, m) 8.90 (1 H, t, *J*=6.2 Hz) 8.51–8.45 (1 H, m) 8.36–8.31 (1 H, m) 8.28 (1 H, t, *J*=1.7 Hz) 8.09–8.03 (1 H, m) 6.84 (1 H, br s) 6.08 (1 H, br s) 4.02–3.98 (2 H, m) 3.97–3.83 (4 H, m) 3.14–3.09 (4 H, m) 3.09–3.06 (1 H, m) 1.94–1.85 (1 H, m) 0.97–0.90 (2 H, m) 0.72–0.65 (2 H, m)
δ_F ppm (471 MHz, DMSO-d$_6$)	64.22 (1 F, s)
δ_C ppm (101 MHz, DMSO-d$_6$)	164.0, 137.9, 135.5, 133.6, 133.4, 132.8, 127.2, 81.7, 73.1, 46.1, 43.2, 28.6, 8.3
LC-MS	t_r = 1.12 min, 96% by UV, $[M+H]^+$ found: 589
HRMS	($C_{24}H_{25}FN_8O_5S_2$) [M+H]+ requires 589.1373, found [M+H]+ = 589.1439.

4-(4-(4-((5-Cyclopropyl-1H-pyrazol-3-yl)amino)-6-(prop-2-yn-1-ylcarbamoyl)pyrimidin-2-yl)piperazine-1-carbonyl)benzenesulfonylfluoride hexafluorophosphoric acid

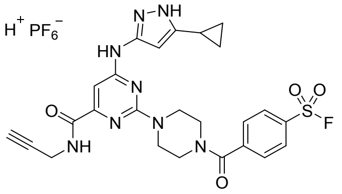

5c

Following the general procedure B, treatment of **S5** (15.0 mg, 0.031 mmol) with 4-(fluorosulfonyl)benzoic acid (6.4 mg, 0.031 mmol), HATU (20.2 mg, 0.053 mmol) and DIPEA (8.2 µL, 0.047 mmol) afforded 4-(4-(4-((5-cyclopropyl-1H-pyrazol-3-yl)amino)-6-(prop-2-yn-1-ylcarbamoyl)pyrimidin-2-yl)piperazine-1-carbonyl)benzenesulfonyl fluoride hexafluorophosphoric acid **5c** (7.0 mg, 10.01 µmol, 32% yield) as a white solid.

δ_H ppm (400 MHz, MeOH-d_4)	8.37–8.33 (1 H, m) 8.24–8.21 (2 H, m) 7.85 (2 H, d, *J*=7.9 Hz) 6.87 (1 H, s) 4.17 (2 H, d, *J*=2.4 Hz) 4.09–4.06 (2 H, m) 3.99–3.89 (4 H, m) 3.58–3.52 (2 H, m) 2.68–2.67 (1 H, m) 2.61 (1 H, t, *J*=2.4 Hz) 2.08–2.01 (1 H, m) 1.23–1.11 (2 H, m) 0.98–0.85 (2 H, m)
δ_F ppm (471 MHz, MeOH-d_4)	63.78 (1 F, s) -74.4 (6 F, d)
δ_C ppm (101 MHz, MeOH-d_4)	168.7, 165.9, 159.3, 156.6, 142.8, 130.7, 128.7, 128.4, 94.6, 78.9, 70.7, 43.5, 39.0, 28.2, 8.1, 6.5
LC-MS	t_r = 1.03 min, 97% by UV, $[M+H]^+$ found: 553
HRMS	($C_{25}H_{25}FN_8O_4S$) [M+H]+ requires 553.1703, found [M+H]+ = 553.1798.

3-(4-(4-((5-Cyclopropyl-1H-pyrazol-3-yl)amino)-6-(prop-2-yn-1-ylcarbamoyl)pyrimidin-2-yl)piperazine-1-carbonyl)benzenesulfonyl fluoride hexafluorophosphoric acid

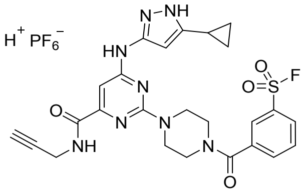

5d

Following the general procedure B, treatment of **S5** (5.8 mg, 0.028 mmol) with 4-(fluorosulfonyl)benzoic acid (15 mg, 0.028 mmol), HATU (18.3 mg, 0.048 mmol) and DIPEA (7.4 µL, 0.042 mmol) afforded 3-(4-(4-((5-cyclopropyl-1H-pyrazol-3-yl)amino)-6-(prop-2-yn-1-ylcarbamoyl)pyrimidin-2-yl)piperazine-1-carbonyl)benzenesulfonyl fluoride hexafluorophosphoric acid **5d** (8.4 mg, 0.012 mmol, 43% yield) as a yellow solid.

δ_H ppm (500 MHz, MeOH-d_4)	8.62 (1 H, t, *J*=2.0 Hz) 8.21 - 8.26 (2 H, m) 8.00 - 8.05 (1 H, m) 7.85 - 7.93 (2 H, m) 6.88 (1 H, s) 4.17 (2 H, d, *J*=2.46 Hz) 4.03 - 4.12 (2 H, m) 3.87 - 4.02 (4 H, m) 3.54 - 3.67 (2 H, m) 2.61 (1 H, t, *J*=2.46 Hz) 2.02 - 2.10 (1 H, m) 1.16 - 1.23 (2 H, m) 0.89 - 0.94 (2 H, m)
δ_F ppm (471 MHz, MeOH-d_4)	63.90 (1 F, s) -74.82 (6 F, d)
δ_C ppm (101 MHz, MeOH-d_4)	168.3, 165.5, 159.9, 145.0, 137.4, 136.3, 134.2, 131.8, 130.5, 126.9, 94.5, 78.9, 70.7, 39.0, 28.2, 8.0, 6.5
LC-MS	t_r = 1.01 min, 94% by UV, $[M+H]^+$ found: 553
HRMS	($C_{25}H_{25}FN_8O_4S$) [M+H]+ requires 553.1703, found [M+H]+ = 553.1765.

4-(2-(4-(4-((5-Cyclopropyl-1H-pyrazol-3-yl)amino)-6-(prop-2-yn-1-ylcarbamoyl)pyrimidin-2-yl)piperazin-1-yl)-2-oxoethyl)benzenesulfonyl fluoride hexafluorophosphoric acid

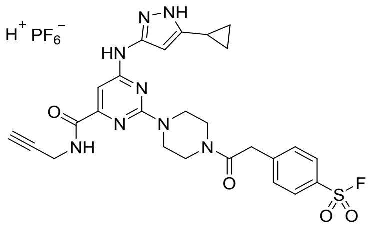

5e

Following the general procedure B, treatment of amine **S5** (15.0 mg, 0.031 mmol) with 2-(4-(fluorosulfonyl)phenyl)acetic acid (8.9 mg, 0.041 mmol), HATU (20.2 mg, 0.053 mmol) and DIPEA (8.2 µL, 0.047 mmol) afforded 4-(2-(4-(4-((5-cyclopropyl-1H-pyrazol-3-yl)amino)-6-(prop-2-yn-1-ylcarbamoyl)pyrimidin-2-yl)piperazin-1-yl)-2-oxoethyl)benzenesulfonyl fluoride hexafluorophosphoric acid **5e** (7.7 mg, 10.8 µmol, 35% yield) as a white solid.

δ_H ppm (400 MHz, MeOH-d$_4$)	8.06–8.02 (2 H, m) 7.68–7.63 (2 H, m) 6.86 (1 H, s) 4.17 (2 H, d, *J*=2.4 Hz) 4.06 (2 H, s) 3.97–3.90 (4 H, m) 3.78–3.73 (4 H, m) 2.67 (2 H, s) 2.61 (1 H, t, *J*=2.4 Hz) 2.08–1.98 (1 H, m) 1.20–1.13 (2 H, m) 0.93–0.86 (2 H, m)
δ_F ppm (471 MHz, MeOH-d$_4$)	62.61 (1 F, s), -76.46 (6 F, d)
δ_C ppm (101 MHz, MeOH-d$_4$)	169.5, 130.7, 128.2, 70.7, 45.2, 43.8, 43.7, 41.3, 39.4, 28.2, 7.9, 6.5. Peaks missing due to weak spectrum.
LC-MS	t_r = 1.04 min, 100% by UV, $[M+H]^+$ found: 567
HRMS	($C_{26}H_{27}FN_8O_4S$) [M+H]+ requires 567.186, found [M+H]+ = 567.1916.

3-(4-(4-((5-Cyclopropyl-1H-pyrazol-3-yl)amino)-6-(prop-2-yn-1-ylcarbamoyl)pyrimidin-2-yl)piperazine-1-carbonyl)-4-methoxybenzenesulfonyl fluoride hexafluorophosphoric acid

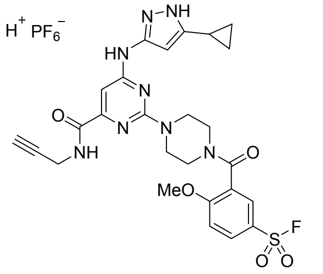

5f

Following the general procedure B, treatment of **S5** (15.0 mg, 0.032 mmol) with 5-(fluorosulfonyl)-2-methoxybenzoic acid (7.5 mg, 0.032 mmol), HATU (20.7 mg, 0.054 mmol) and DIPEA (8.4 µL, 0.048 mmol) afforded 3-(4-(4-((5-cyclopropyl-1H-pyrazol-3-yl)amino)-6-(prop-2-yn-1-ylcarbamoyl)pyrimidin-2-yl)piperazine-1-carbonyl)-4-methoxybenzenesulfonyl fluoride hexafluorophosphoric acid **5f** (9.1 mg, 0.012 mmol, 39% yield) as a white solid.

δ_H ppm (600 MHz, MeOH-d$_4$)	8.19 (1 H, dd, *J*=9.0, 2.2 Hz) 8.04–8.02 (1 H, d, *J*=2.2 Hz) 7.47 (1 H, d, *J*=9.0 Hz) 6.88 (1 H, s) 4.17 (2 H, d, *J*=2.2 Hz) 4.14–3.98 (5 H, m) 3.98–3.68 (4 H, m) 3.47–3.38 (2 H, m) 2.60 (1 H, t, *J*=2.6 Hz) 2.04–1.98 (1 H, m) 1.15–1.09 (2 H, m) 0.88–0.81 (2 H, m)
δ_F ppm (471 MHz, MeOH-d$_4$)	65.27 (1 F, s) -74.30 (6 F, d)
δ_C ppm (101 MHz, MeOH-d$_4$)	165.9, 163.9, 161.2, 160.6, 159.7, 156.4, 145.6, 132.1, 128.6, 126.7, 112.5, 96.5, 94.3, 79.0, 70.7, 55.9, 46.4, 43.4, 41.6, 39.0, 28.1, 17.4, 7.6, 6.4
LC-MS	t_r = 1.00 min, 98% by UV, $[M+H]^+$ found: 583
HRMS	($C_{26}H_{27}FN_8O_5S$) [M+H]+ requires 583.1809, found [M+H]+ = 583.188.

4-(4-(4-((5-Cyclopropyl-1H-pyrazol-3-yl)amino)-6-(prop-2-yn-1-ylcarbamoyl)pyrimidin-2-yl)piperazine-1-carbonyl)phenylsulfonyl fluoride hexafluorophosphoric acid

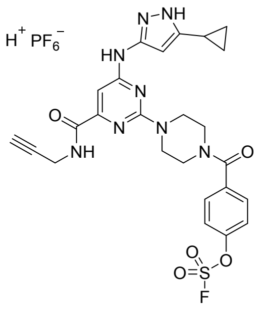

5g

Following the general procedure B, treatment of **S5** (15.0 mg, 0.041 mmol) with 4-((fluorosulfonyl)oxy)benzoic acid (20 mg, 0.091 mmol) HATU (26.5 mg, 0.070 mmol) and DIPEA (10.7 µL, 0.061 mmol) afforded an off-white solid. The solid was dissolved in ethyl acetate (5 mL) and aq. sat. sodium bicarbonate solution (5 mL) and partitioned, then the aqueous layer was re-extracted with ethyl acetate (2 × 10 mL). The combined organic layers were dried (hydrophobic frit), then concentrated in vacuo to afford an off-white solid. The solid was purified by MDAP, then reverse phase chromatography to afford 4-(4-(4-((5-cyclopropyl-1H-pyrazol-3-yl)amino)-6-(prop-2-yn-1-ylcarbamoyl)pyrimidin-2-yl)piperazine-1-carbonyl)phenylsulfonyl fluoride hexafluorophosphoric acid **5g** (5.6 mg, 9.9 µmol, 19% yield) as a white solid.

δ_H ppm (400 MHz, MeOH-d$_4$)	7.73–7.69 (2 H, m) 7.64–7.60 (2 H, m) 6.89 (1 H, s) 4.17–4.13 (2 H, d, J=2.5 Hz) 4.08–3.98 (2 H, m) 3.97–3.81 (4 H, m) 3.61–3.49 (2 H, m) 2.60 (1 H, t, *J*=2.5 Hz) 1.99–1.91 (1 H, m) 1.05–0.99 (2 H, m) 0.79–0.74 (2 H, m)
δ_F ppm (471 MHz, MeOH-d$_4$)	36.22 (1 F, s) -77.11 (6 F, s)
δ_C ppm (101 MHz, MeOH-d$_4$)	129.4, 121.3, 70.5, 28.1, 7.0. Peaks missing due to weak spectrum.
LC-MS	t_r = 1.05 min, 100% by UV, $[M+H]^+$ found: 569
HRMS	($C_{25}H_{25}FN_8O_5S$) [M+H]+ requires 569.1653, found [M+H]+ = 569.1729.

5-(4-(4-((5-Cyclopropyl-1H-pyrazol-3-yl)amino)-6-(prop-2-yn-1-ylcarbamoyl)pyrimidin-2-yl)piperazine-1-carbonyl)-1H-pyrrole-3-sulfonyl fluoride hexafluorophosphoric acid

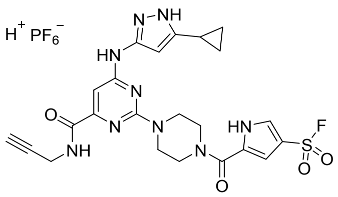

5h

Following the general procedure B, treatment of **S5** (15.0 mg, 0.031 mmol) with 4-(fluorosulfonyl)-1H-pyrrole-2-carboxylic acid (7.8 mg, 0.041 mmol), HATU (20.2 mg, 0.053 mmol) and DIPEA (10.9 µL, 0.062 mmol) afforded 5-(4-(4-((5-cyclopropyl-1H-pyrazol-3-yl)amino)-6-(prop-2-yn-1-ylcarbamoyl)pyrimidin-2-yl)piperazine-1-carbonyl)-1H-pyrrole-3-sulfonyl fluoride hexafluorophosphoric acid **5h** (15.4 mg, 0.022 mmol, 72% yield) as a white solid.

δ_H ppm (600 MHz, MeOH-d$_4$)	7.81–7.79 (1 H, m) 7.11 (1 H, d, *J*=1.5 Hz) 6.88 (1 H, br s) 6.08 (1 H, br s) 4.17 (2 H, d, *J*=2.5 Hz) 4.03–3.98 (4 H, m) 3.95–3.88 (4 H, m) 2.60 (1 H, t, *J*=2.5 Hz) 2.00–1.87 (1 H, m) 1.04–0.98 (2 H, m) 0.79–0.71 (2 H, m)
δ_F ppm (471 MHz, MeOH-d$_4$)	68.06 (1 F, s), -74.50 (6 F, d)
δ_C ppm (151 MHz, MeOH-d$_4$)	161.1, 160.9, 127.2, 126.8, 115.1, 111.1, 79.2, 70.5, 43.5, 39.0, 33.7, 28.1, 15.8, 7.6
LC-MS	t_r = 0.97 min, 98% by UV, $[M+H]^+$ found: 542
HRMS	($C_{23}H_{24}FN_9O_4S$) [M+H]+ requires 542.1656, found [M+H]+ = 542.1719.

3-(4-(4-((5-Cyclopropyl-1H-pyrazol-3-yl)amino)-6-(prop-2-yn-1-ylcarbamoyl)pyrimidin-2-yl)piperazine-1-carbonyl)azetidine-1-sulfonyl fluoride

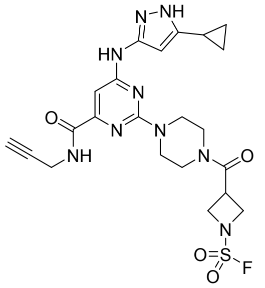

5i

Following the general procedure B, treatment of **S5** (15.0 mg, 0.031 mmol) with 4-(fluorosulfonyl)-1H-pyrrole-2-carboxylic acid (5.7 mg, 0.031 mmol), HATU (20.2 mg, 0.053 mmol) and DIPEA (8.2 µL, 0.047 mmol) afforded 3-(4-(4-((5-cyclopropyl-1H-pyrazol-3-yl)amino)-6-(prop-2-yn-1-ylcarbamoyl)pyrimidin-2-yl)piperazine-1-carbonyl)azetidine-1-sulfonyl fluoride **5i** (7.9 mg, 0.015 mmol, 48% yield) as an off-white solid.

δ_H ppm (600 MHz, MeOH-d$_4$)	6.89 (1 H, br s) 6.13 (1 H, br s) 4.48–4.34 (4 H, m) 4.16 (2 H, d, *J*=2.3 Hz) 4.08–3.96 (1 H, m) 3.96–3.89 (4 H, m) 3.77–3.68 (2 H, m) 3.52–3.44 (2 H, m) 2.61 (1 H, t, *J*=2.5 Hz) 2.00–1.82 (1 H, m) 1.06–0.92 (2 H, m) 0.82–0.69 (2 H, m)
δ_F ppm (471 MHz, MeOH-d$_4$)	26.54 (1 F, s)
δ_C ppm (101 MHz, MeOH-d$_4$)	169.1, 168.9, 164.1, 161.1, 93.3, 79.2, 70.6, 56.1, 54.4, 44.8, 43.2, 41.8, 28.1, 15.7, 8.5, 6.8
LC-MS	t_r = 0.98 min, 100% by UV, $[M+H]^+$ found: 532
HRMS	($C_{22}H_{26}FN_9O_4S$) [M+H]+ requires 532.1813, found [M+H]+ = 532.1887.

Additional **XO44** probe was purchased from Sigma Aldrich (PF-6808472).

4. Intrinsic reactivity studies

4.1. Buffer composition

Identity	pH	Concentration / mM	Details
PBS	7	0.1	NaCl (0.137 M), potassium phosphate monobasic (1.47 mM), sodium phosphate dibasic (8.11 mM), KCl (2.69 mM). pH adjusted with NaOH (5 M). Filtered through a 0.22 µm filter.
HEPES	7	0.1	H3375, CAS Number 7365-45-9, Sigma Aldrich. pH adjusted with NaOH (5 M).
PBS	8	0.1	NaCl (1.37 M), potassium phosphate monobasic (14.7 mM), sodium phosphate dibasic (81.1 mM), KCl (26.9 mM). pH adjusted with NaOH (5 M). Filtered through a 0.22 µm filter.
HEPES	8	0.1	H3375, CAS Number 7365-45-9, Sigma Aldrich. pH adjusted with NaOH (5 M).
$NaHCO_3$/ Na_2CO_3	10	0.1	Sodium bicarbonate (0.0461 M), sodium carbonate (0.0539 M).

4.2. Hydrolysis study protocol

To each S^{VI}-F compound **1a**–**i** (60 µL, 10 mM in DMSO) in a glass LC-MS vial was added DMSO (80 µL) and either 1,4-dicyanobenzene solution or methyl *p*-tolyl sulfone solution (as an internal standard, 60 µL, 10 mM in DMSO), then finally, buffer solution (0.1 M, 800 µL). The vial was mixed, then analysed by HPLC (2 µL injection volume) at intervals of approximately 40 min.

Final concentrations: sulfur(VI) fluoride **1a**–**i** (0.6 mM), 1,4-dicyanobenzene (0.6 mM) in buffer incl. 20% DMSO by volume.

Final volume: 1 mL

Raw UV area data and timepoint data was extracted from ChromView, then processed in Excel. The data was normalised to the peak area of internal standard (methyl *p*-tolyl sulfone or 1,4-dicyanobenzene), then normalised to the maximum concentration of starting material. The reaction progress was analysed in GraphPad Prism® (one-phase decay model, constrained plateau value to '0'), assuming pseudo-first order kinetics to calculate rates of hydrolysis (k) and half-lives ($t_{1/2}$ = 0.693/k).[1] Samples were analysed by LC-MS after reaction to confirm the only product as the corresponding sulfonic acid.

4.3. Amino acid reactivity study protocol

Table S7. The composition of the ***N*-Ac-Cys, -Tyr, -His** amino acid stock solutions

Stock solution	Composition	Volume / µL	Final concentration / mM
Amino acid: *N*-Ac-Cys/Tyr/His	50 mM in DMSO	240	2
Internal standard: methyl p-tolyl sulfone/1,4-dicyanobenzene	10 mM in DMSO	360	0.6
DMSO	-	580	-
Buffer (PBS, pH 8.0)	-	4680	-

Table S8. The composition of the ***N*-Ac-Lys** amino acid stock solution

Stock solution	Composition	Volume / µL	Final concentration / mM
Amino acid: *N*-Ac-Lys	50 mM in buffer (PBS, pH 8.0)	240	2
Internal standard: methyl p-tolyl sulfone/1,4-dicyanobenzene	10 mM in DMSO	360	0.6
DMSO	-	820	-
Buffer (PBS, pH 8.0)	-	4440	-

***N*-Ac-Cys, -Tyr, -His, -Lys:**

To sulfur(VI) fluoride **1a**–**h** (20 µL, 10 mM in DMSO) in a glass LC-MS vial was added amino acid stock solution (980 µL, composition as described in Table S7 and S8), then the solution was mixed with a pipette, The vial was analysed by HPLC (2 µL injection volume) at intervals of approximately 40 min. Raw UV area data was extracted from ChromView, then processed in excel. Timepoints were extracted, and the starting material peak data normalised to the internal standard.

Final concentrations: sulfur(VI) fluoride **1a**–**h** (0.2 mM), 1,4-dicyanobenzene (0.6 mM), *N*-Ac amino acid (2 mM). Final solution is buffer incl. 20% DMSO by volume. Final volume: 1.0 mL

Analysis of rate data was undertaken in GraphPad Prism® 5.0.4 software. Normalised starting material UV peak data and timepoints were transferred from excel to GraphPad, then processed using one-phase decay analyses, with plateau constraint set to '0'.

Competing S^{VI}-F hydrolysis was observed in all reactions, therefore the rate constants for the reactions with *N*-Ac-Tyr and *N*-Ac-Lys were determined by subtraction of the measured hydrolysis rate constant (pH 8, PBS as above) from the overall rate constant for S^{VI}-F consumption (assuming pseudo-first order rate conditions).[1] For *N*-Ac-Cys reactions, rate constants were determined directly from the formation of sulfinic acid. For *N*-Ac-His, no product was observed for any S^{VI}-F fragment.

5. LUMO energy calculations

Quantum chemical calculations were performed to estimate LUMO energies for S^{VI}-F fragments **1a–i** in order to assess correlation with measured hydrolysis rate constants. 3D structures of **1a–i** were created using LigPrep and conformer generation was performed with Schrödinger Macromodel. Geometry optimisation of the resulting conformations was carried out using Gaussian16 using a lower-level semiempirical AM1 method and higher-level DFT B3LYP-D3 functional using both 6-31+G** and aug-cc-PVTZ basis set and a polarizable continuum model (PCM) solvation model with water. Geometry optimisation was used to select the most stable conformer for each structure (at each level), then vibrational frequencies were calculated to determine if the conformer was a minimum of the potential energy surface. The LUMO orbital energy was calculated for the lowest energy conformer of each structure. The calculated LUMO energies were then plotted against the experimentally measured aqueous half-lives (PBS buffer, pH 7.0). The calculations showed that the lower-level semiempirical AM1 method determined the lowest lying conformers differently, and the result gave poor correlation with the experimentally measured aqueous half-lives (PBS buffer, pH 7.0) (R^2 = 0.31). Higher-level DFT with both basis sets showed high coefficients of determination, with R^2 = 0.96 and R^2 = 0.97 for 6-31+G** and aug-cc-PVTZ, respectively.

a)

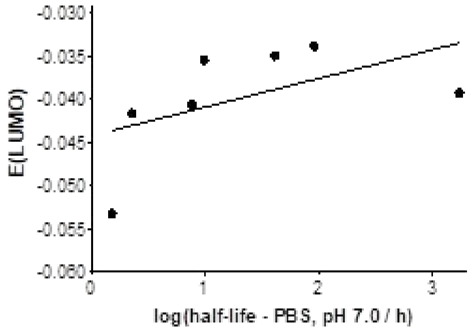

b)

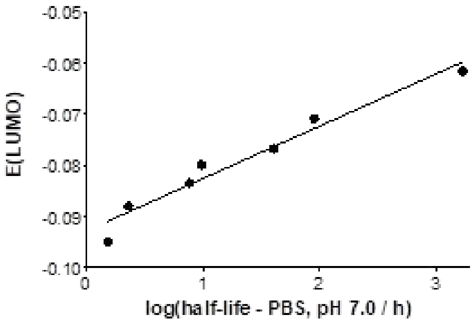

c)

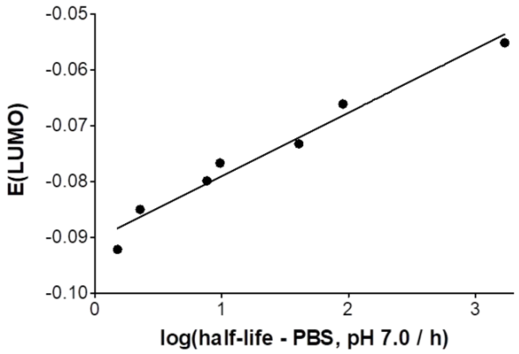

Figure S8. The calculated LUMO energies for the training compounds as a function of measured half-life at pH=7 in PBS buffer.

Predicted half-lives for fragments **1h** and **1i** were determined using the correlation between measured half-lives and LUMO energy values from the B3LYP-D3 with aug-cc-PVTZ basis set, from the following equations where:

x = half-life / h y = LUMO energy

pH 7: $x = 10^{(y+0.09044)/0.01139}$

pH 8: $x = 10^{(y+0.08608)/0.01178}$

6. Protein reactivity studies

6.1. Protein stock solutions

CDK2: recombinant, purchased under exclusive license from Dundee consortium: co-expressed GST-CDK2 with Cyclin A (DU43557), 6H-Flag-Tev-CDK2, supplied as a solution (41 µM, 37100 Mw)

Carbonic anhydrase II human: recombinant, expressed in *E. coli* (Sigma Aldrich, C6624-500UG, Lot: 069M4082L) supplied as a solution (34.19 µM, MW 29246 Da) in 20 mM Tris, pH 7.5, with 150 mM NaCl.

Lysate: HepG2 Princen, PBS buffer, benzonase assisted lysis/no detergent. Cleared by centrifuge (20 min).

6.2. Protein modification study protocols

6.2.1. CAII modification kinetics study

Recombinant CAII protein was thawed then diluted to 1 µM in assay buffer (pH 7.5, 25 mM HEPES, 150 mM NaCl in distilled H_2O). Protein solution (49.5 µL) was pipetted into wells of a Greiner 384-well PP plate (#781280) on ice, then the plate was centrifuged (1000 rpm, 1 min). Solutions of the S^{VI}-F fragments **2a**–**i, 3a,c**–**e, 4a,c**–**e** or DMSO (0.5 µL, concentrations as follows in DMSO) were added to each well at time intervals of 85 seconds to give solutions with final compound concentrations (0 (DMSO control), 5, 10, 20, 50 and 100 µM). The solutions were mixed with a pipette after each addition, and the experiment was performed in duplicate. The plate was centrifuged (1000 rpm, 1 min), then sealed and submitted for analysis across a timecourse by intact-protein LC-MS (2 µL injection volume).

Intact-protein LC-MS analysis was performed, and the resulting modification yields (Equation S1) plotted in GraphPad Prism® 5.0.4 software against the time of sampling. Non-linear regression analyses were conducted using a 'one-phase association' model, with the y=0 value constrained to '0' and plateau value constrained to '100'. The observed rate constants (k_{obs}, value 'K' in GraphPad) were extracted for each compound at each concentration, then replotted against the compound concentration. Further non-linear regression analyses were conducted using a 'Michaelis-Menten' model, from which k_{inact} values ('Vmax' in GraphPad) and K_I values ('Km') were extracted.

6.3. CDK2 modification study

Recombinant CDK2 protein was diluted to 1 µM in assay buffer (pH 7.5, HEPES (25 mM), NaCl (150 mM) in distilled H_2O). Protein solution (49 µL) was pipetted into wells of a Greiner 384-well PP plate (#781280) on ice, then the plate was centrifuged (1000 rpm, 1 min). Solutions of S^{VI}-F probes **5a**–**i** (1 µL, 0.5 mM in DMSO) and a DMSO control (1 µL) were added to each well at time intervals of 85 seconds and mixed with a pipette. This experiment was performed in duplicate. The plate was centrifuged (1000 rpm, 1 min), then sealed and submitted for analysis across a timecourse by intact protein LC-MS.

Final: CDK2 (1 µM), S^{VI}-F probe (10 µM), 50 µL volume per well.

LC-MS analysis was performed (Section 2.9), and the resulting crosslinking yields plotted in GraphPad Prism® 5.0.4 software against the time of sampling. Non-linear regression analysis was conducted using a 'one-phase association' model, with the y=0 value constrained to '0'.

7. Gel electrophoresis with S^{VI}-F probe analogues

Lysis buffer: $MgCl_2$ (1.5 mM), glycerol (5%), NaCl (175 mM) and protease tablet (1X) in PBS buffer (0.1 M).

THPTA click mix: $CuSO_4.5H_2O$ (0.6 mM), Cy5-5-N_3 (0.15 mM), ascorbic acid (1 mM), THPTA (0.6 mM), aminoguanidine (1 mM) in H_2O.

A stock solution of cleared lysate (9.49 mg mL^{-1}) was diluted to 1 mg mL^{-1} using the prepared lysis buffer. To each sample of lysate-buffer solution (25 µL) was added probe (0.5 mM in DMSO, 0.5 µL) or DMSO (0.5 µL). The aliquots were incubated on ice for 2.5 hr. To 15 µL of each solution was added Cy5.5-N_3 (0.125 mM, 2 µL), followed by THPTA click mix (1 µL). To 450 µL of Invitrogen™ Gel Loading Buffer II was added DTT (50 µL, 1 mM). The samples were left on ice for 1 hour, before DTT-loading buffer solution (5 µL) was added. A NuPAGE 12% Bis-Tris gel was prepared in 1X NuPAGE MES SDS running buffer and SeeBlue™ Plus2 Pre-stained Protein Standard (10 µL) added in the first column. Each probe sample (8 µL) was added in separate lanes of the gel. The gel was run for 45 min at constant voltage (200 V, 120 mA, 25.0 W) and analysed on a LI-COR gel reader using Image Studio™ Lite.

8. Chemoproteomic studies with S^{VI}-F probe analogues

8.1. Acetylation of NeutrAvidin agarose resin

NeutrAvidin agarose resin (20 mL, Thermo Fisher Scientific 29204) was centrifuged (2000 rcf, 2 min) and supernatant was removed. The beads were washed three times with PBS buffer, before PBS (9 mL) and Sulfo-NHS-acetate (200 mM freshly made in anhydrous DMSO, 965 µL, Thermo Fisher Scientific, 26777) were added. The beads were incubated for 30 min at rt on a falcon tube roller, then centrifuged (2000 rcf, 2 min). Supernatant was removed and the incubation step with freshly made NHS-acetate was repeated. The reaction was quenched by adding Tris (1 M, 2 mL, pH 7.5). The beads were washed once with PBS and twice with 20% EtOH. EtOH (20%, 10 mL) was added and the beads were stored at 4 °C until required.

8.2. Chemoproteomics workflow

Jurkat cells (2 × 10^6 cells mL^{-1} in serum free RPMI media) were treated in triplicate for 1 h with probes **5a–i** and **XO44** (2 µM final concentration) or DMSO vehicle at 37 °C. Treated cells were pelleted and washed with PBS. Cells were lysed in lysis buffer (SDS 0.1%, 1% IGEPAL, 0.5% Na-deoxycholate, 150 mM NaCl, 50 mM HEPES pH 8.0 and 1× EDTA-free protease inhibitor cocktail) by sonicating for 5 × 1 s. The protein concentration of each lysate was determined using a BCA assay (Thermo Fisher Scientific, 23227).

376 µL of each lysate (concentrations adjusted to 2.3 µg µL^{-1}) was treated with premixed click mixture (24 µL, final concentrations of 100 µM biotin-PEG3-azide, 1 mM $CuSO_4$, 1 mM TCEP and 100 µM BTTAA) for 1 h. The click reaction was quenched by adding of EDTA (8 µL, 500 mM, 10 mM final concentration).

Proteins were precipitated using ice-cold acetone and the resulting pellets washed twice with ice-cold 80% acetone. The air-dried pellets were dissolved in SDS (400 µL, 0.2%) in HEPES (50 mM, pH 8.0) by vortexing and sonicating.

Samples were incubated with acetylated NeutrAvidin agarose resin (1:10 ratio of bead suspension:protein, 85 µL, pre-washed three times with 0.2% SDS in 50 mM HEPES pH 8.0) on a combinatorial microlute plate for 2 h. The plate was centrifuged (700 g, 1 min). The beads were washed three times each with lysis buffer, urea (4 M in 50 mM HEPES pH 8.0) and HEPES (50 mM pH 8.0). The proteins were digested on-bead overnight at 37 °C with LysC (60 µL, 0.004 µg µL^{-1} in 50 mM HEPES pH 8.0). The supernatants were collected and trypsin (40 µL, 0.006 µg µL^{-1} in 50 mM HEPES pH 8.0) was added to each sample. The samples were incubated for 4 h at 37 °C, then formic acid (1 µL) added.

The peptide samples were cleaned-up using C18 96-well plate (BioPureSPE Macro 96-Well, 100mg PROTO C18). The wells were conditioned with acetonitrile (400 µL), centrifuged (50 g, 1 min), equilibrated twice with TFA (0.1% in water, 300 µL), and centrifuged again (150 g, 1 min). TFA (0.1% in water, 100 µL) was added to the digested samples, then the samples were loaded on the plate and centrifuged (150 g, 1 min). The samples were washed twice with TFA (0.1% in water, 200 µL), and centrifuged twice (150 g, 1 min) then (200 g, 1 min). The peptides were eluted twice with TFA (0.1% in 50% acetonitrile, 150 µL) into a collection plate and centrifuged (200 g, 1 min). The plate was frozen, and the samples were dried in a Labconco CentriVap Benchtop Vacuum Concentrator at 35 °C.

8.3. LC-MS/MS analysis

Peptides were redissolved in formic acid (0.1% in water), then 40% of each digested sample and iRT standards (Biognosys AG) were loaded onto Evotips (as prepared according to manufacturer's instructions), followed by loading onto the Evosep One system in front of the Orbitrap Fusion Lumos (Thermo). The Evosep One was fitted with a 15 cm column and the predefined method for an 88 minute was employed. Data for all samples was acquired in Data Independent Acquisition mode (DIA). DIA Lumos settings were as follows: Transfer capillary set to 300 °C and 2.2 kV applied to the nanospray needle (Evosep). MS1 data acquired in the Orbitrap with a resolution of 120 k, max injection time of 20 ms, AGC target of 10^6, in positive ion mode, in profile mode, over the mass range 393–907 *m/z*. DIA segments over this mass range (20 *m/z* wide/1 Da overlap/27 in total) were acquired in the Orbitrap following fragmentation in the HCD cell (32%), with 30k resolution over the mass range 200–2000 *m/z*, max injection time of 54 ms (dynamic), and AGC target of 10^6.

The data were searched using Pulsar search engine in Spectronaut (v.14, Biognosys AG). A spectral library was first generated by searching the data against the Uniprot Proteome Database for "*Homo Sapiens (uniprot-proteome_ UP000005640.fasta), avidin.fasta"* file and a database of common contaminants. BGS factory settings (default) were used, except no fixed modifications were selected. The library contained 50817 precursors that correspond to 38612 peptides from 4843 protein groups.

The data was searched against the generated library using BGS factory settings (default). Run-wise imputation (Q-value percentile = 30%) was applied to dataset. A two sample t-test was carried out in Spectronaut™ software to assess differential abundances (compound vs. DMSO).

8.4. Data analysis

The data was exported into excel and annotated using several databases:

- Kinase annotation – kinome.org
- Target development level – TCRD database
- GO annotation – geneontology.org via spectronaut
- Proteomics publication data – see references in main publication text

Filters were applied to the dataset:

- q ≤ 0.05, avg log2-fold change ≥ 0.58
- No. unique total peptides ≥ 2

Data visualisation of the resulting dataset was carried out in excel, using pivot tables to produce heatmaps (Figure 6C, E, F). The kinome tree was produced using http://www.kinhub.org/kinmap/. Figure 6G was produced using GraphPad Prism®.

The mass spectrometry proteomics data have been deposited to the ProteomeXchange Consortium via the PRIDE [1] partner repository with the dataset identifier PXD037048

9. ^{1}H NMR spectra

1a

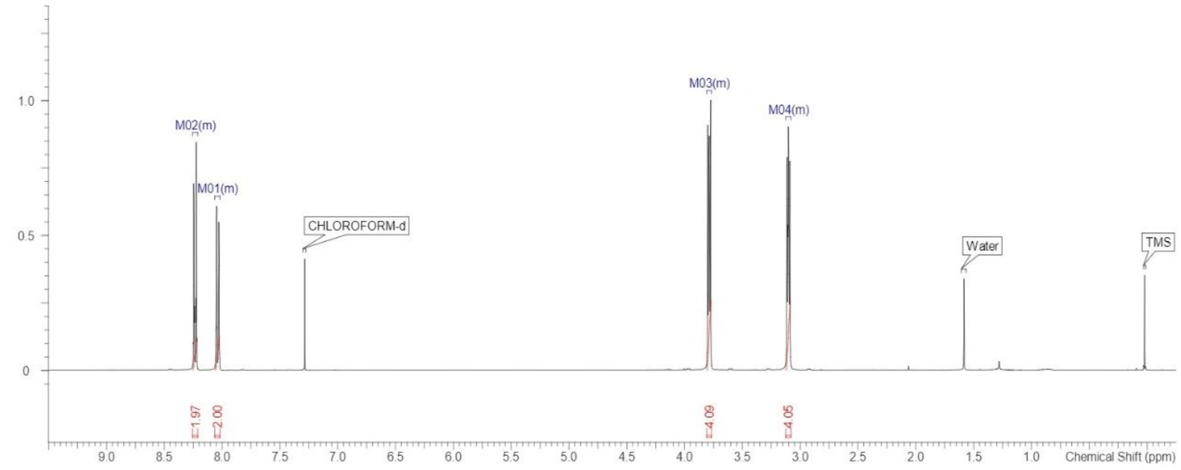

1b

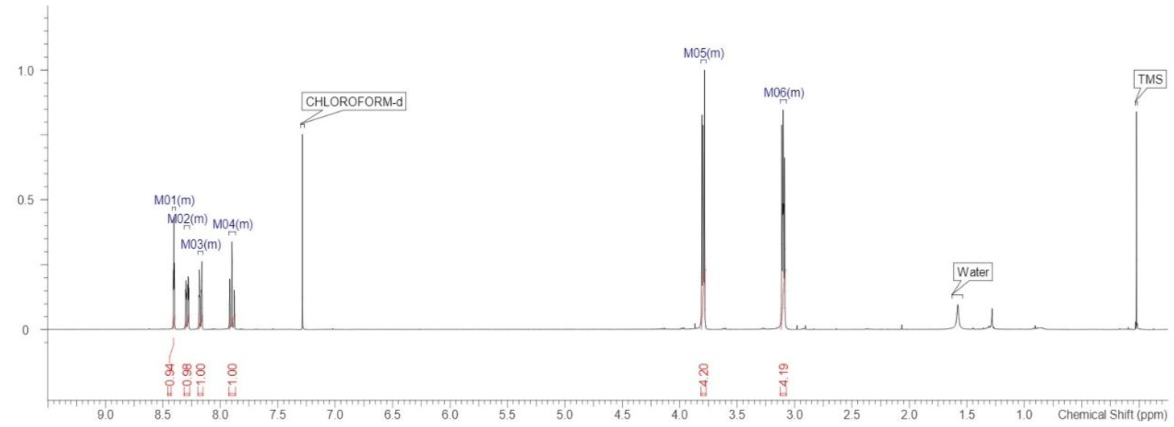

1c

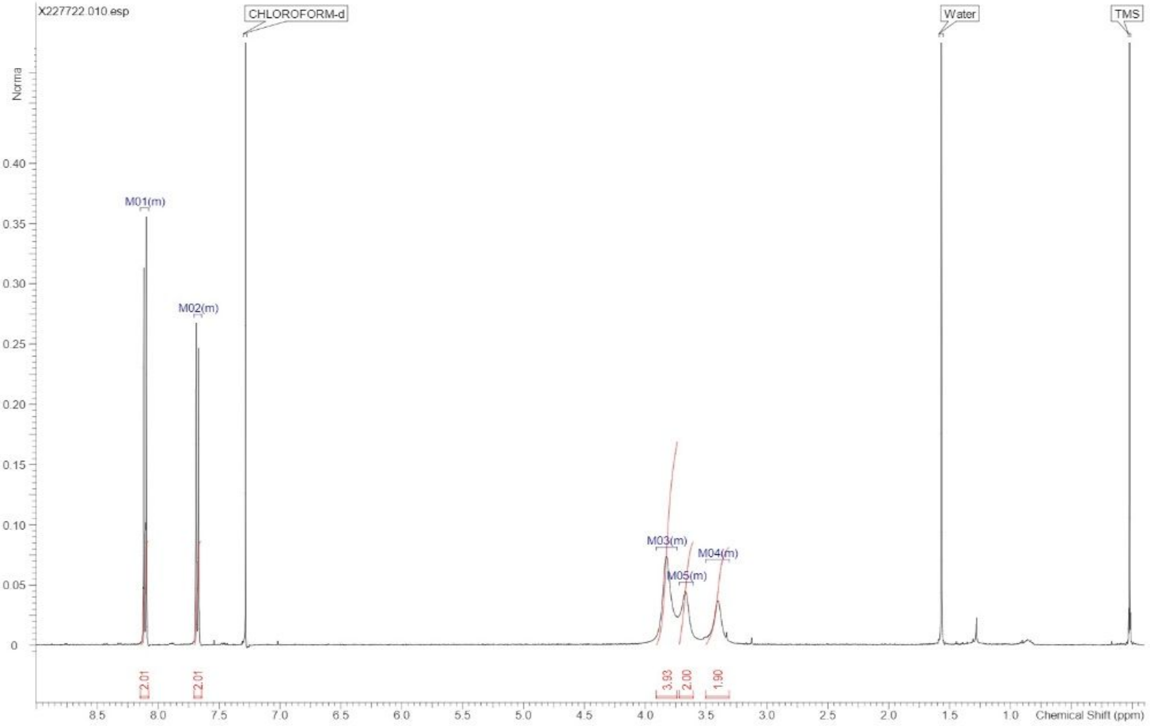

1d

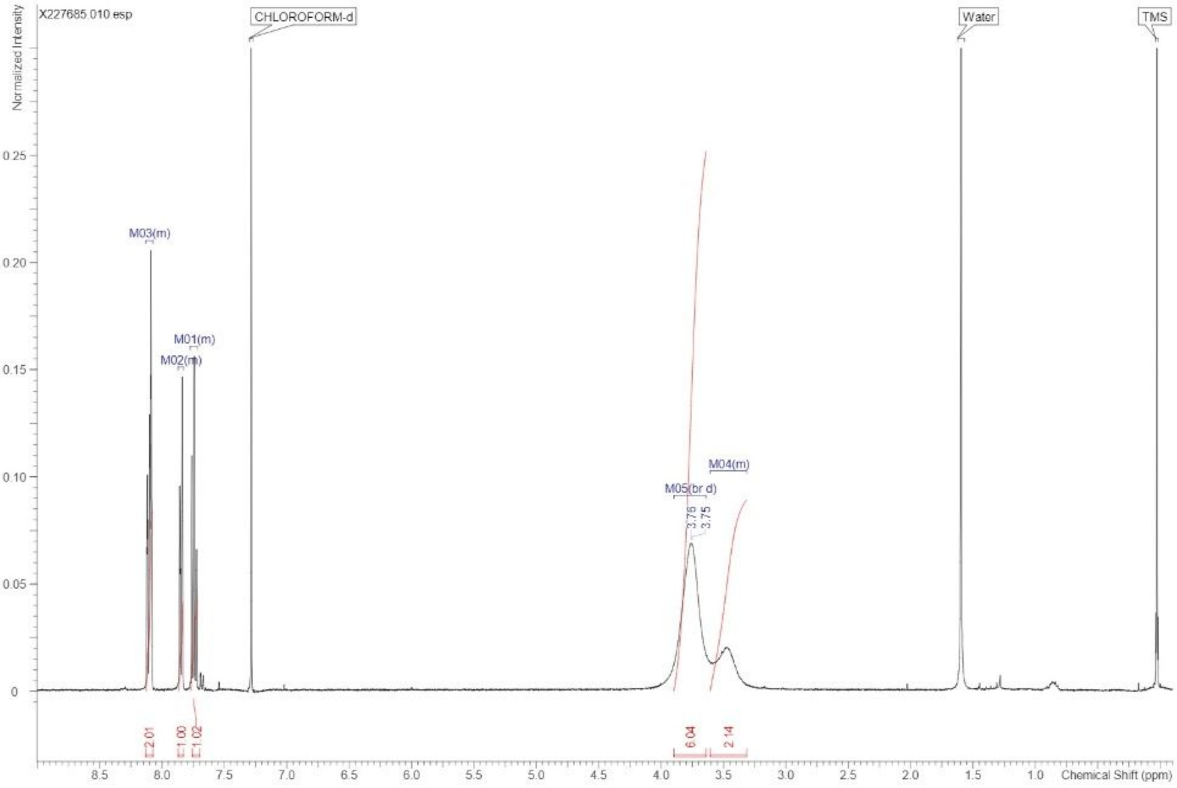
X227685.010.esp
Normalized Intensity
CHLOROFORM-d
Water
TMS
M03(m)
M01(m)
M02(m)
M04(m)
M05(br d)
3.76
3.75
2.01
1.00
1.02
6.04
2.14
Chemical Shift (ppm)

1e

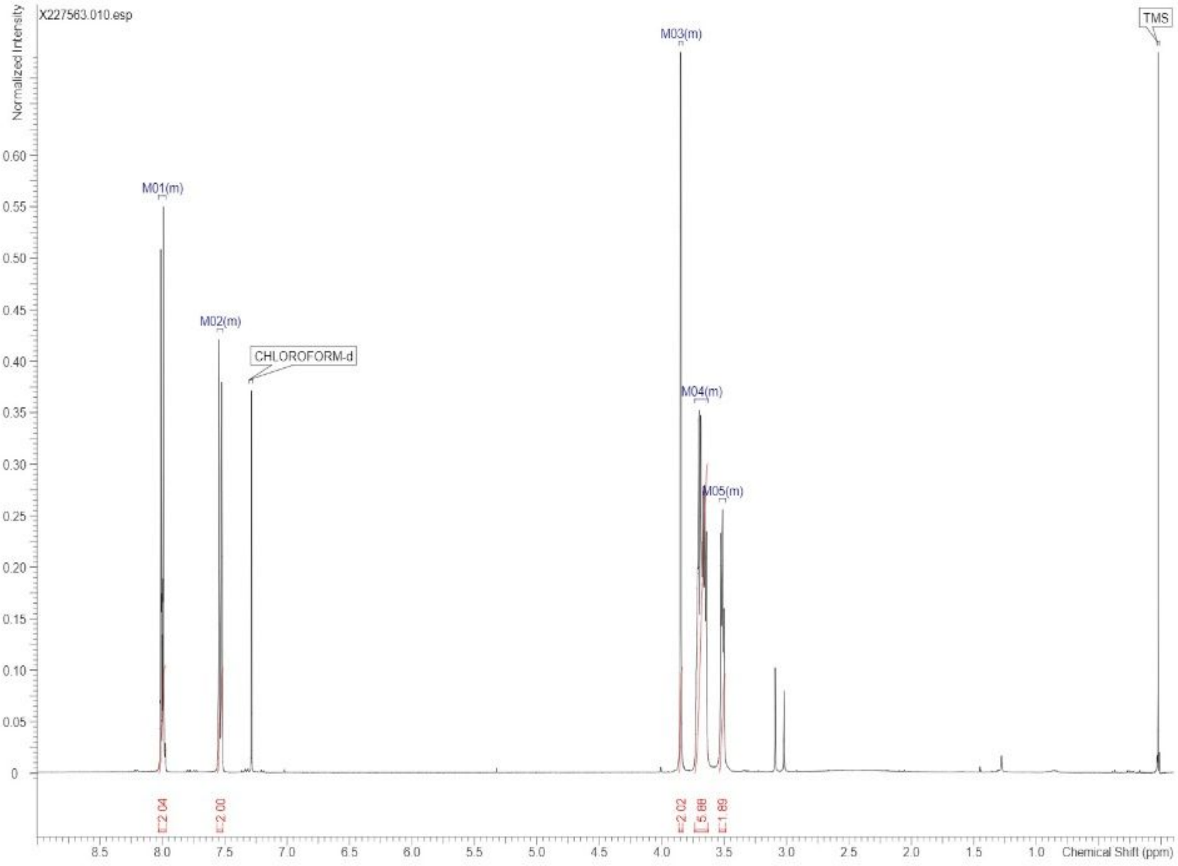
X227563.010.esp
Normalized Intensity
TMS
M03(m)
M01(m)
M02(m)
CHLOROFORM-d
M04(m)
M05(m)
2.04
2.00
2.02
5.88
1.89
Chemical Shift (ppm)

1f

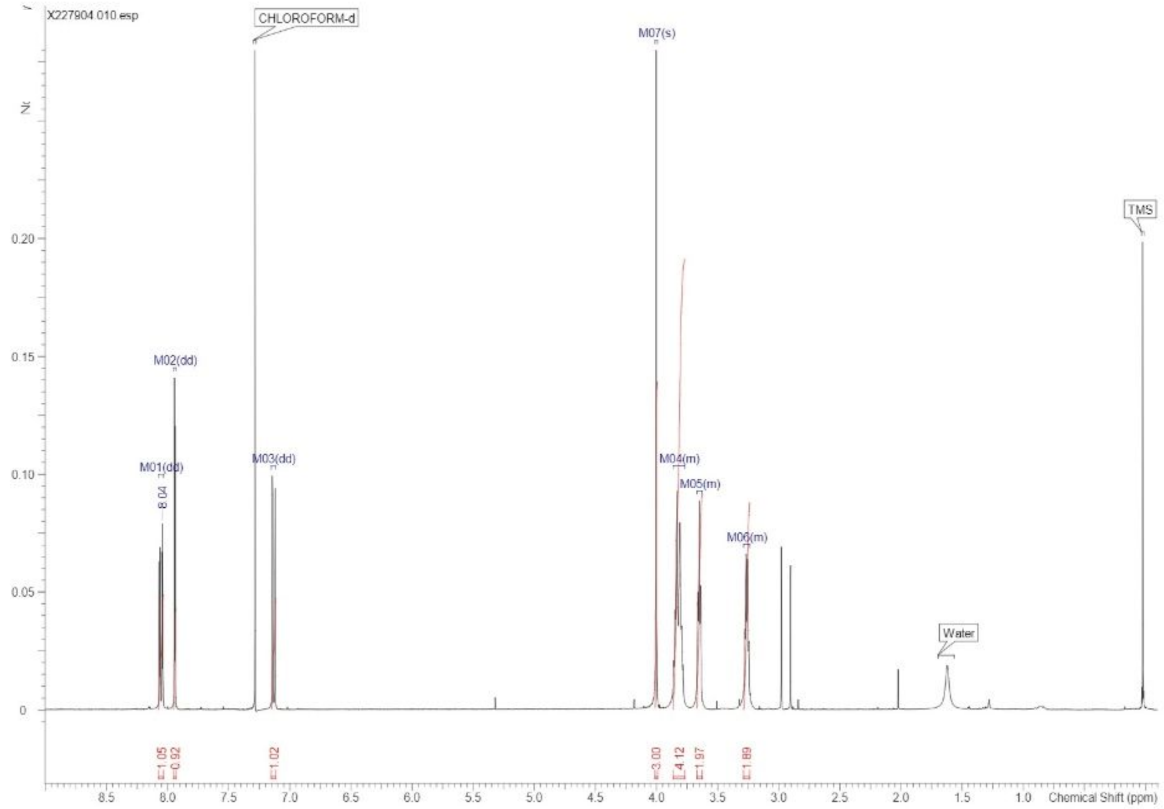
X227904.010.esp
CHLOROFORM-d
M07(s)
TMS
M02(dd)
M01(dd)
8.04
M03(dd)
M04(m)
M05(m)
M06(m)
Water
1.05
0.92
1.02
3.00
4.12
1.97
1.89
Chemical Shift (ppm)

1g

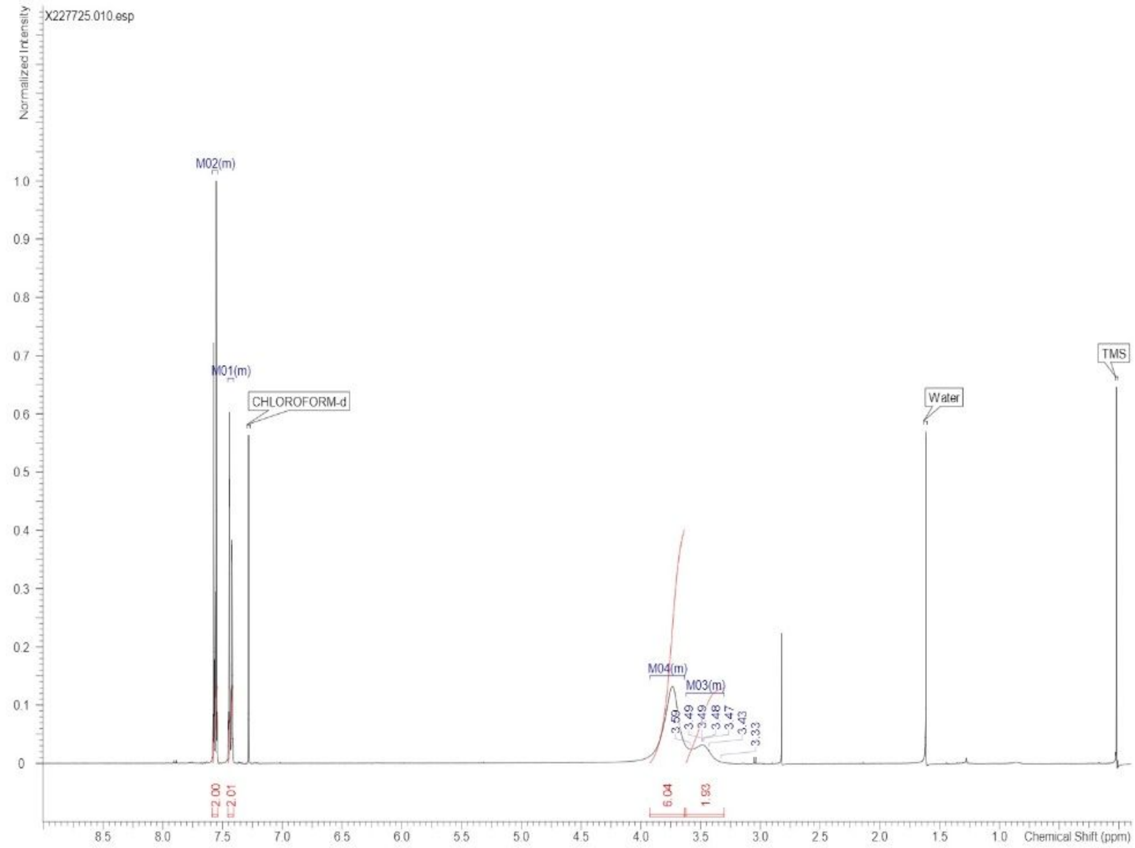
Normalized Intensity
X227725.010.esp
M02(m)
M01(m)
CHLOROFORM-d
Water
TMS
M04(m)
M03(m)
3.59
3.49
3.49
3.48
3.47
3.43
3.33
2.00
2.01
6.04
1.93
Chemical Shift (ppm)

1h

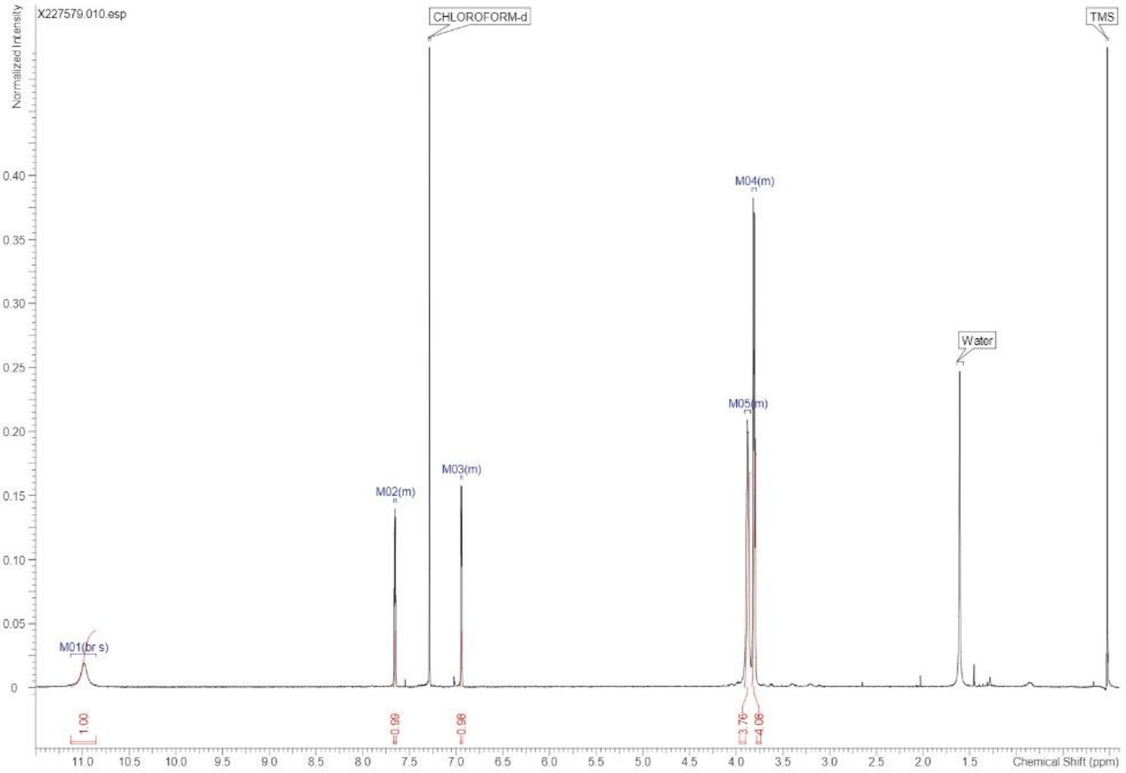
X227579.010.esp
Normalized Intensity
CHLOROFORM-d
TMS
Water
M01(br s)
M02(m)
M03(m)
M04(m)
M05(m)
1.00
0.99
0.98
3.76
4.08
Chemical Shift (ppm)

1i

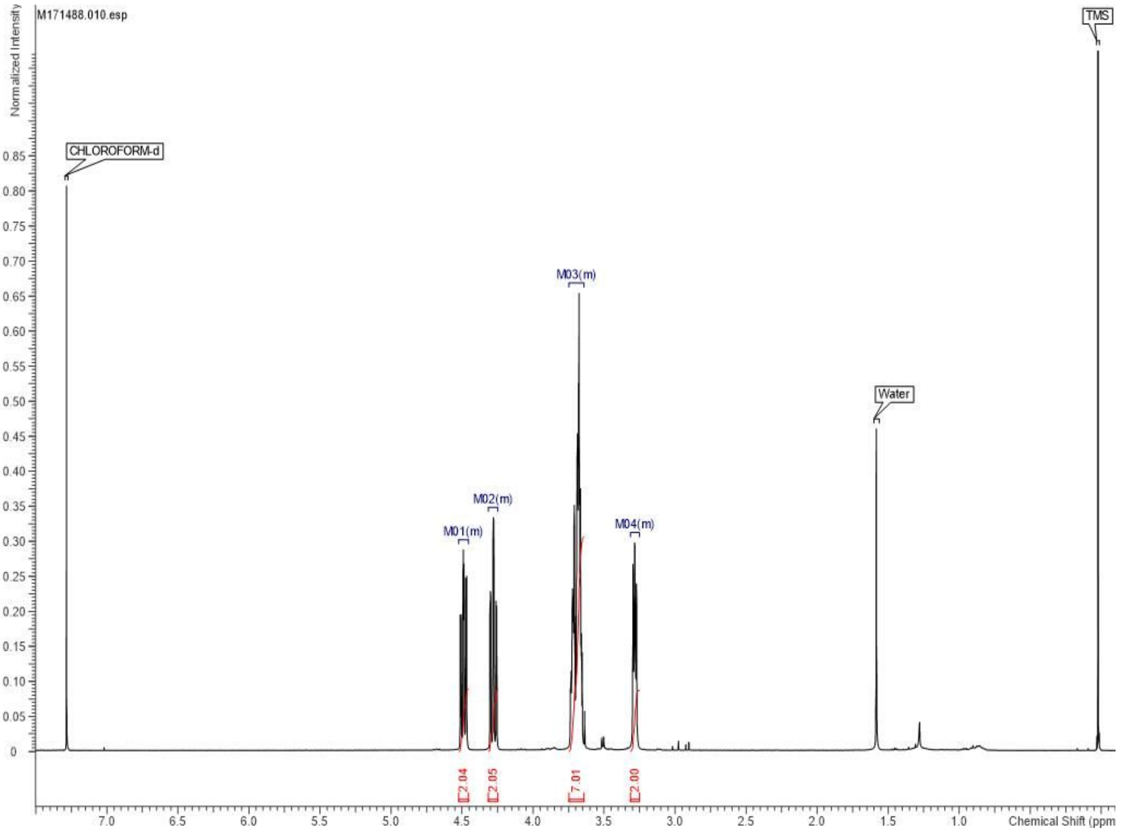
M171488.010.esp
Normalized Intensity
CHLOROFORM-d
TMS
Water
M01(m)
M02(m)
M03(m)
M04(m)
2.04
2.05
7.01
2.00
Chemical Shift (ppm

2a

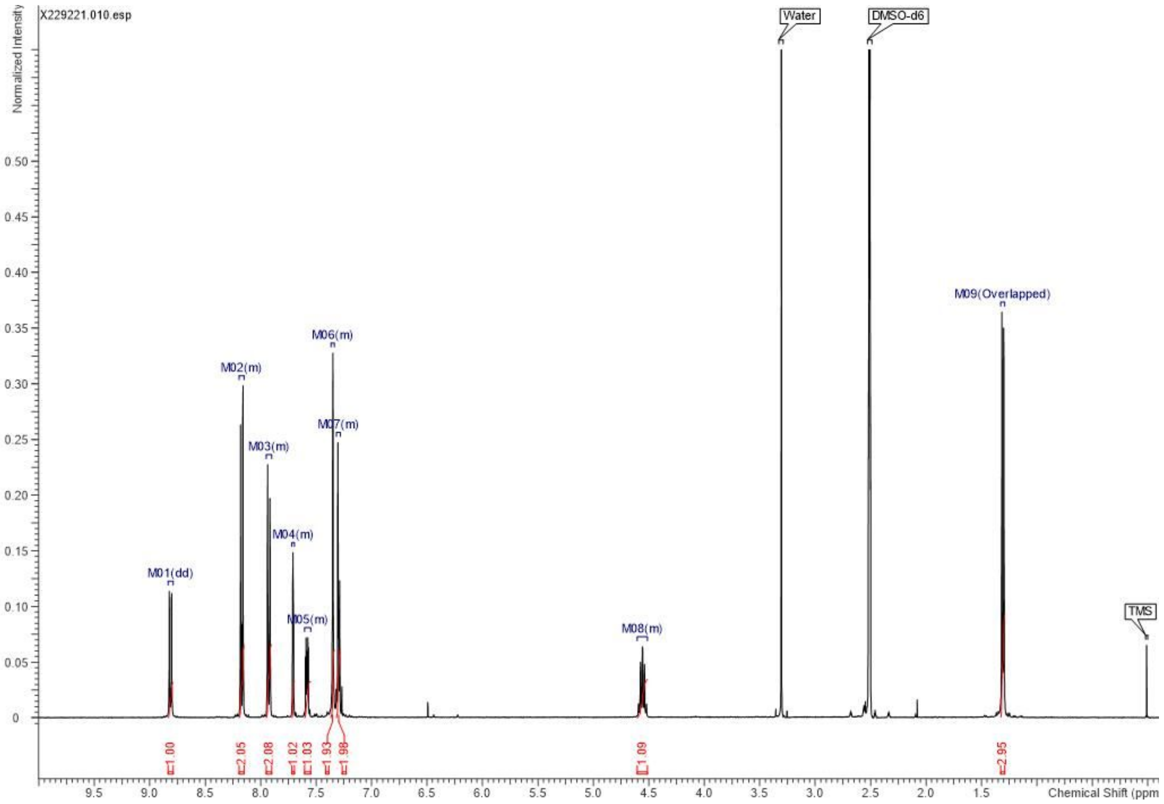
X229221.010.esp
Water
DMSO-d6
M01(dd)
M02(m)
M03(m)
M04(m)
M05(m)
M06(m)
M07(m)
M08(m)
M09(Overlapped)
TMS
Normalized Intensity
Chemical Shift (ppm

2b

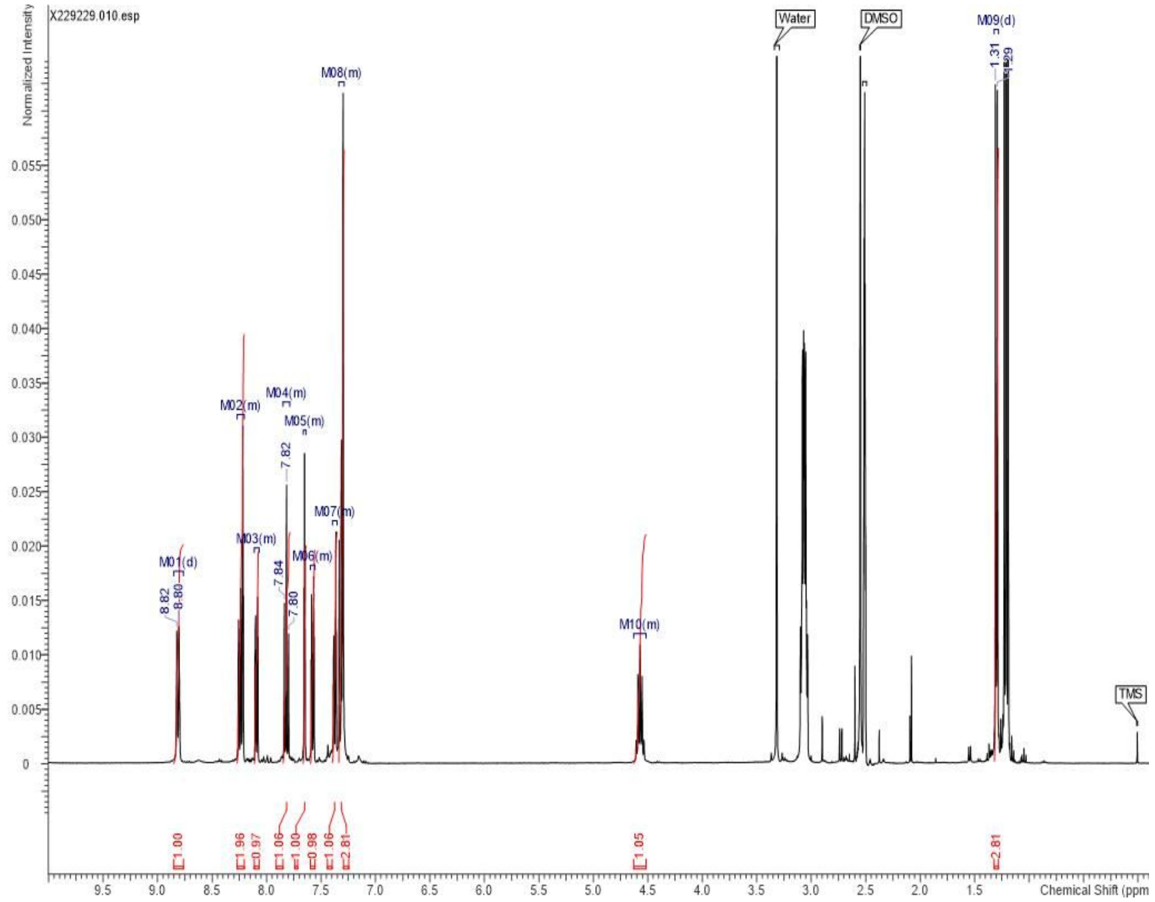
X229229.010.esp
Water
DMSO
M01(d)
M02(m)
M03(m)
M04(m)
M05(m)
M06(m)
M07(m)
M08(m)
M09(d)
M10(m)
TMS
Normalized Intensity
Chemical Shift (ppm

2c

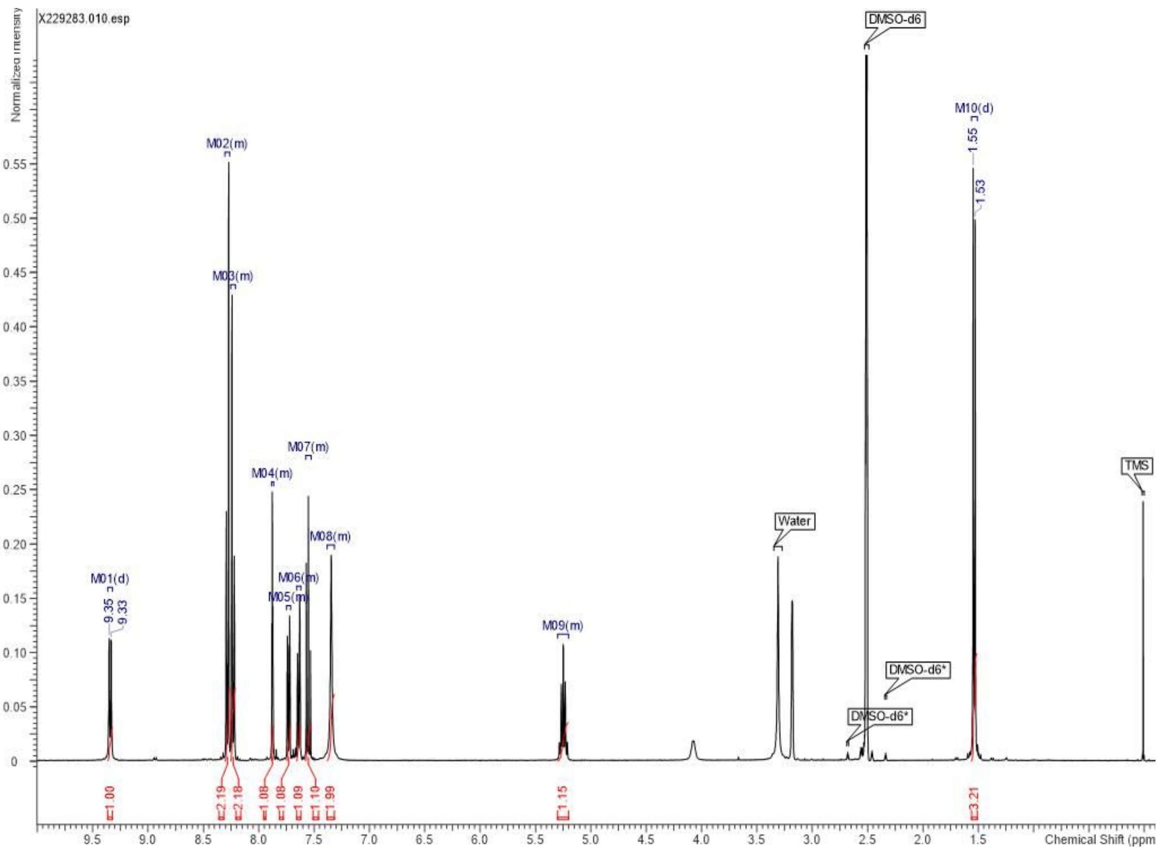
X229283.010.esp
Normalized Intensity
DMSO-d6
M10(d)
1.55
1.53
M02(m)
M03(m)
M07(m)
M04(m)
M08(m)
Water
M06(m)
M05(m)
M01(d)
9.35
9.33
M09(m)
DMSO-d6*
DMSO-d6*
TMS
1.00
2.19
2.18
1.08
1.08
1.09
1.10
1.99
1.15
3.21
Chemical Shift (ppm

2d

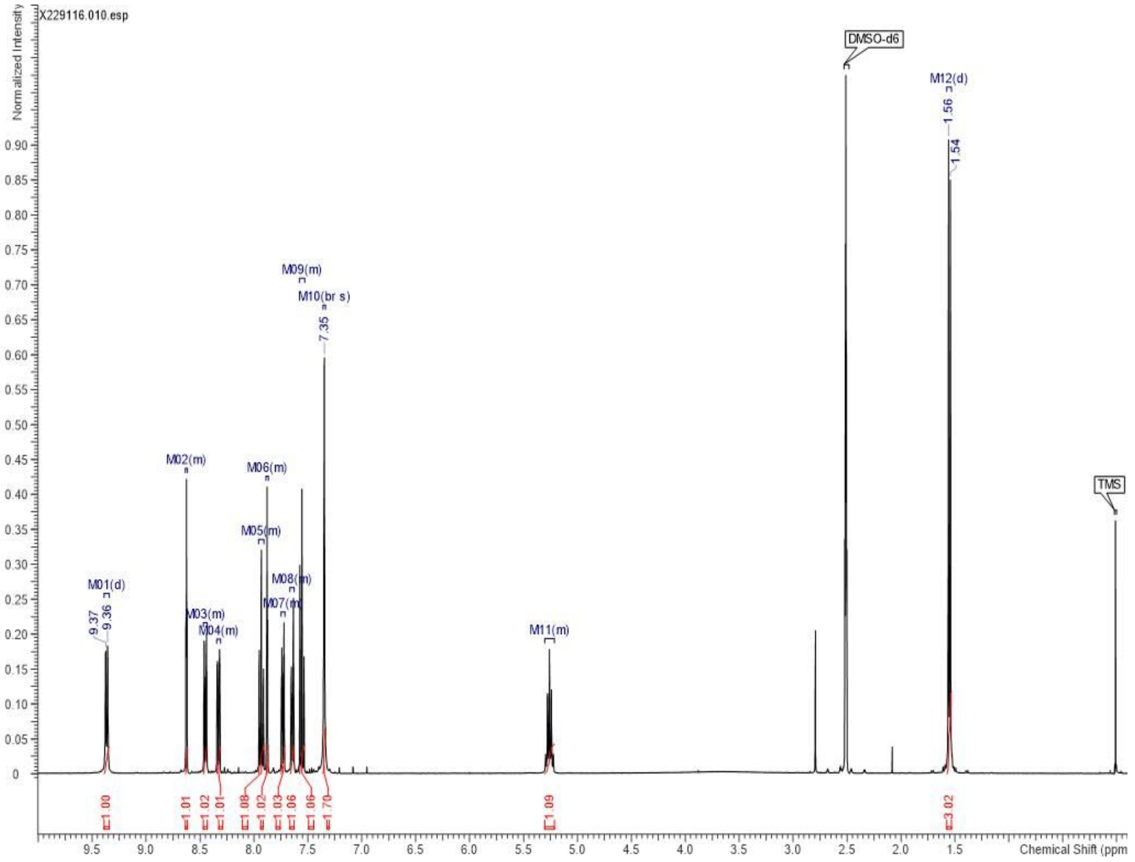
X229116.010.esp
Normalized Intensity
DMSO-d6
M12(d)
1.56
1.54
M09(m)
M10(br s)
7.35
M02(m)
M06(m)
M05(m)
M08(m)
M07(m)
M01(d)
9.37
9.36
M03(m)
M04(m)
M11(m)
TMS
1.00
1.01
1.02
1.01
1.08
1.02
1.03
1.06
1.06
1.70
1.09
3.02
Chemical Shift (ppm

2e

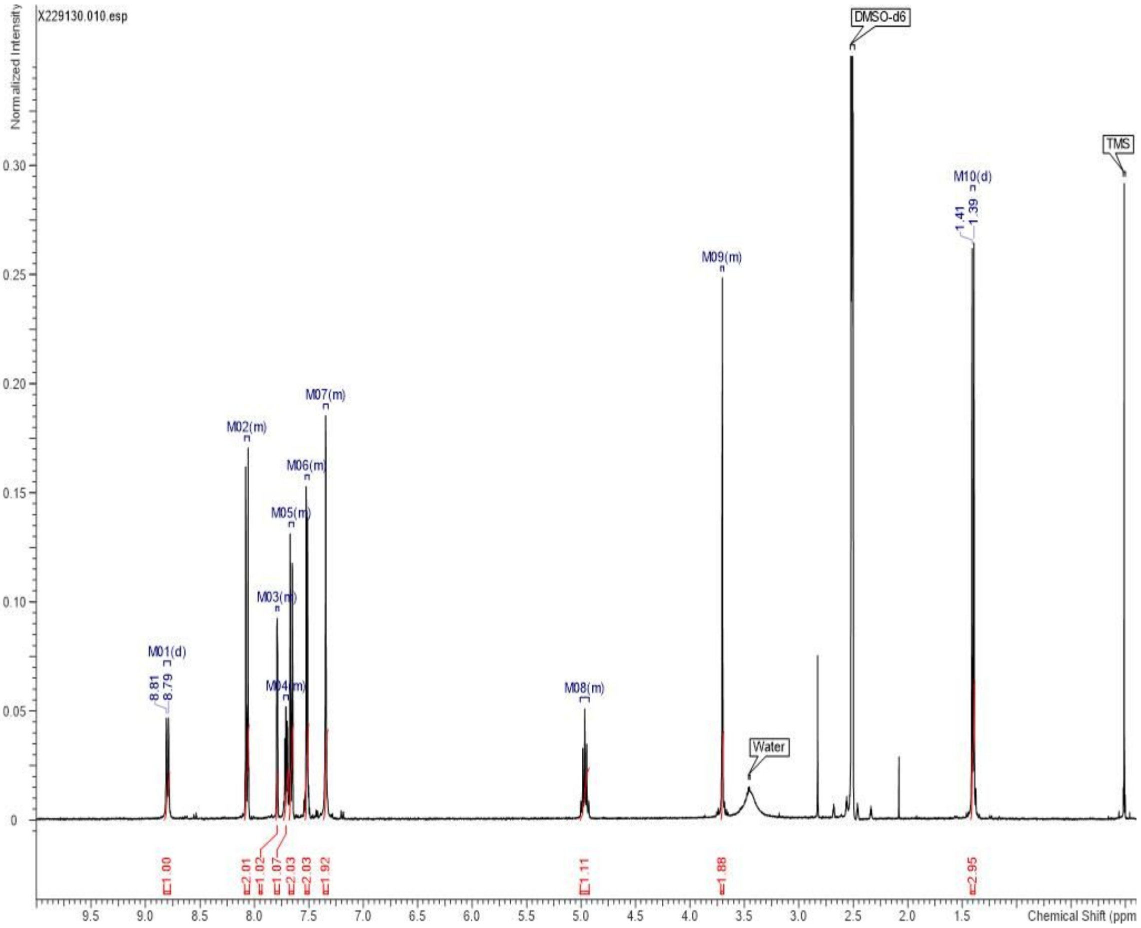
X229130.010.esp
Normalized Intensity
DMSO-d6
TMS
Water
M01(d)
M02(m)
M03(m)
M04(m)
M05(m)
M06(m)
M07(m)
M08(m)
M09(m)
M10(d)
8.81
8.79
1.41
1.39
1.00
2.01
1.02
1.07
2.03
2.03
1.92
1.11
1.88
2.95
Chemical Shift (ppm

2f

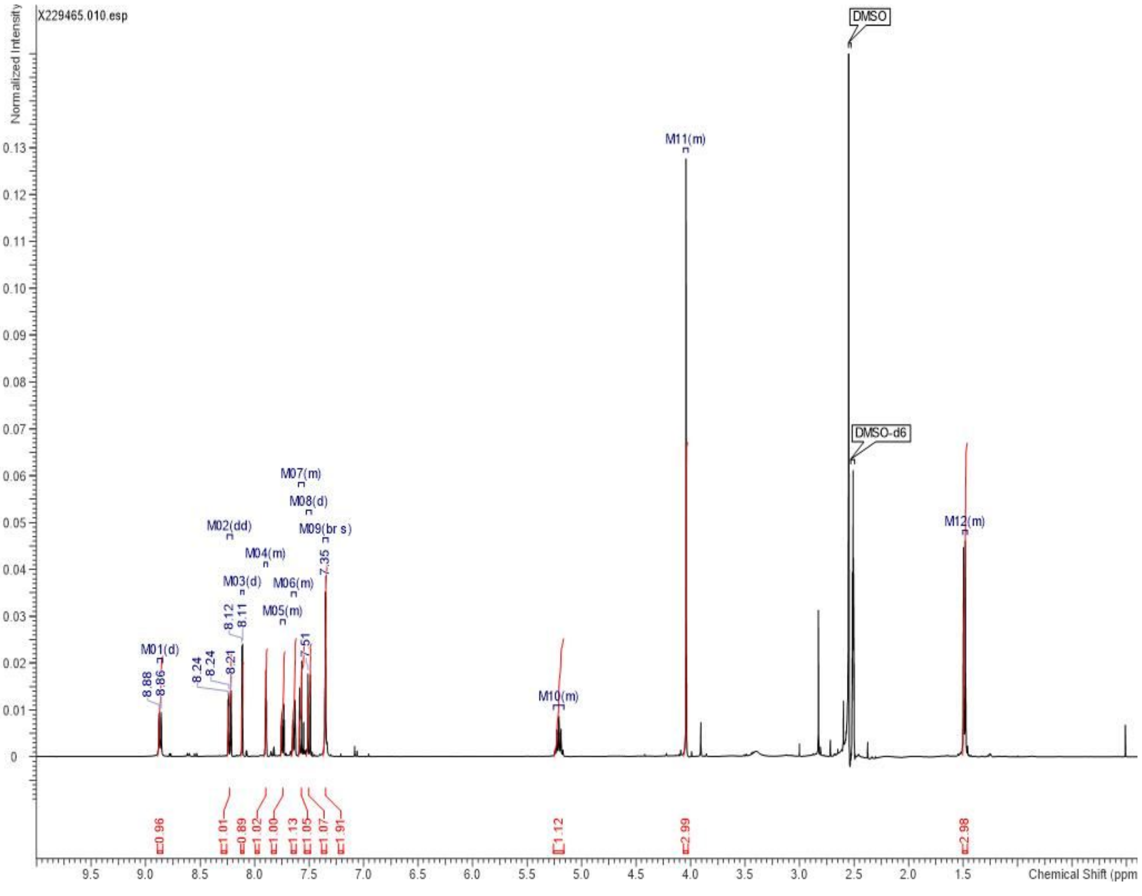
X229465.010.esp
Normalized Intensity
DMSO
DMSO-d6
M01(d)
M02(dd)
M03(d)
M04(m)
M05(m)
M06(m)
M07(m)
M08(d)
M09(br s)
M10(m)
M11(m)
M12(m)
8.88
8.86
8.24
8.24
8.21
8.12
8.11
7.51
7.35
0.96
1.01
0.89
1.02
1.00
2.13
1.05
1.07
1.91
1.12
2.99
2.98
Chemical Shift (ppm

2g

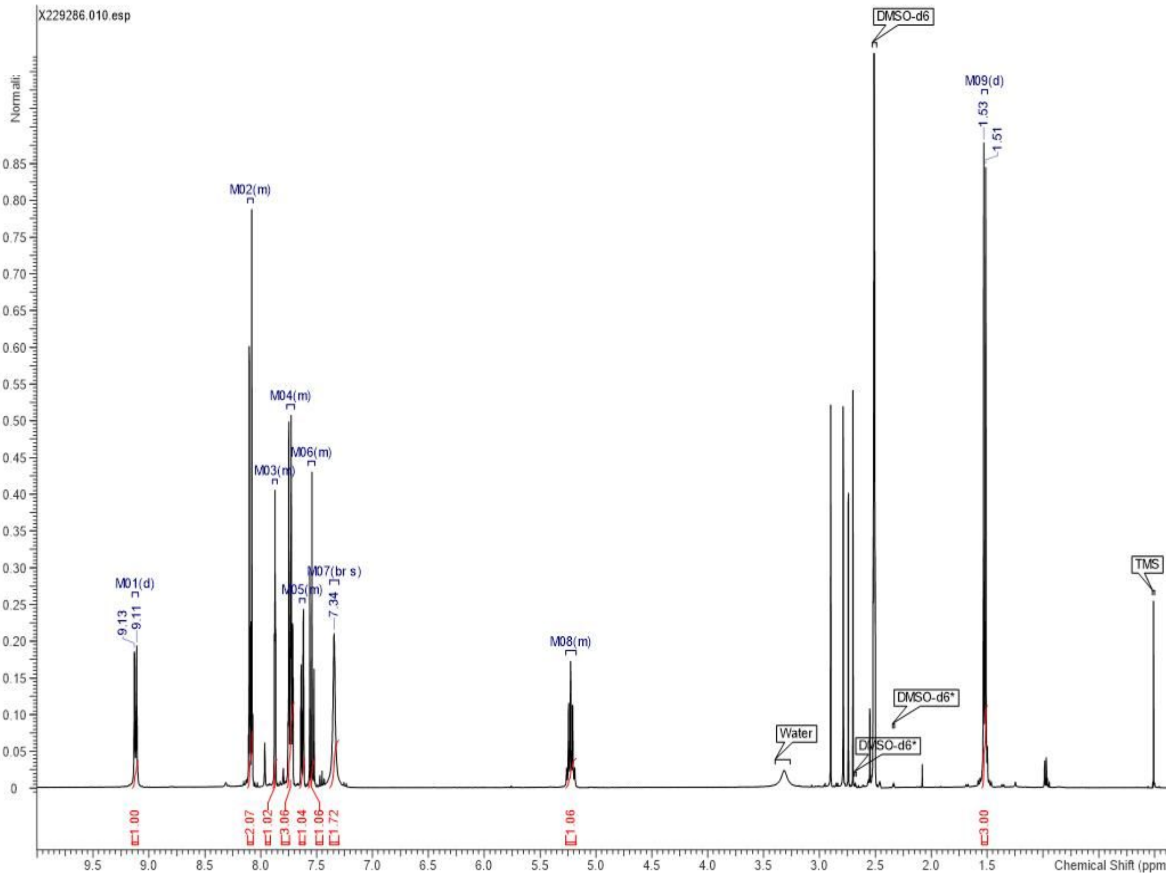
X229286.010.esp
Normali
DMSO-d6
M09(d)
1.53
1.51
M02(m)
M04(m)
M06(m)
M03(m)
M01(d)
9.13
9.11
M05(m)
M07(br s)
7.34
M08(m)
Water
DMSO-d6*
DMSO-d6*
TMS
1.00
2.07
1.02
3.06
1.04
1.06
1.72
1.06
3.00
Chemical Shift (ppm

2h

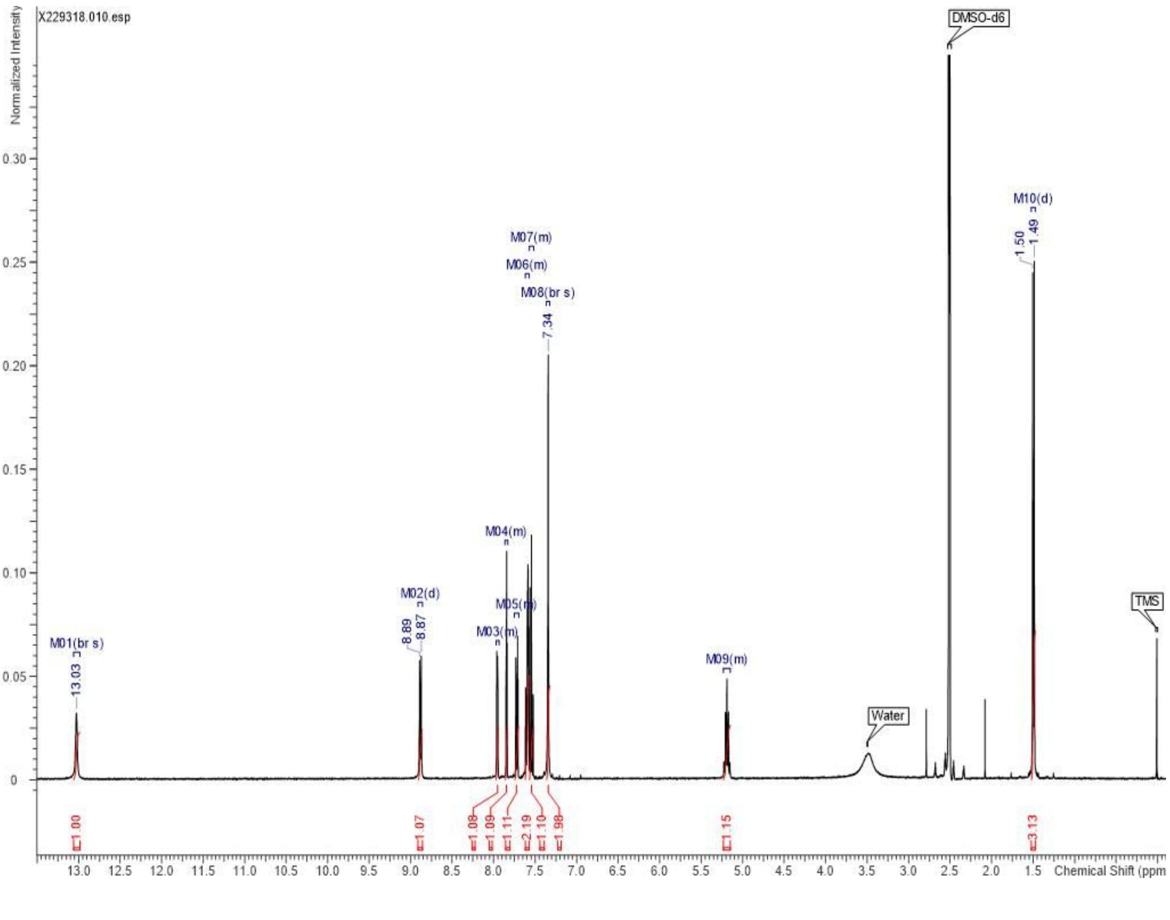
X229318.010.esp
Normalized Intensity
DMSO-d6
M10(d)
1.50
1.49
M07(m)
M06(m)
M08(br s)
7.34
M04(m)
M02(d)
8.89
8.87
M05(m)
M03(m)
M01(br s)
13.03
M09(m)
Water
TMS
1.00
1.07
1.08
1.09
1.11
2.19
1.10
1.98
1.15
3.13
Chemical Shift (ppm

2i

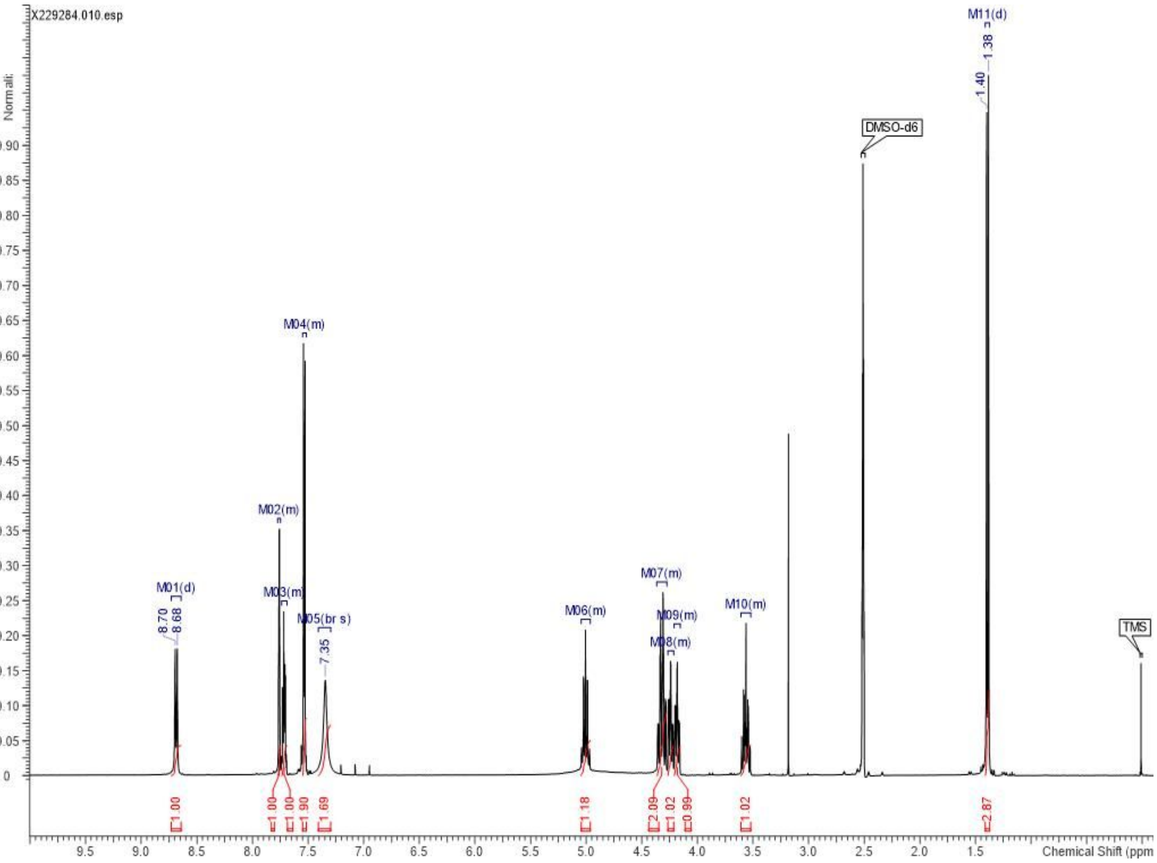
X229284.010.esp
Normali.
M01(d)
8.70
8.68
M02(m)
M03(m)
M04(m)
M05(br s)
7.35
M06(m)
M07(m)
M08(m)
M09(m)
M10(m)
DMSO-d6
M11(d)
1.40
1.38
TMS
1.00
1.00
1.00
1.90
1.69
1.18
2.09
1.02
0.99
1.02
2.87
Chemical Shift (ppm

5a

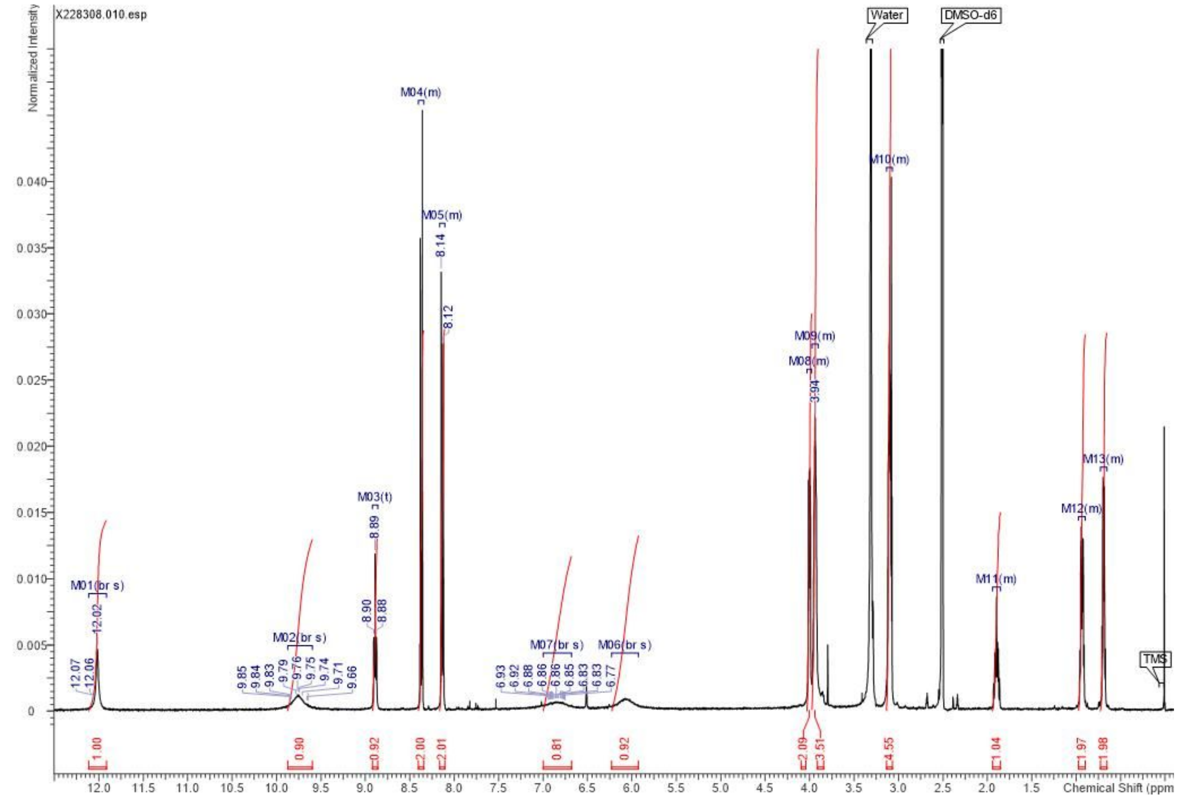

X228308.010.esp
Normalized Intensity
Water
DMSO-d6
TMS
M01(br s)
M02(br s)
M03(t)
M04(m)
M05(m)
M06(br s)
M07(br s)
M08(m)
M09(m)
M10(m)
M11(m)
M12(m)
M13(m)
Chemical Shift (ppm

5b

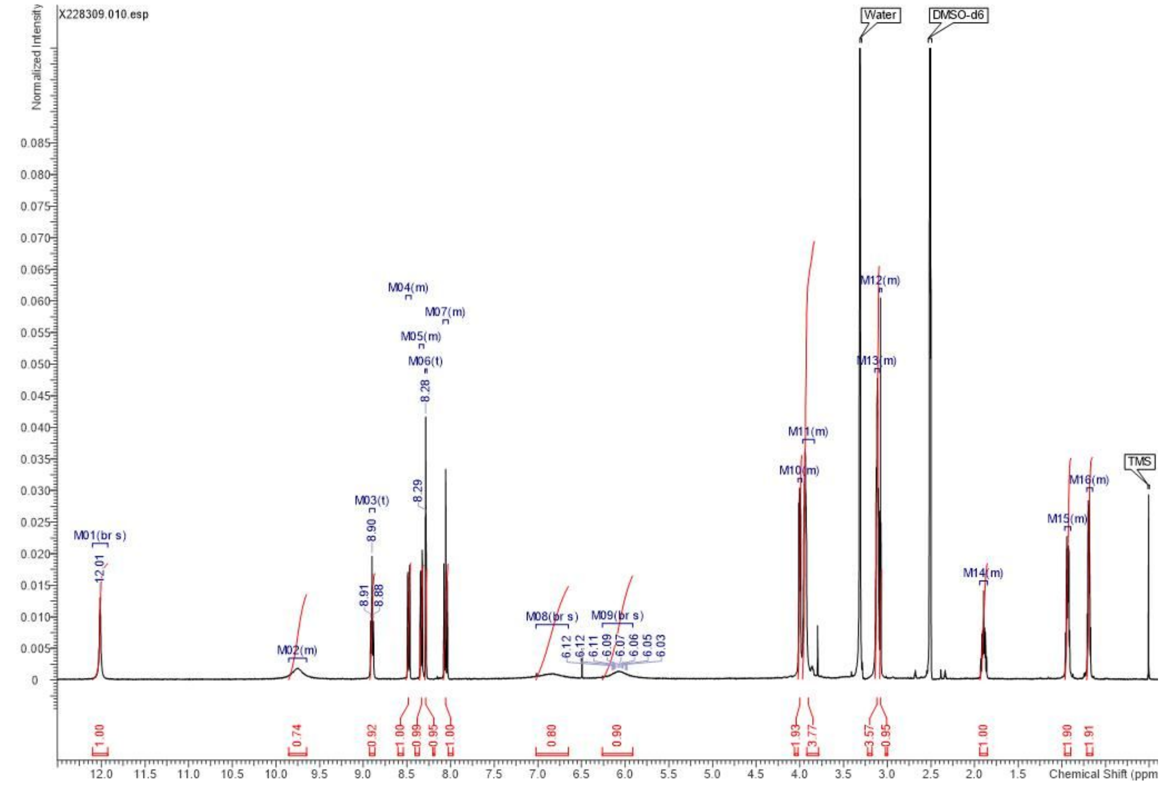

X228309.010.esp
Normalized Intensity
Water
DMSO-d6
TMS
M01(br s)
M02(m)
M03(t)
M04(m)
M05(m)
M06(t)
M07(m)
M08(br s)
M09(br s)
M10(m)
M11(m)
M12(m)
M13(m)
M14(m)
M15(m)
M16(m)
Chemical Shift (ppm

5c

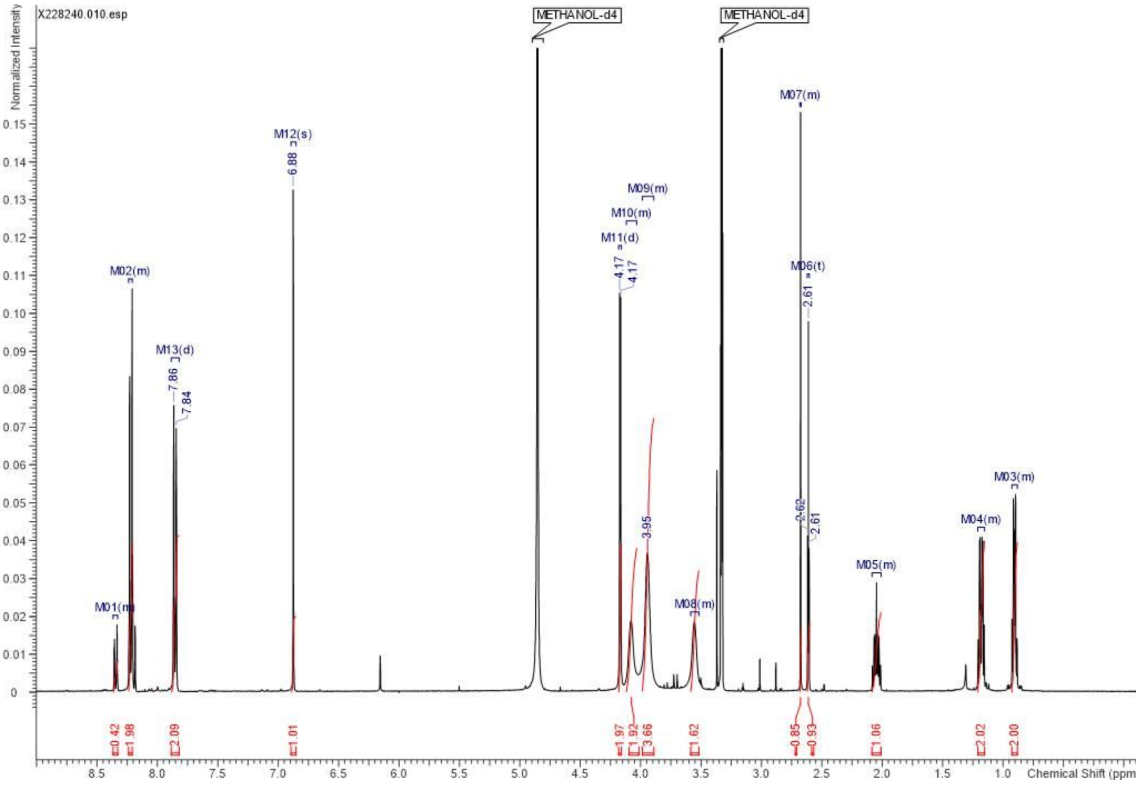
X228240.010.esp
Normalized Intensity
METHANOL-d4
METHANOL-d4
M07(m)
M12(s)
6.88
M09(m)
M10(m)
M11(d)
4.17
4.17
M02(m)
M06(t)
2.61
M13(d)
7.86
7.84
3.95
2.62
2.61
M04(m)
M03(m)
M05(m)
M01(m)
M08(m)
0.42
1.98
2.09
1.01
1.97
1.92
3.66
1.62
0.85
0.93
1.06
2.02
2.00
Chemical Shift (ppm)

5d

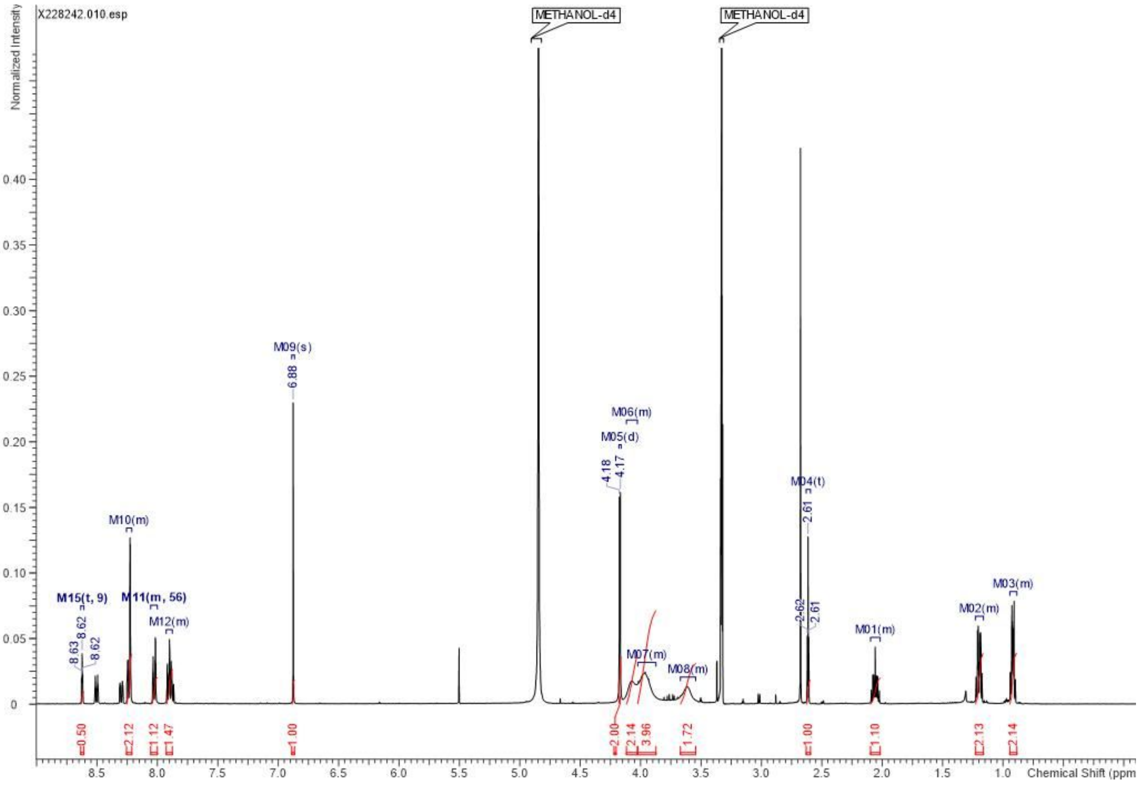
X228242.010.esp
Normalized Intensity
METHANOL-d4
METHANOL-d4
M09(s)
6.88
M06(m)
M05(d)
4.18
4.17
M04(t)
2.61
M10(m)
M15(t, 9)
M11(m, 56)
M12(m)
8.63
8.62
8.62
2.62
2.61
M01(m)
M02(m)
M03(m)
M07(m)
M08(m)
0.50
2.12
1.12
1.47
1.00
2.00
2.14
3.96
1.72
1.00
1.10
2.13
2.14
Chemical Shift (ppm)

5e

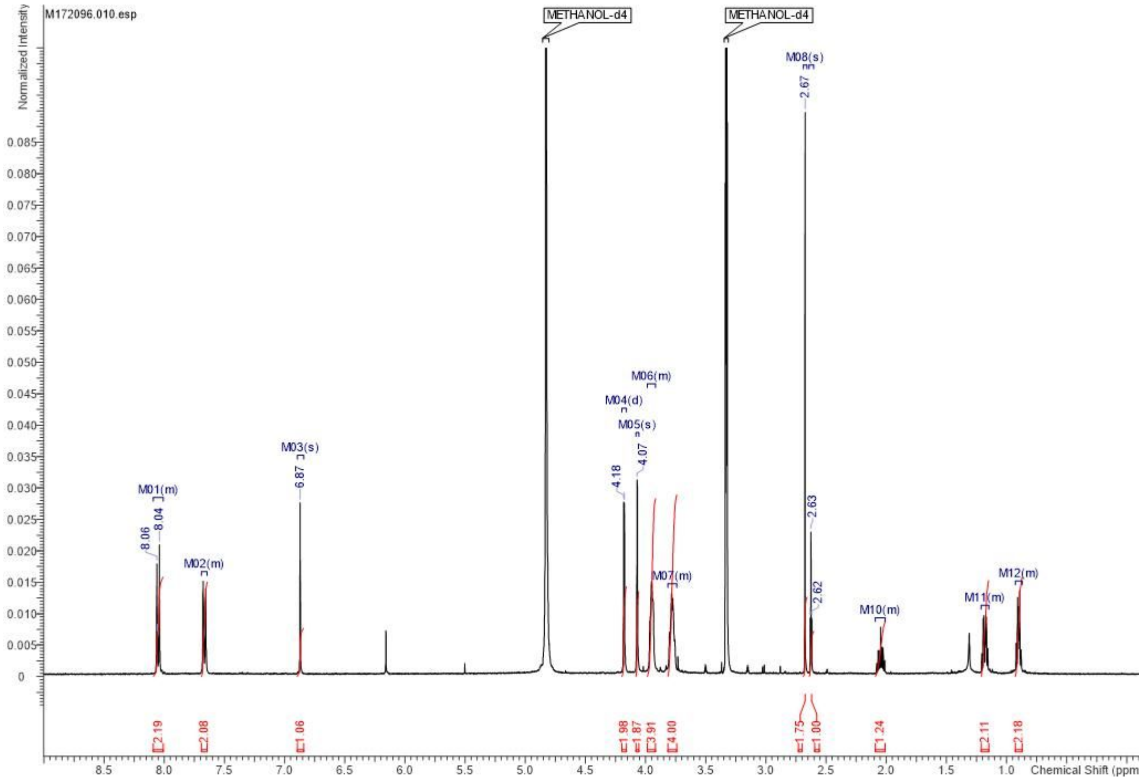

5f

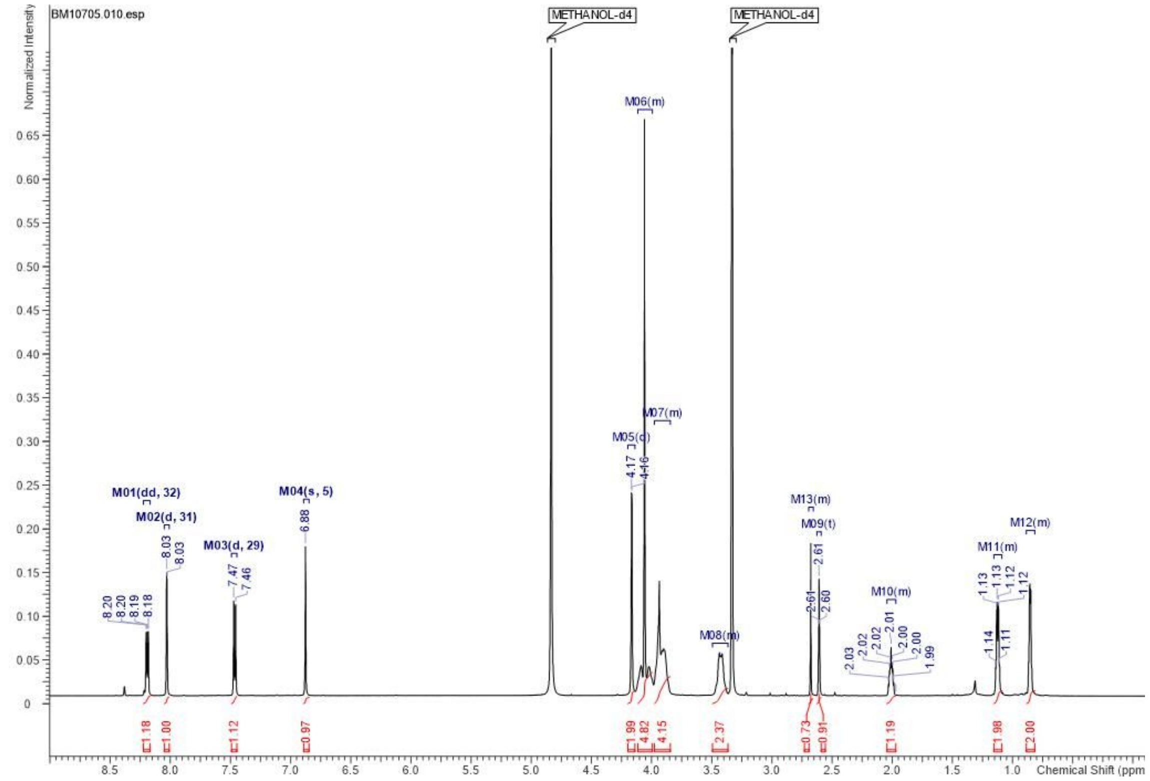

5g

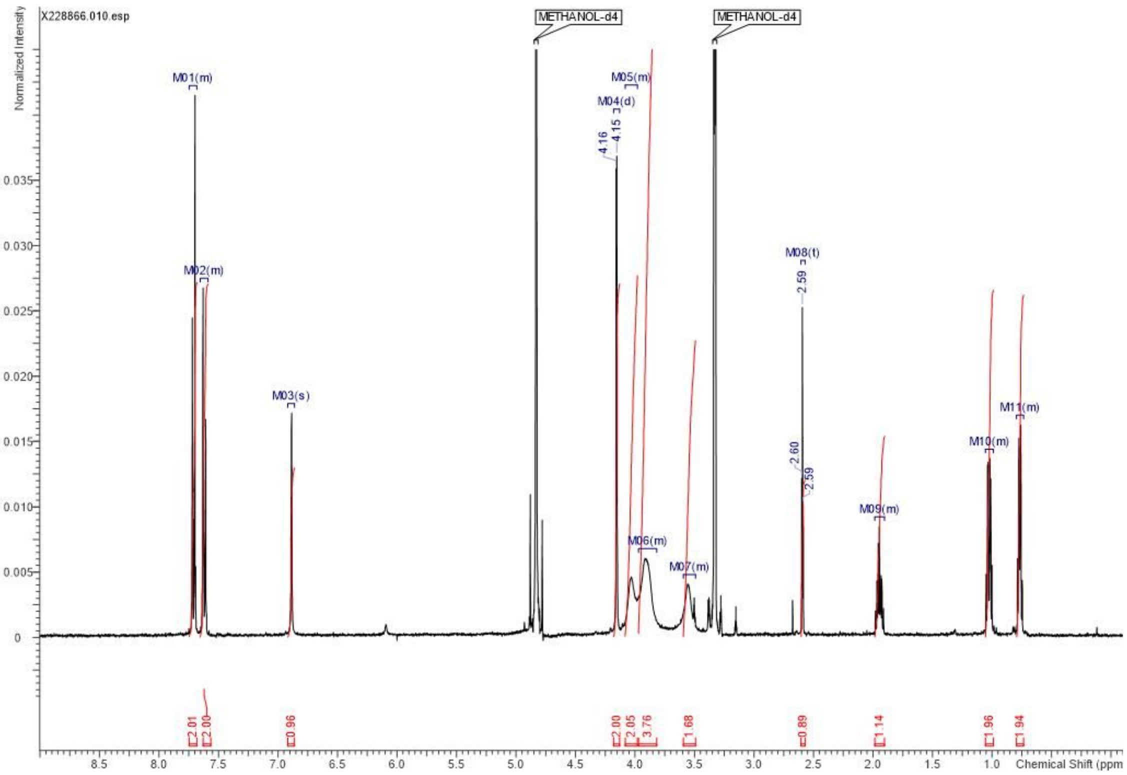
X228866.010.esp
METHANOL-d4
METHANOL-d4
Normalized Intensity
Chemical Shift (ppm)

5h

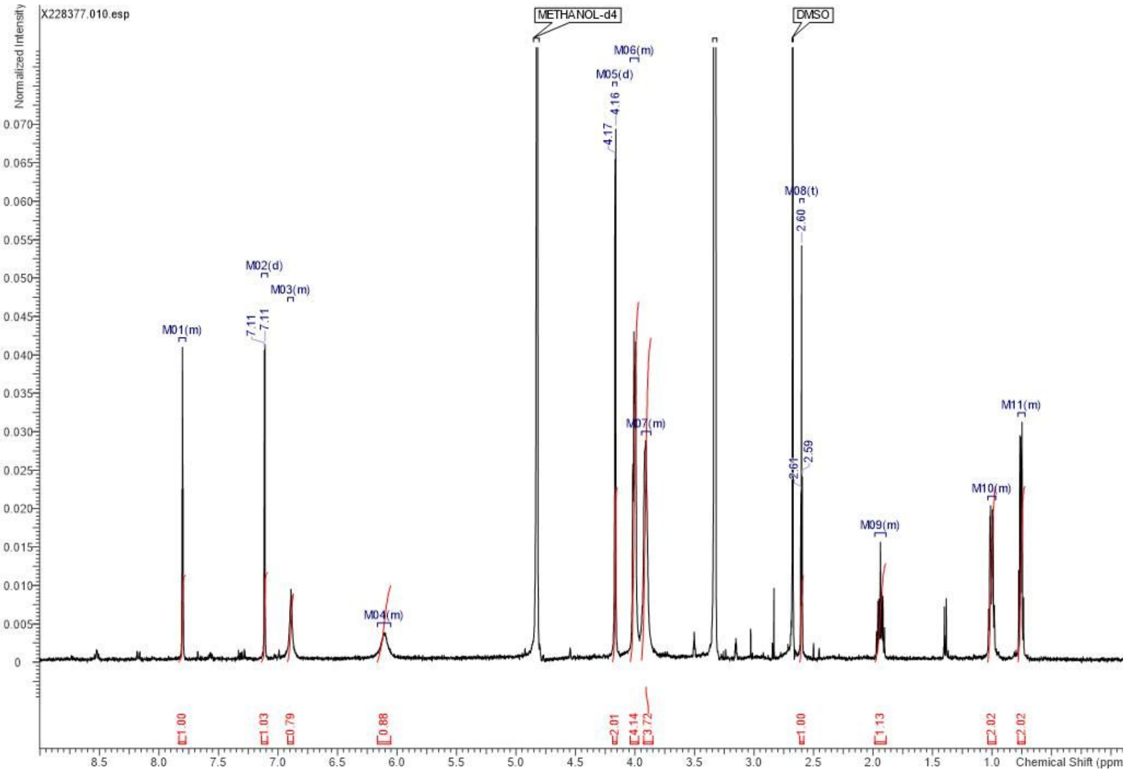
X228377.010.esp
METHANOL-d4
DMSO
Normalized Intensity
Chemical Shift (ppm)

5i

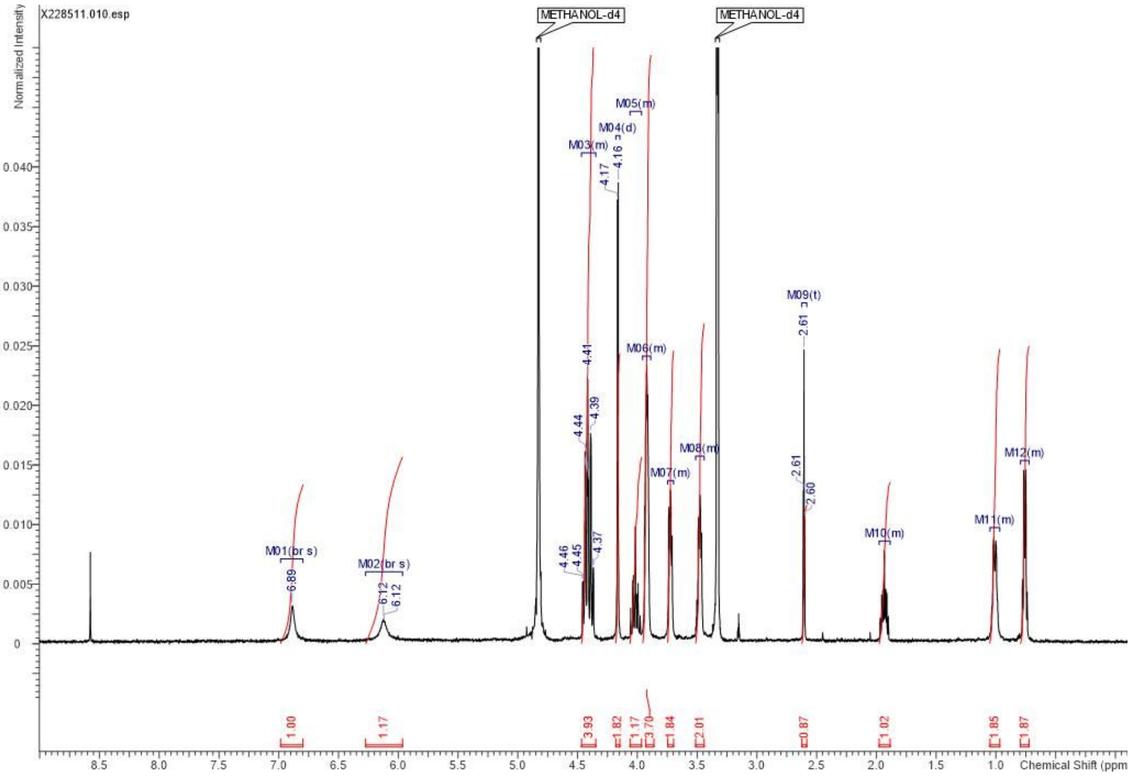

X228511.010.esp
Normalized Intensity
METHANOL-d4
METHANOL-d4
M01(br s)
M02(br s)
M03(m)
M04(d)
M05(m)
M06(m)
M07(m)
M08(m)
M09(t)
M10(m)
M11(m)
M12(m)
Chemical Shift (ppm)

10. ^{19}F NMR spectra

1a

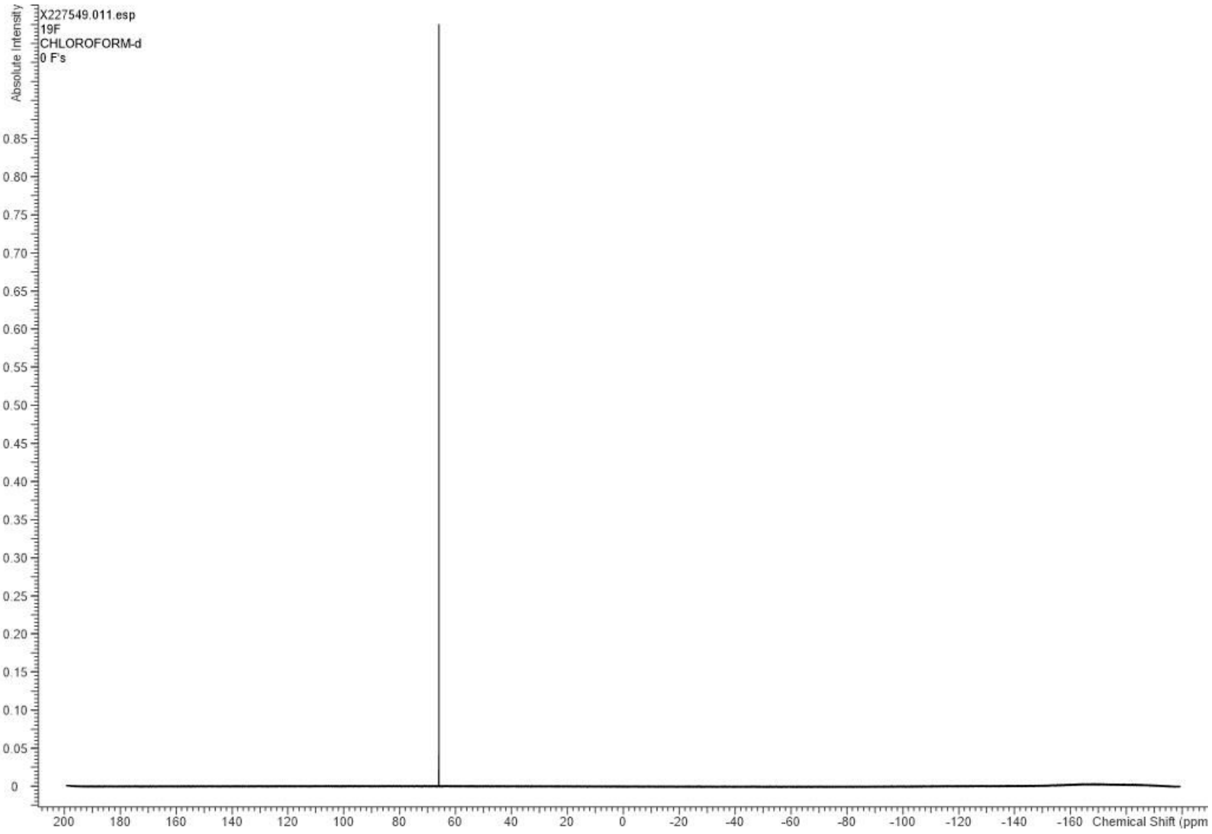

1b

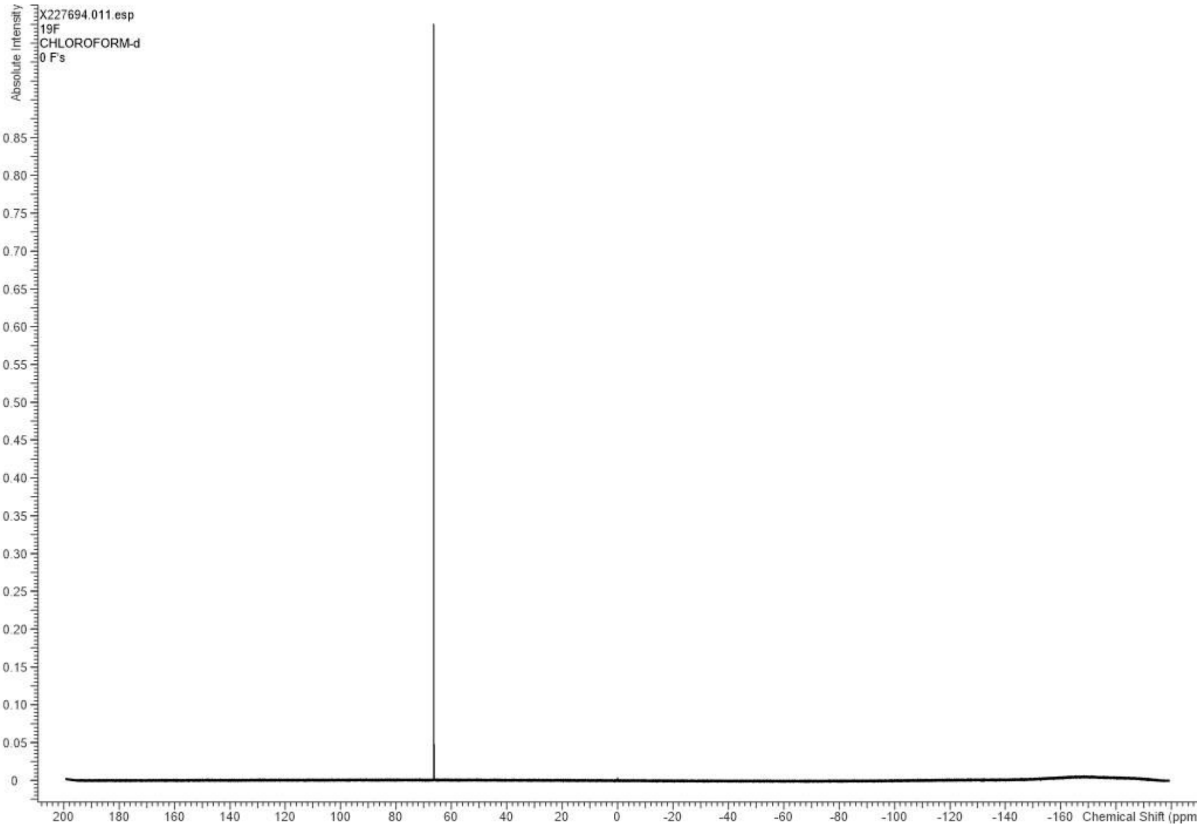

1c

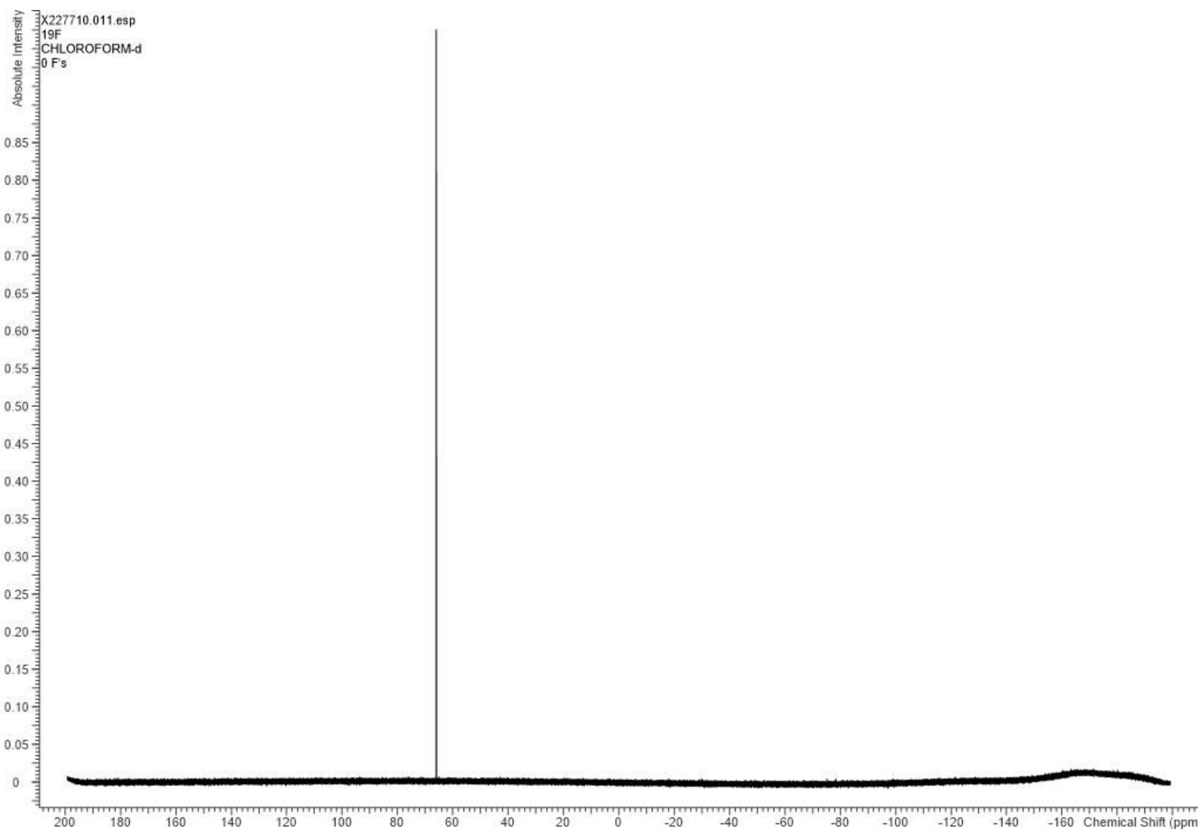

X227710.011.esp
19F
CHLOROFORM-d
0 F's
Absolute Intensity
Chemical Shift (ppm

1d

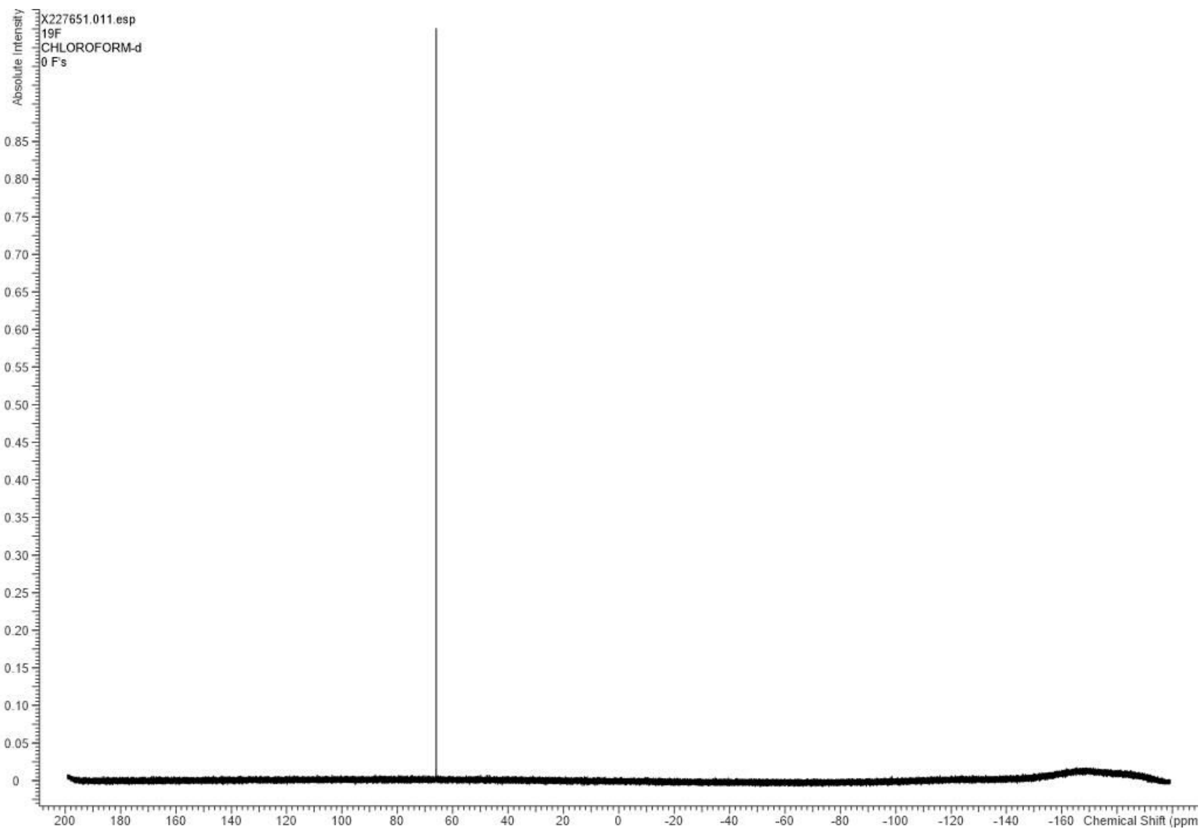

X227651.011.esp
19F
CHLOROFORM-d
0 F's
Absolute Intensity
Chemical Shift (ppm

1e

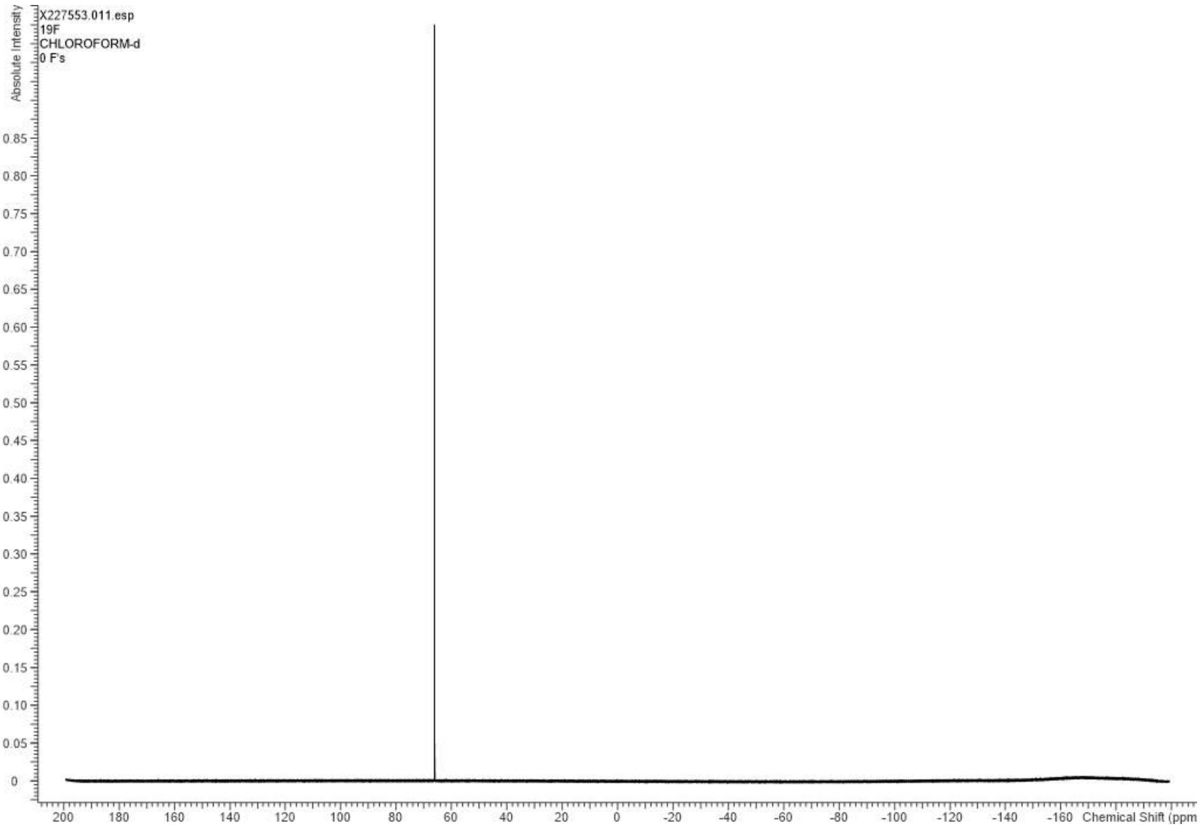
X227553.011.esp
19F
CHLOROFORM-d
0 F's
Absolute Intensity
0.85
0.80
0.75
0.70
0.65
0.60
0.55
0.50
0.45
0.40
0.35
0.30
0.25
0.20
0.15
0.10
0.05
0
200 180 160 140 120 100 80 60 40 20 0 -20 -40 -60 -80 -100 -120 -140 -160 Chemical Shift (ppm

1f

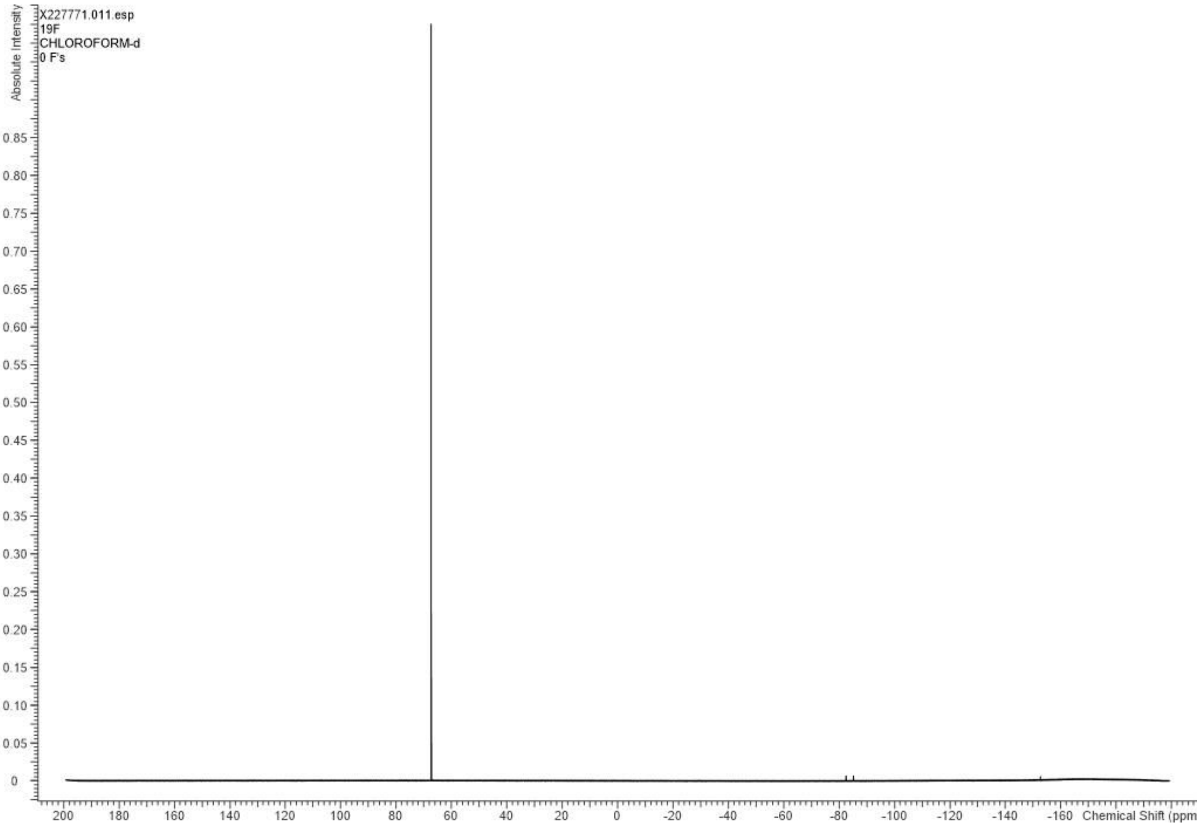
X227771.011.esp
19F
CHLOROFORM-d
0 F's
Absolute Intensity
0.85
0.80
0.75
0.70
0.65
0.60
0.55
0.50
0.45
0.40
0.35
0.30
0.25
0.20
0.15
0.10
0.05
0
200 180 160 140 120 100 80 60 40 20 0 -20 -40 -60 -80 -100 -120 -140 -160 Chemical Shift (ppm

1g

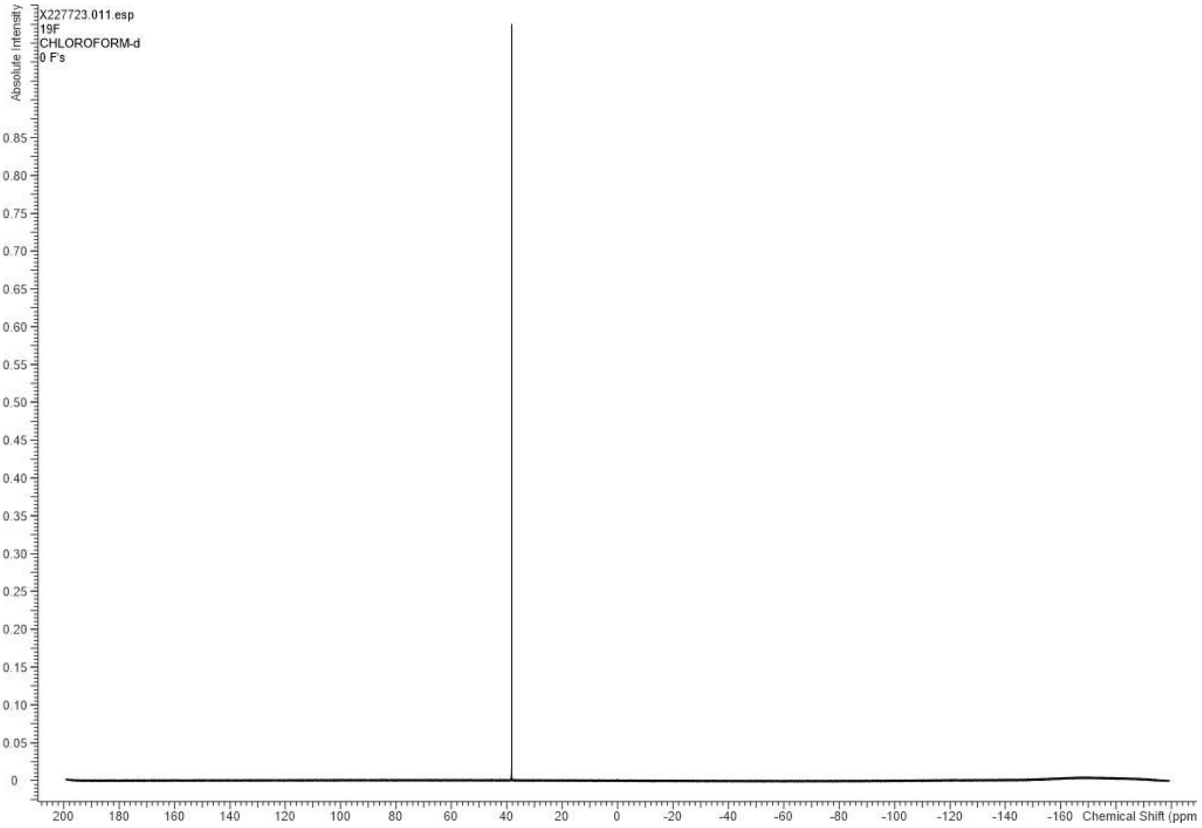
X227723.011.esp
19F
CHLOROFORM-d
0 F's
Absolute Intensity
0.85
0.80
0.75
0.70
0.65
0.60
0.55
0.50
0.45
0.40
0.35
0.30
0.25
0.20
0.15
0.10
0.05
0
200 180 160 140 120 100 80 60 40 20 0 -20 -40 -60 -80 -100 -120 -140 -160
Chemical Shift (ppm

1h

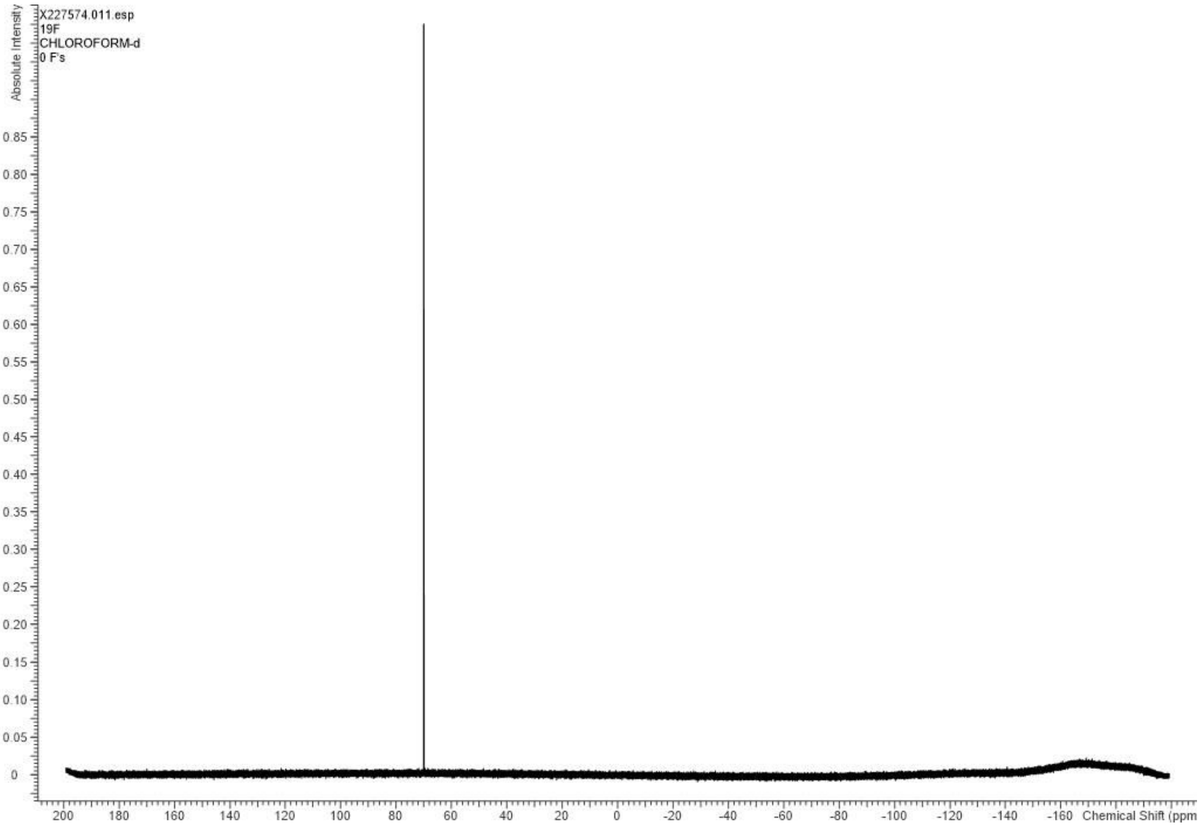
X227574.011.esp
19F
CHLOROFORM-d
0 F's
Absolute Intensity
0.85
0.80
0.75
0.70
0.65
0.60
0.55
0.50
0.45
0.40
0.35
0.30
0.25
0.20
0.15
0.10
0.05
0
200 180 160 140 120 100 80 60 40 20 0 -20 -40 -60 -80 -100 -120 -140 -160
Chemical Shift (ppm

1i

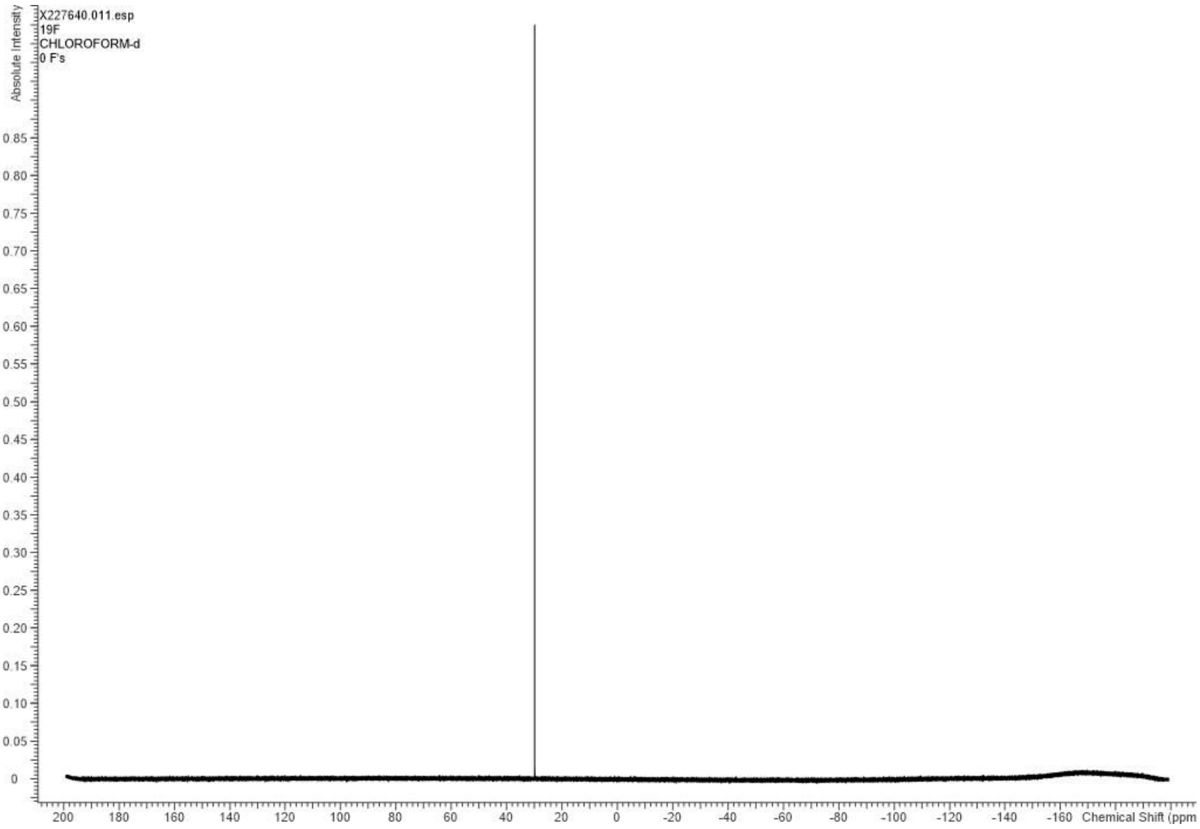
X227640.011.esp
19F
CHLOROFORM-d
0 F's
Absolute Intensity
0.85
0.80
0.75
0.70
0.65
0.60
0.55
0.50
0.45
0.40
0.35
0.30
0.25
0.20
0.15
0.10
0.05
0
200 180 160 140 120 100 80 60 40 20 0 -20 -40 -60 -80 -100 -120 -140 -160
Chemical Shift (ppm

2a

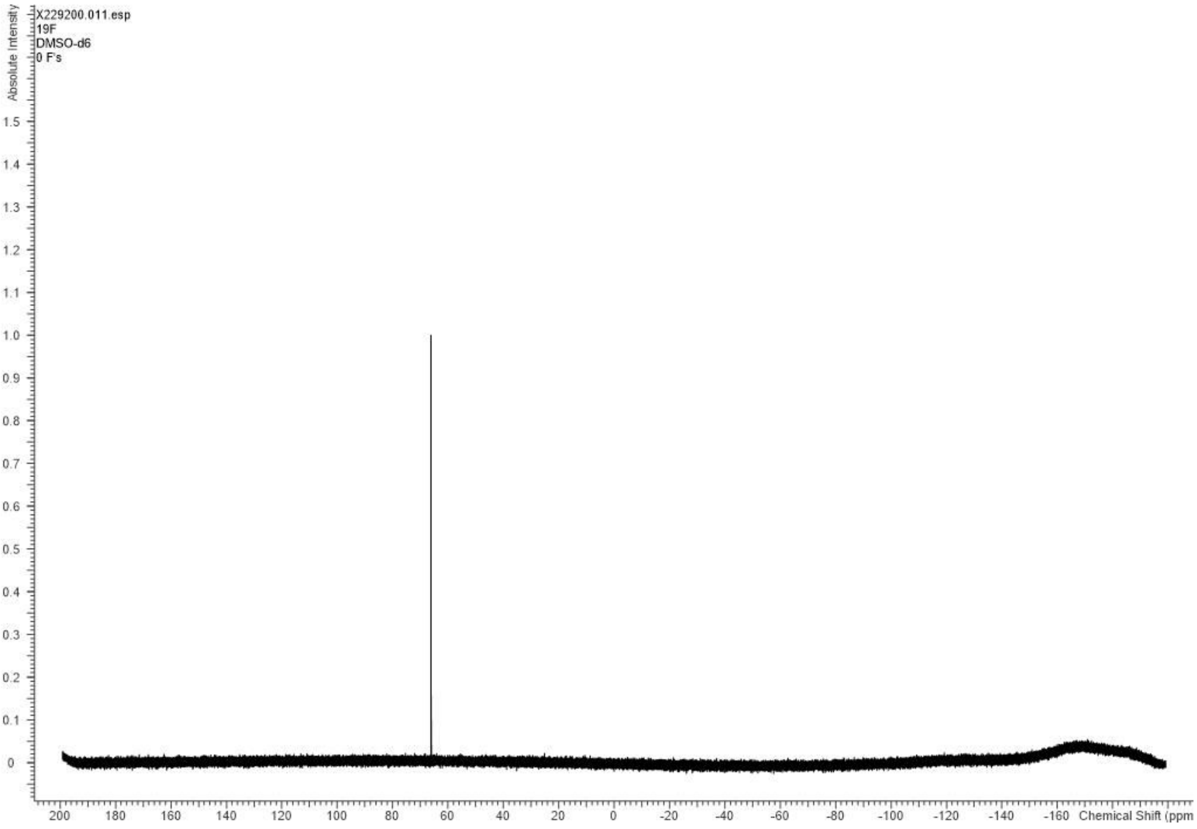
X229200.011.esp
19F
DMSO-d6
0 F's
Absolute Intensity
1.5
1.4
1.3
1.2
1.1
1.0
0.9
0.8
0.7
0.6
0.5
0.4
0.3
0.2
0.1
0
200 180 160 140 120 100 80 60 40 20 0 -20 -40 -60 -80 -100 -120 -140 -160
Chemical Shift (ppm

2b

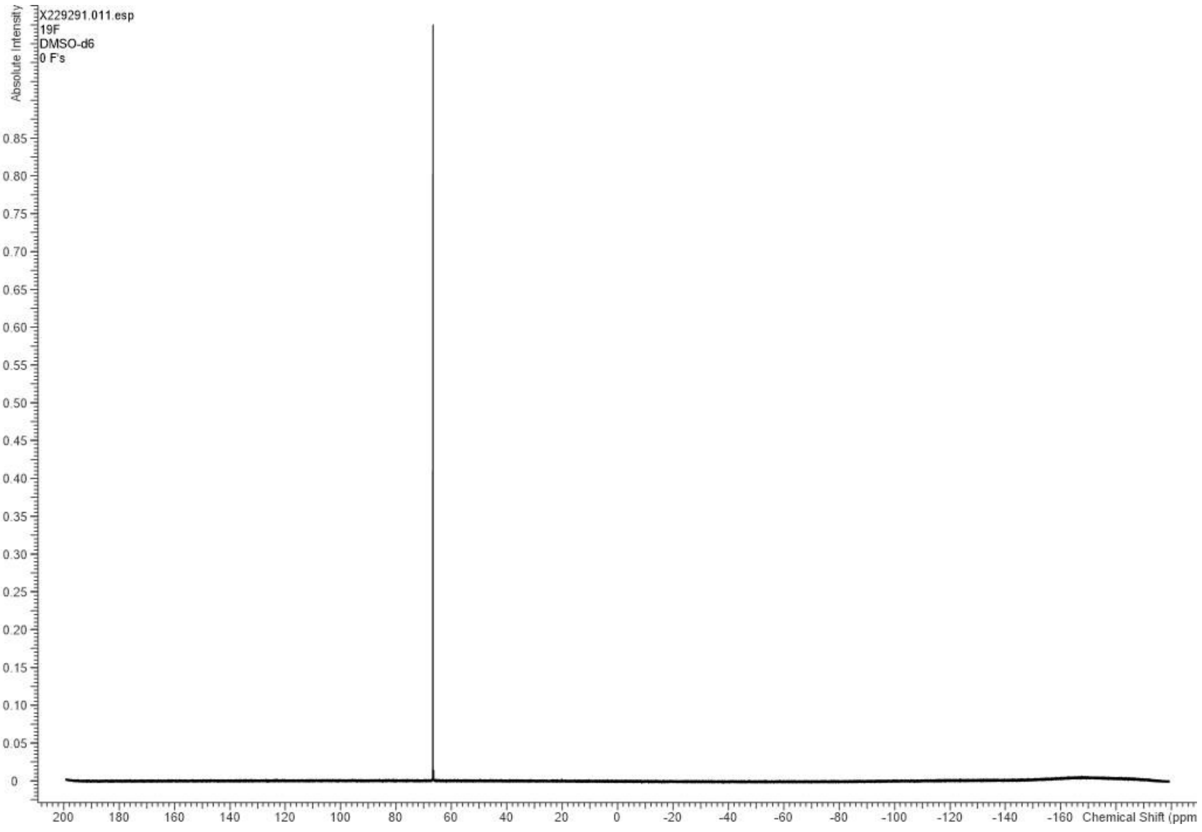

X229291.011.esp
19F
DMSO-d6
0 F's
Absolute Intensity
Chemical Shift (ppm

2c

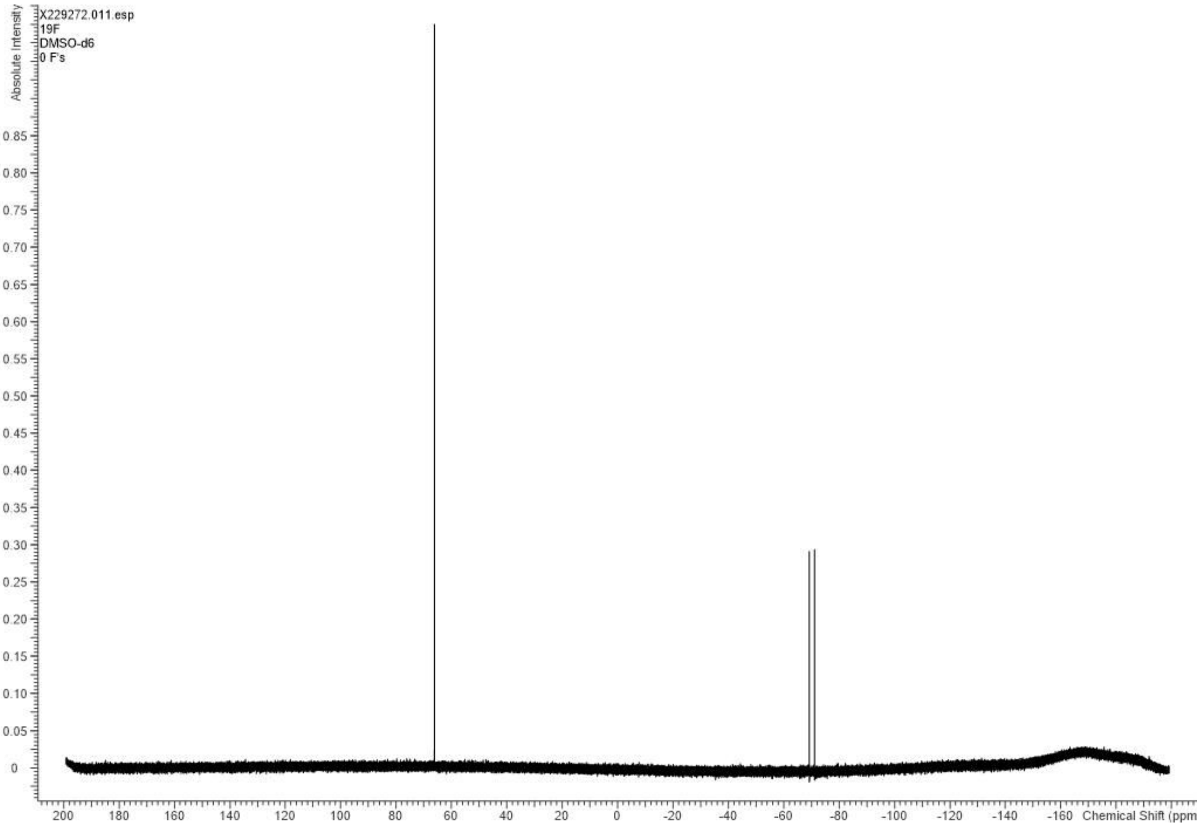

X229272.011.esp
19F
DMSO-d6
0 F's
Absolute Intensity
Chemical Shift (ppm

2d

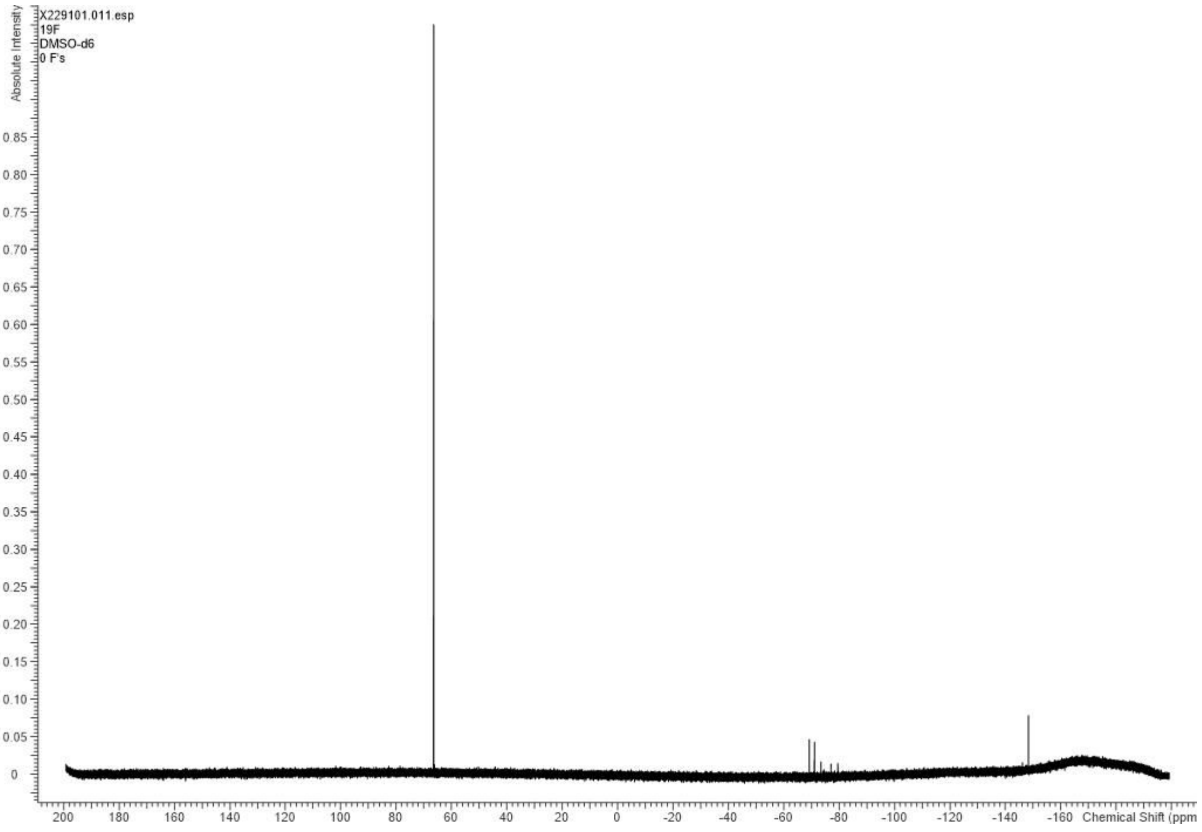

X229101.011.esp
19F
DMSO-d6
0 F's
Absolute Intensity
0.85
0.80
0.75
0.70
0.65
0.60
0.55
0.50
0.45
0.40
0.35
0.30
0.25
0.20
0.15
0.10
0.05
0
200 180 160 140 120 100 80 60 40 20 0 -20 -40 -60 -80 -100 -120 -140 -160
Chemical Shift (ppm

2e

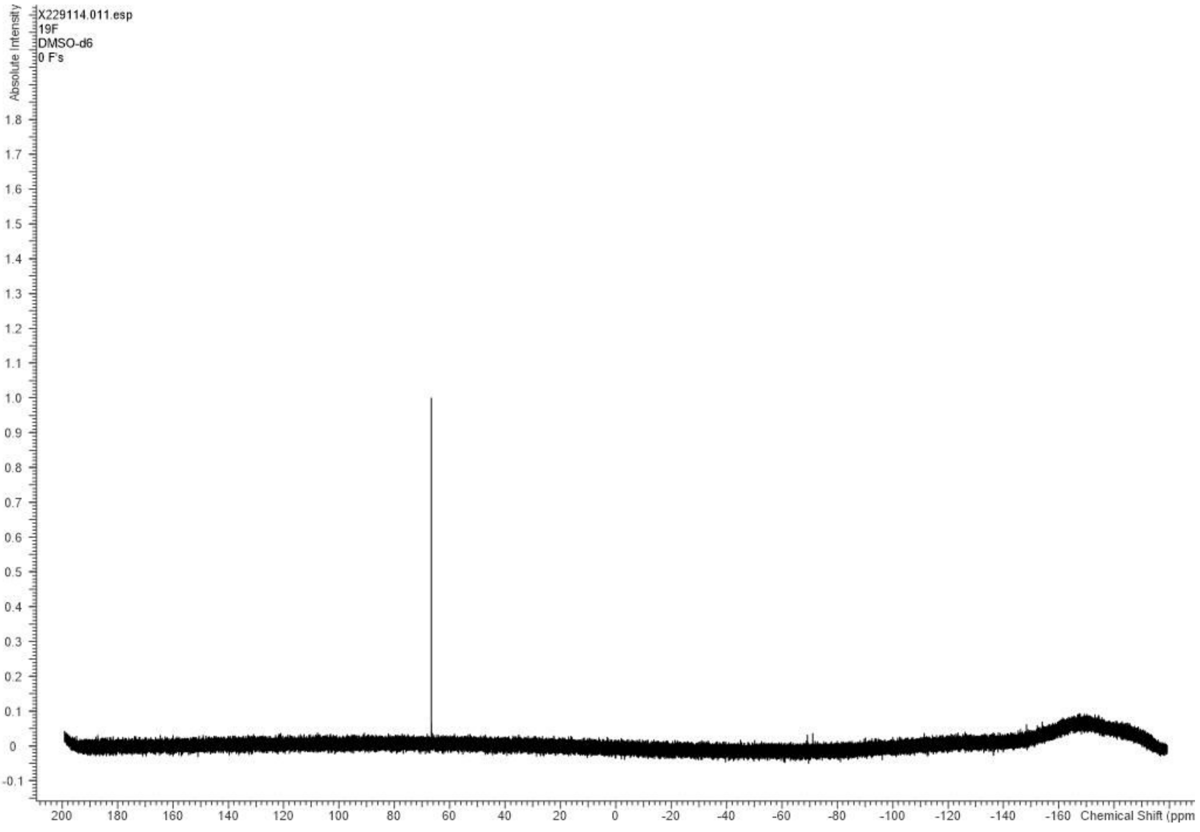

X229114.011.esp
19F
DMSO-d6
0 F's
Absolute Intensity
1.8
1.7
1.6
1.5
1.4
1.3
1.2
1.1
1.0
0.9
0.8
0.7
0.6
0.5
0.4
0.3
0.2
0.1
0
-0.1
200 180 160 140 120 100 80 60 40 20 0 -20 -40 -60 -80 -100 -120 -140 -160
Chemical Shift (ppm

2f

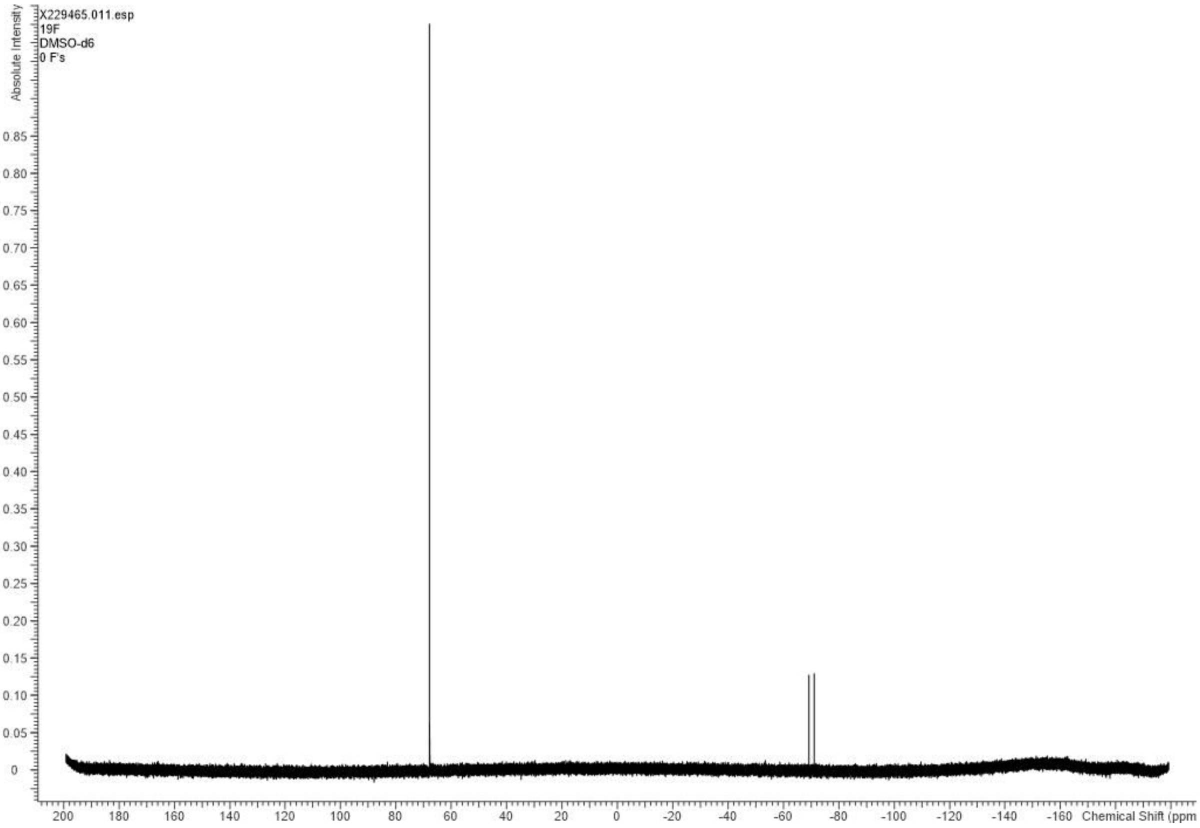
X229465.011.esp
19F
DMSO-d6
0 F's
Absolute Intensity
Chemical Shift (ppm

2g

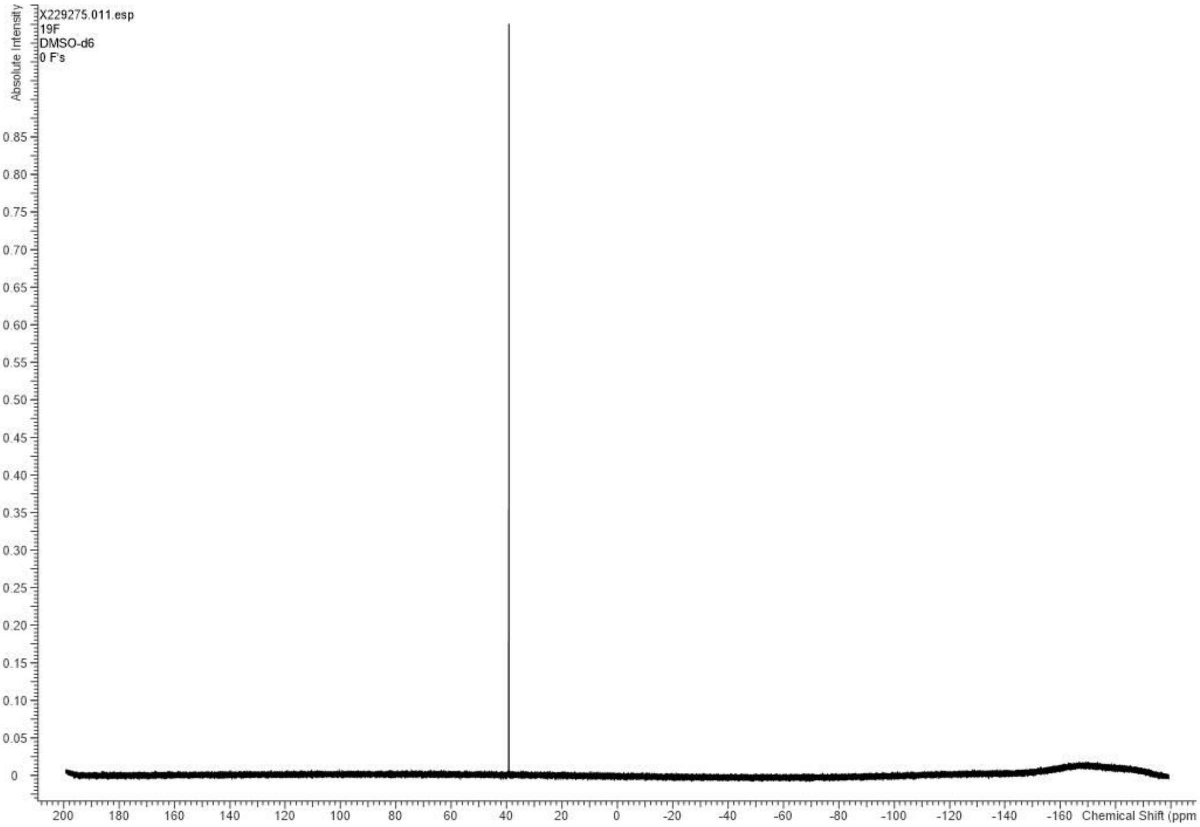
X229275.011.esp
19F
DMSO-d6
0 F's
Absolute Intensity
Chemical Shift (ppm

2h

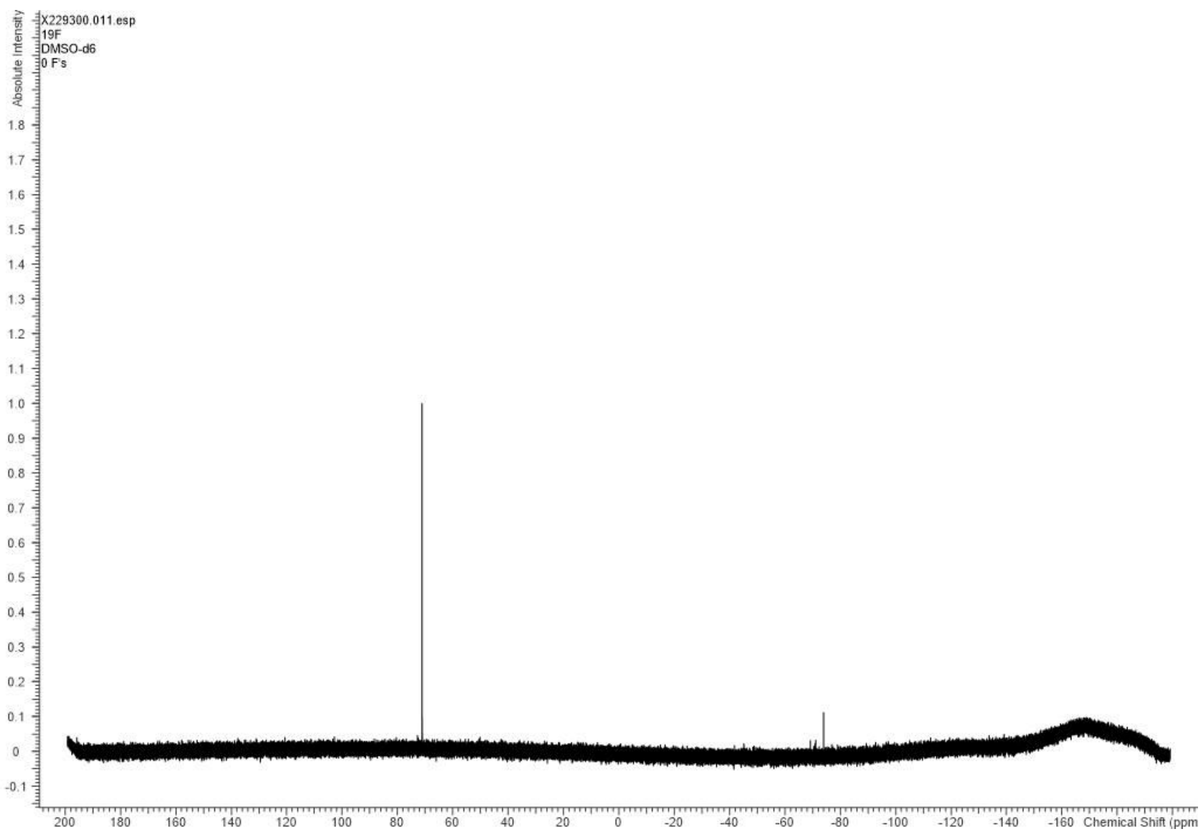
X229300.011.esp
19F
DMSO-d6
0 F's
Absolute Intensity
Chemical Shift (ppm

2i

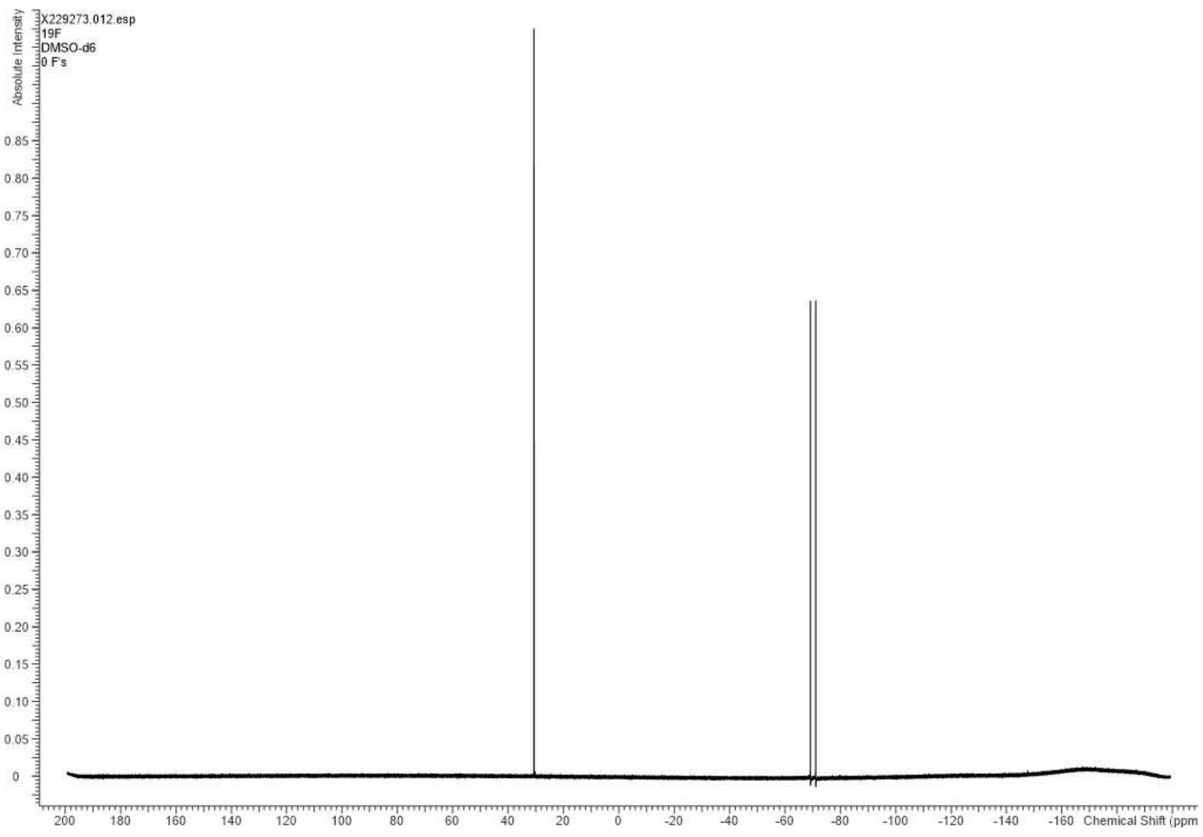
X229273.012.esp
19F
DMSO-d6
0 F's
Absolute Intensity
Chemical Shift (ppm

5a

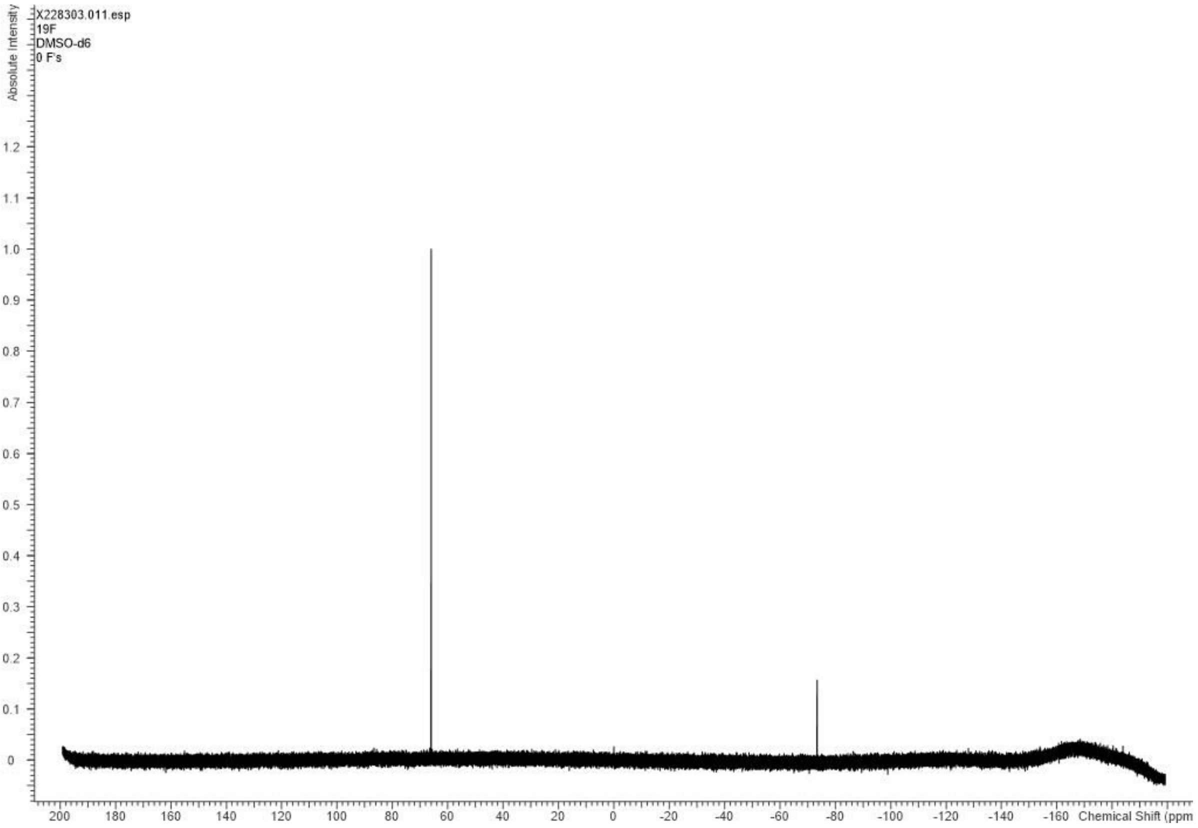
X228303.011.esp
19F
DMSO-d6
0 F's
Absolute Intensity
Chemical Shift (ppm

5b

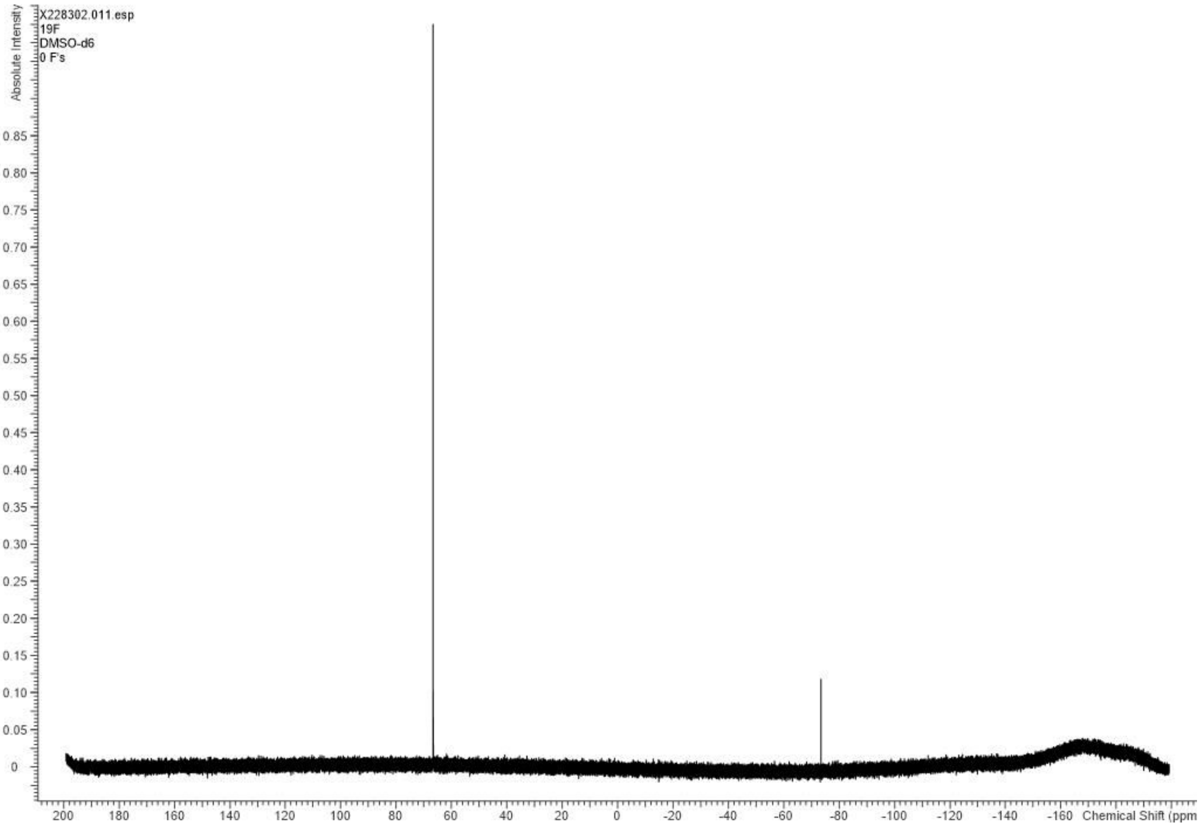
X228302.011.esp
19F
DMSO-d6
0 F's
Absolute Intensity
Chemical Shift (ppm

5c

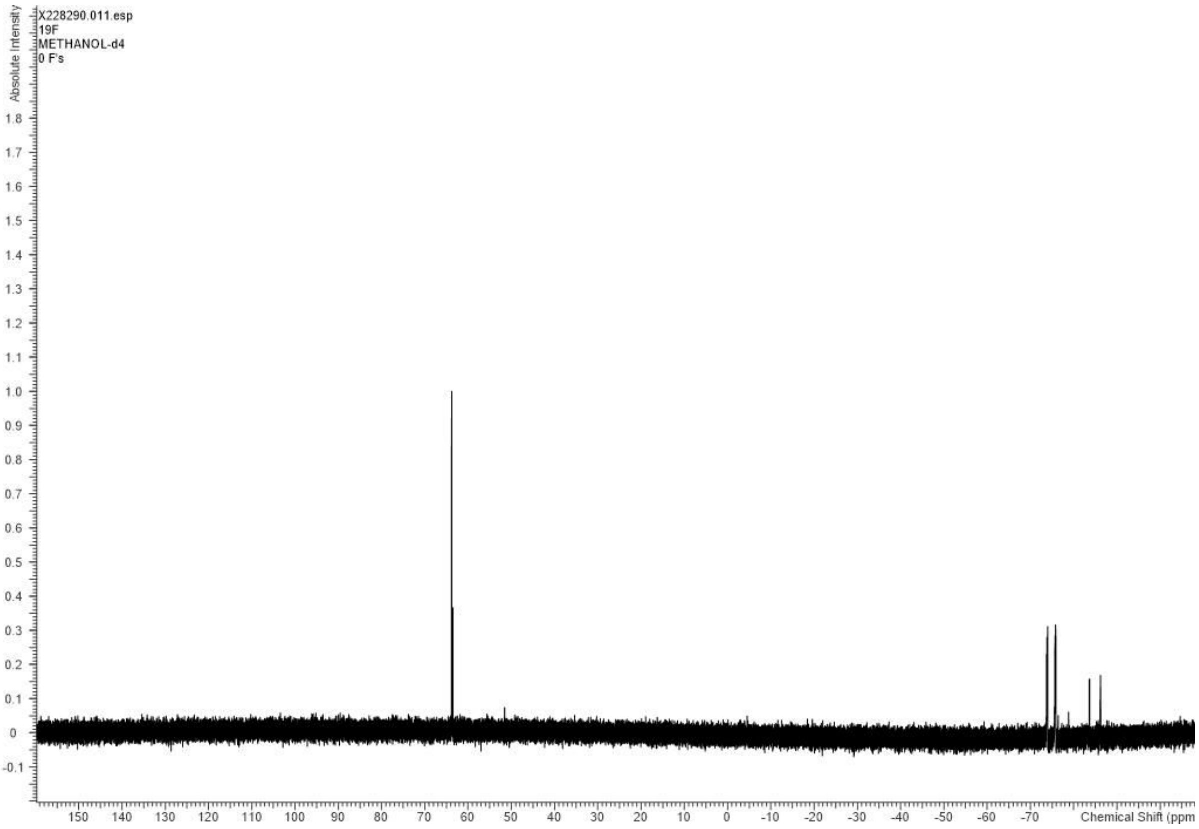
X228290.011.esp
19F
METHANOL-d4
0 F's
Absolute Intensity
1.8
1.7
1.6
1.5
1.4
1.3
1.2
1.1
1.0
0.9
0.8
0.7
0.6
0.5
0.4
0.3
0.2
0.1
0
-0.1
150 140 130 120 110 100 90 80 70 60 50 40 30 20 10 0 -10 -20 -30 -40 -50 -60 -70
Chemical Shift (ppm

5d

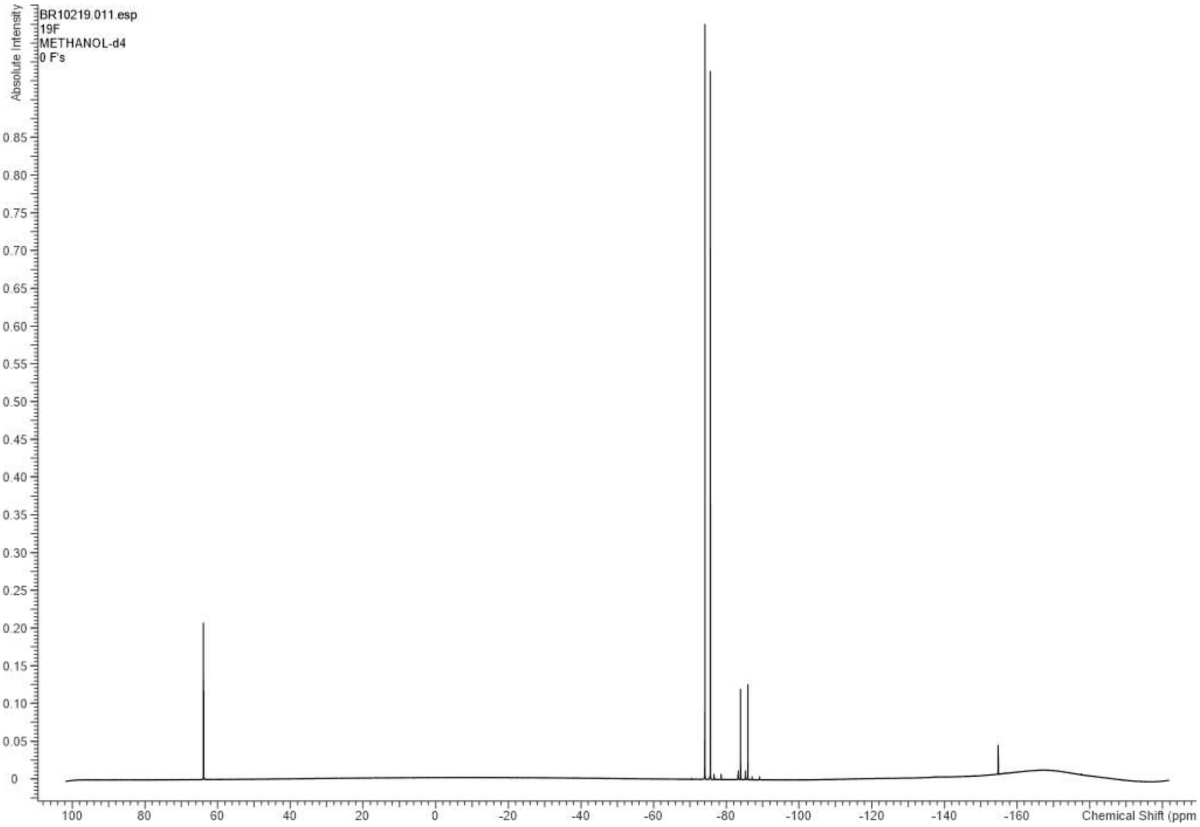
BR10219.011.esp
19F
METHANOL-d4
0 F's
Absolute Intensity
0.85
0.80
0.75
0.70
0.65
0.60
0.55
0.50
0.45
0.40
0.35
0.30
0.25
0.20
0.15
0.10
0.05
0
100 80 60 40 20 0 -20 -40 -60 -80 -100 -120 -140 -160
Chemical Shift (ppm

5e

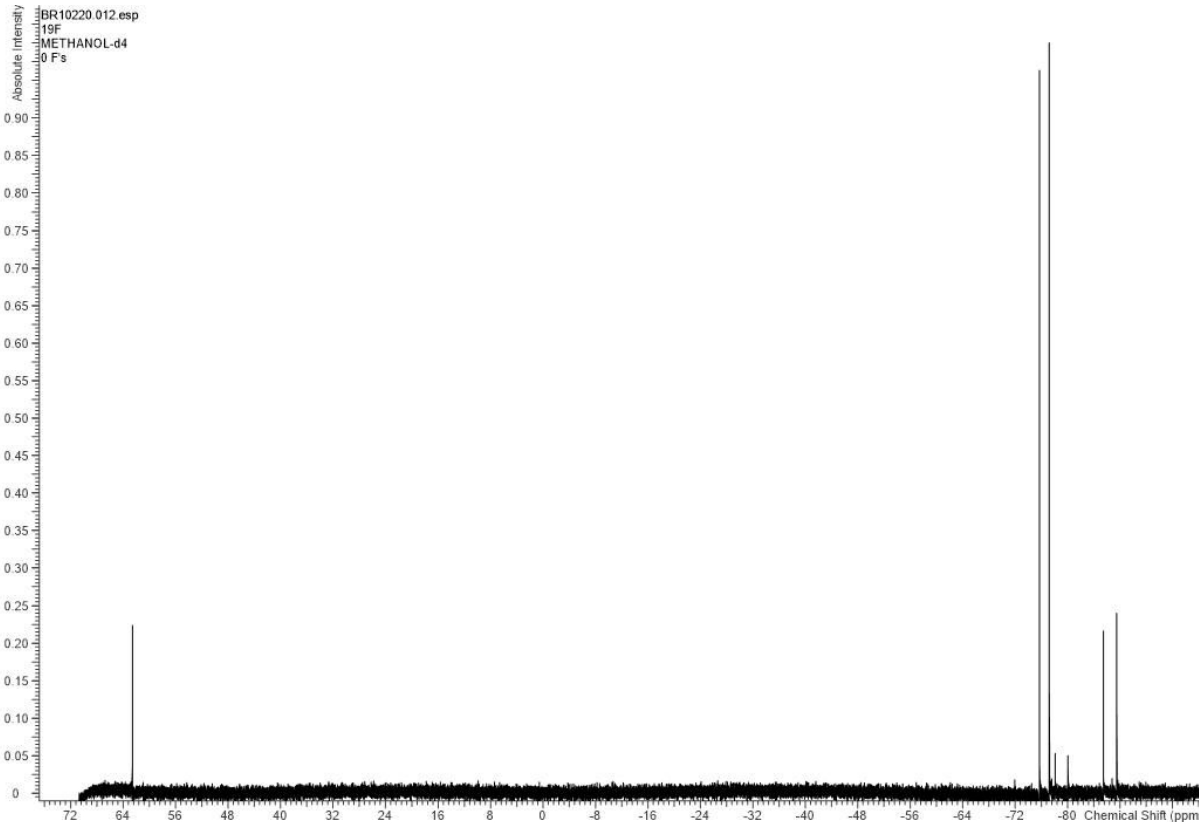
BR10220.012.esp
19F
METHANOL-d4
0 F's
Absolute Intensity
Chemical Shift (ppm

5f

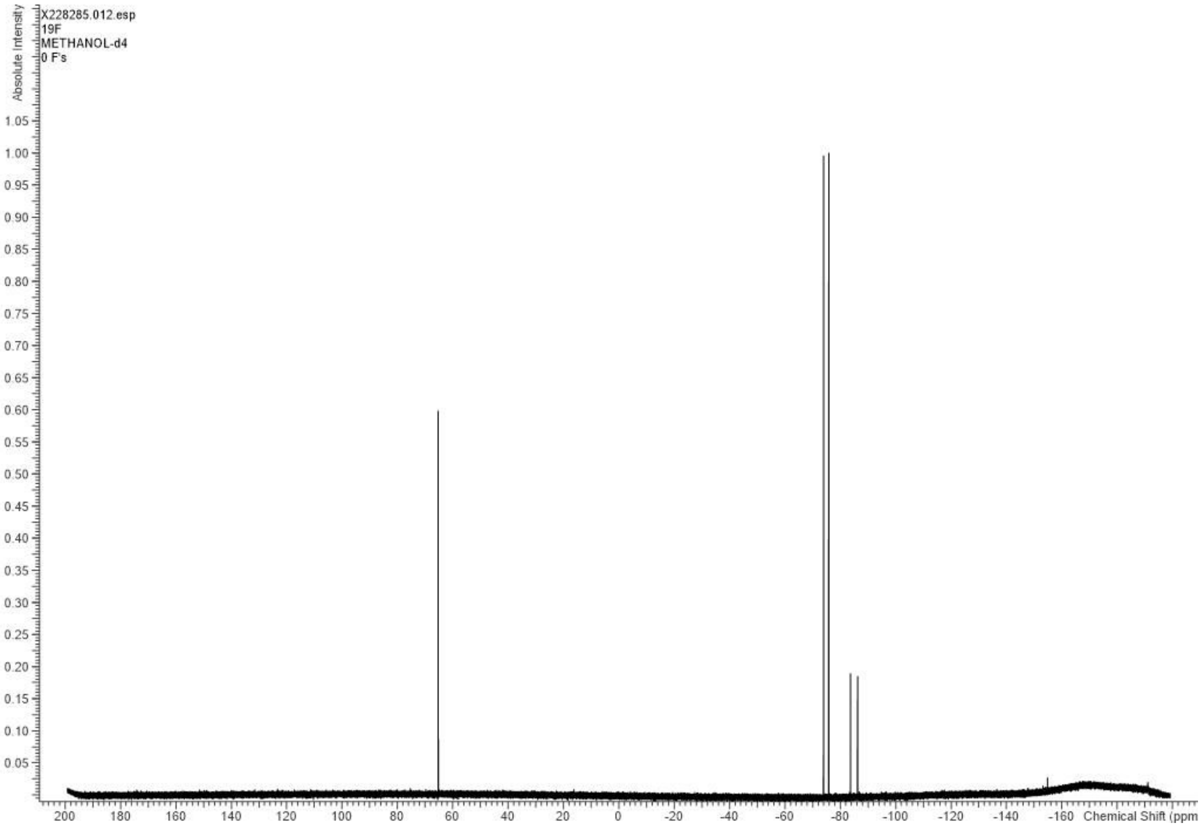
X228285.012.esp
19F
METHANOL-d4
0 F's
Absolute Intensity
Chemical Shift (ppm

5g

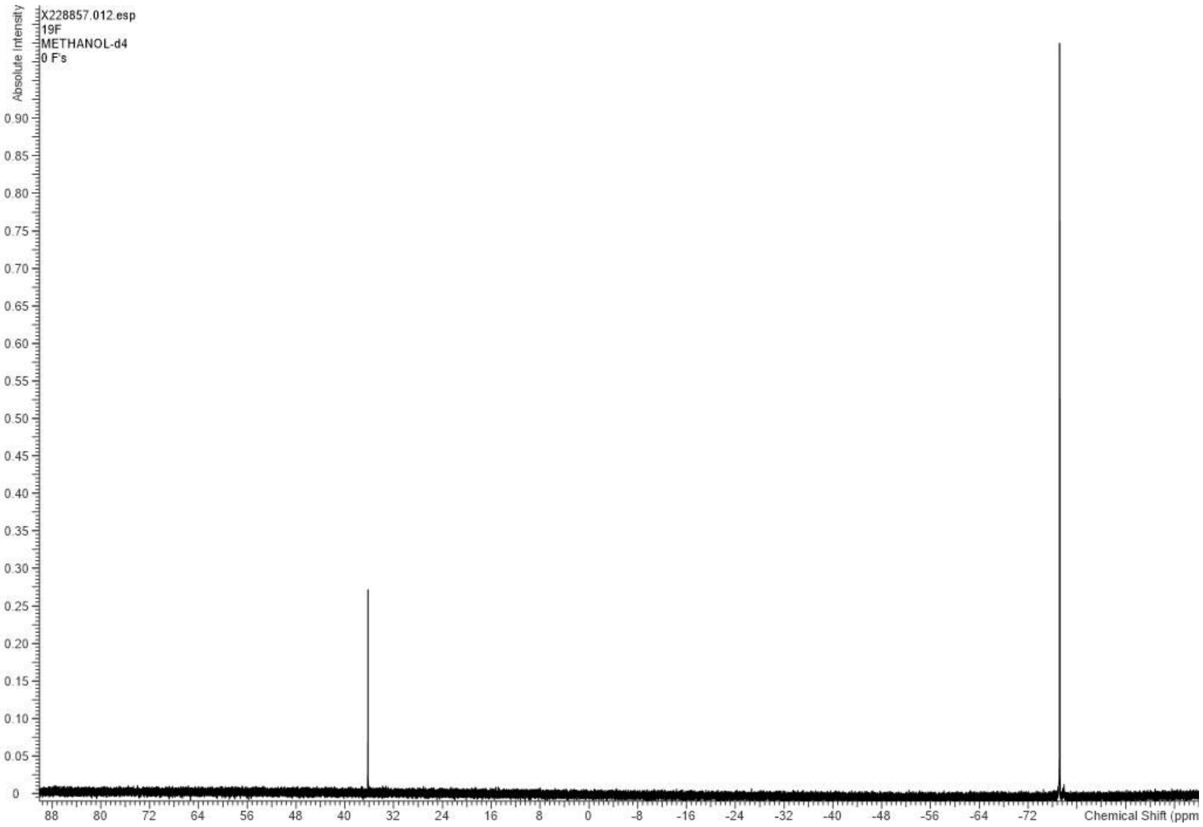
X228857.012.esp
19F
METHANOL-d4
0 F's
Absolute Intensity
0.90
0.85
0.80
0.75
0.70
0.65
0.60
0.55
0.50
0.45
0.40
0.35
0.30
0.25
0.20
0.15
0.10
0.05
0
88
80
72
64
56
48
40
32
24
16
8
0
-8
-16
-24
-32
-40
-48
-56
-64
-72
Chemical Shift (ppm

5h

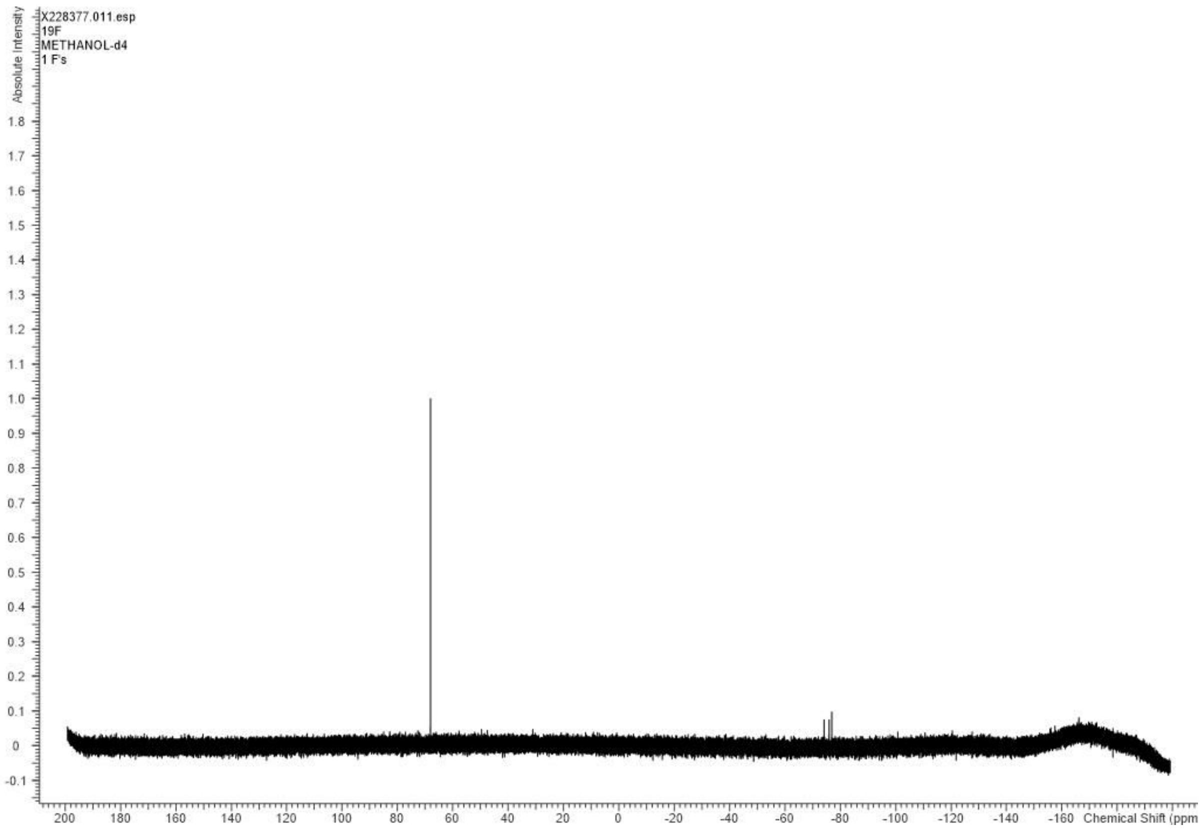
X228377.011.esp
19F
METHANOL-d4
1 F's
Absolute Intensity
1.8
1.7
1.6
1.5
1.4
1.3
1.2
1.1
1.0
0.9
0.8
0.7
0.6
0.5
0.4
0.3
0.2
0.1
0
-0.1
200
180
160
140
120
100
80
60
40
20
0
-20
-40
-60
-80
-100
-120
-140
-160
Chemical Shift (ppm

5i

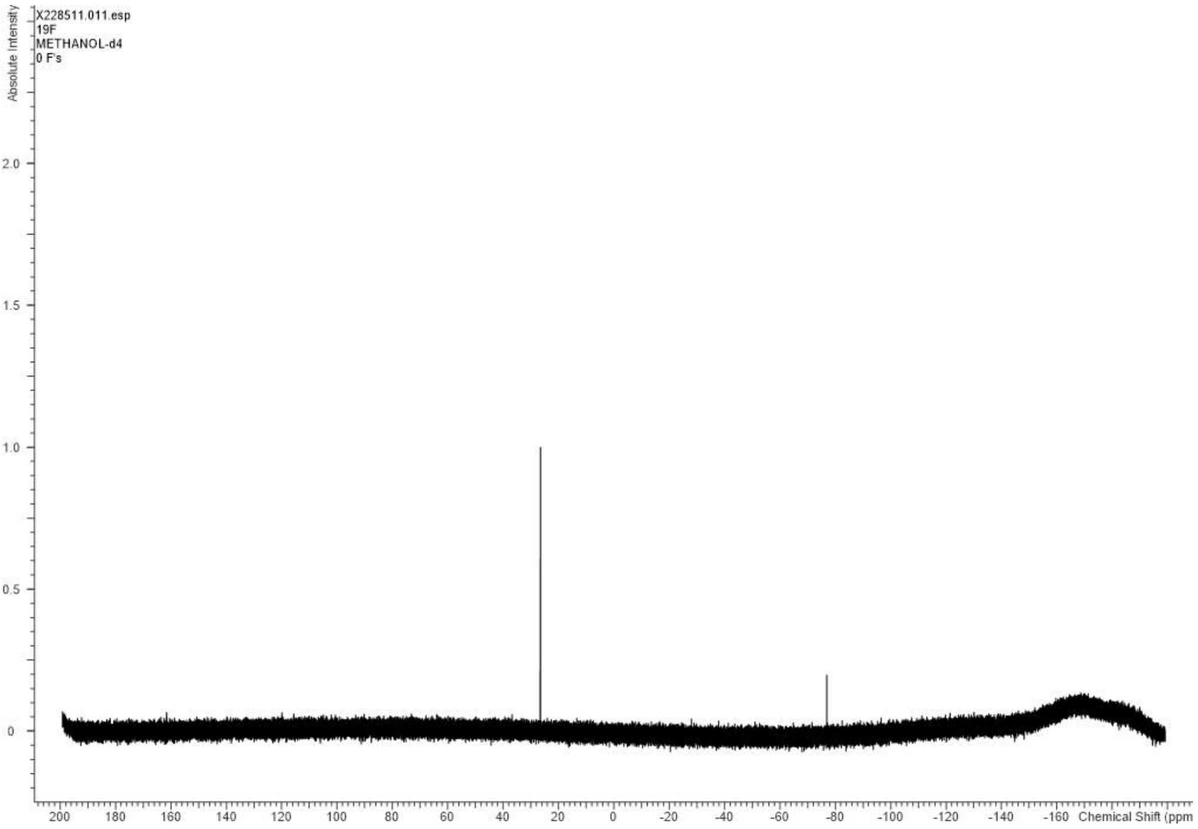
X228511.011.esp
19F
METHANOL-d4
0 F's
Absolute Intensity
2.0
1.5
1.0
0.5
0
200 180 160 140 120 100 80 60 40 20 0 -20 -40 -60 -80 -100 -120 -140 -160 Chemical Shift (ppm

11. ^{13}C NMR spectra

1a

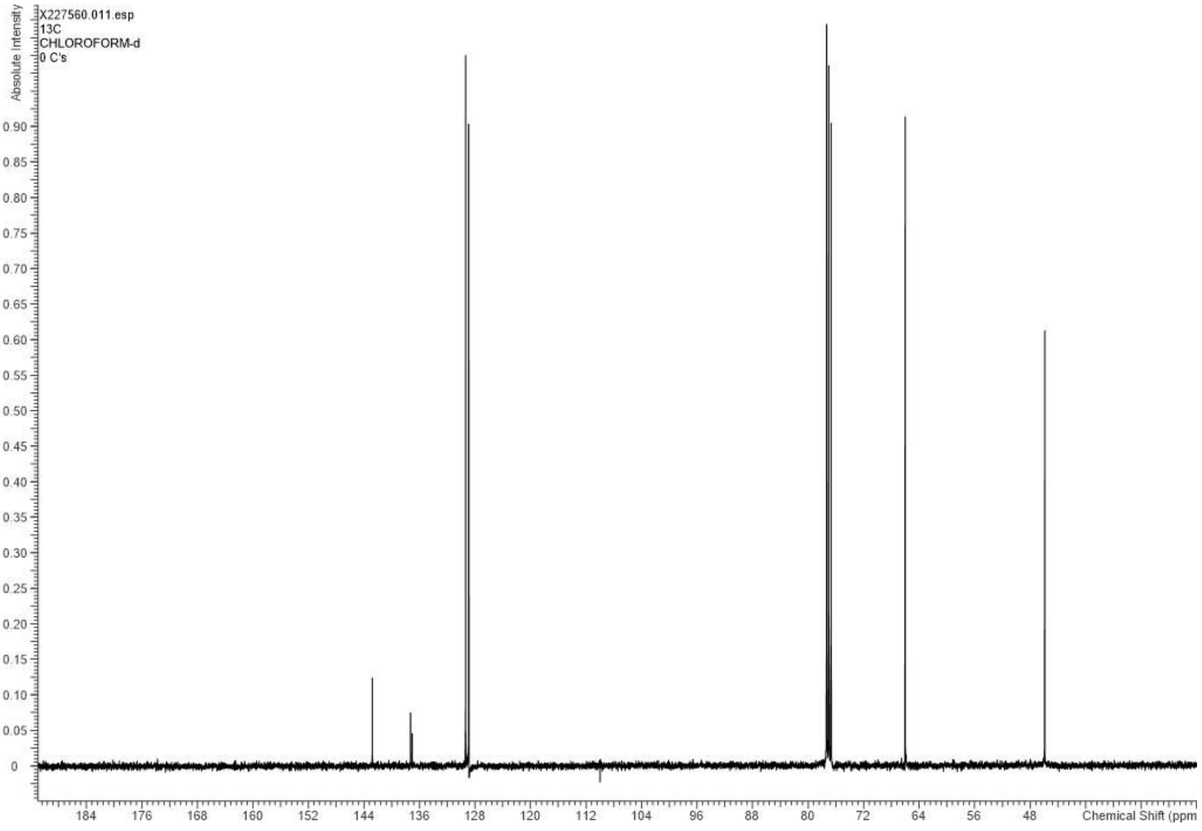

1b

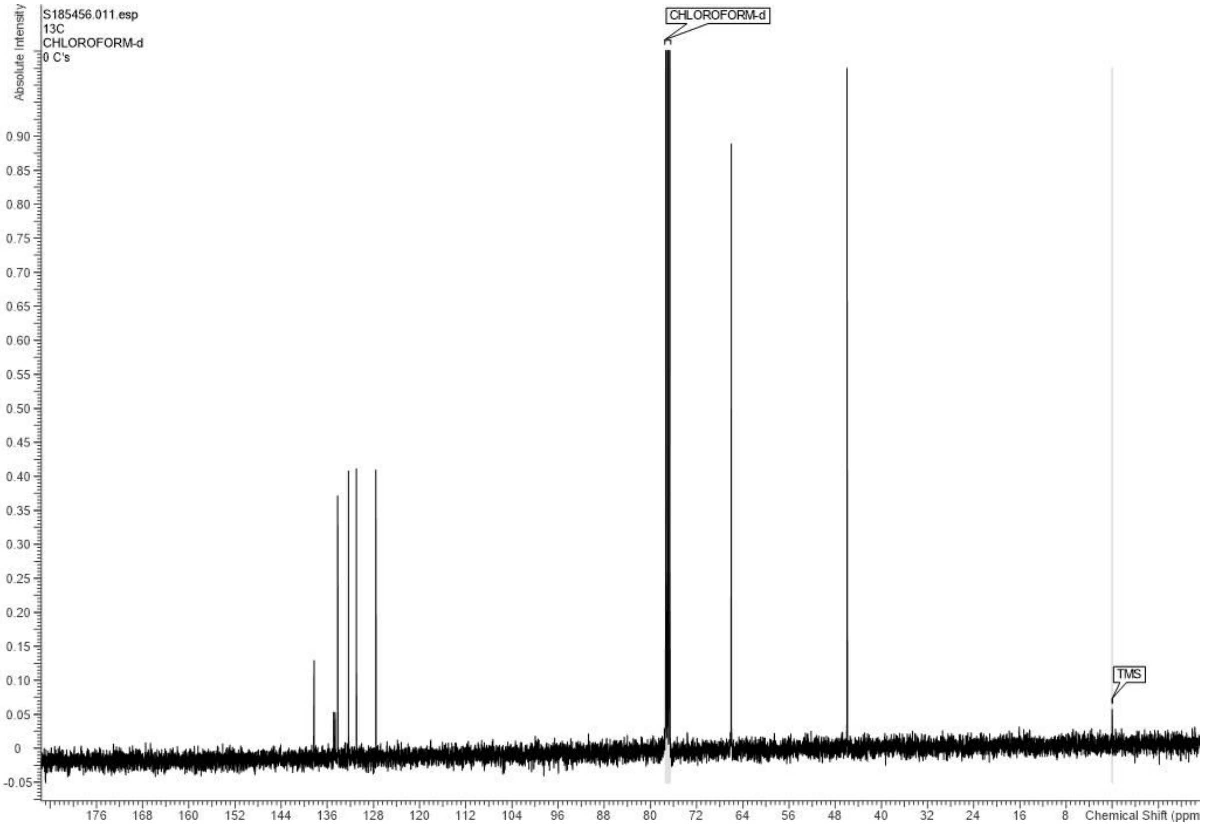

1c

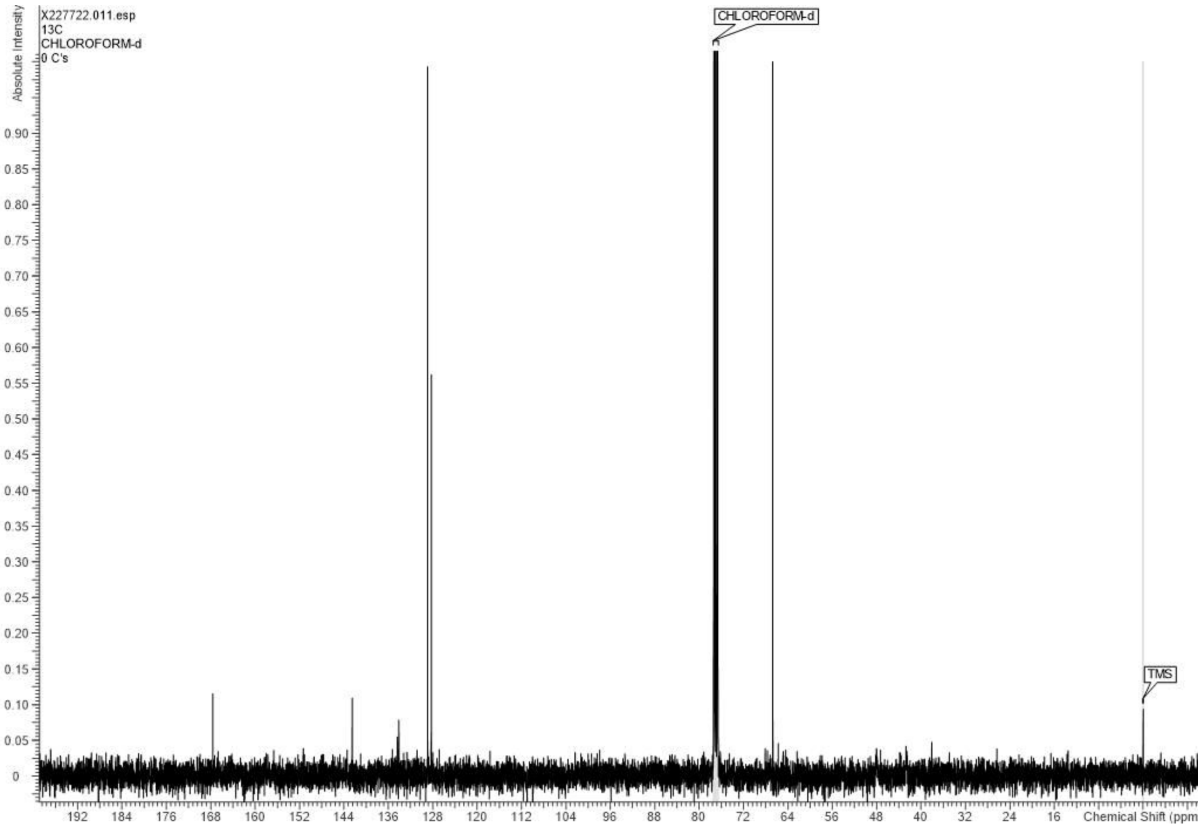
X227722.011.esp
13C
CHLOROFORM-d
0 C's
CHLOROFORM-d
TMS
Absolute Intensity
Chemical Shift (ppm

1d

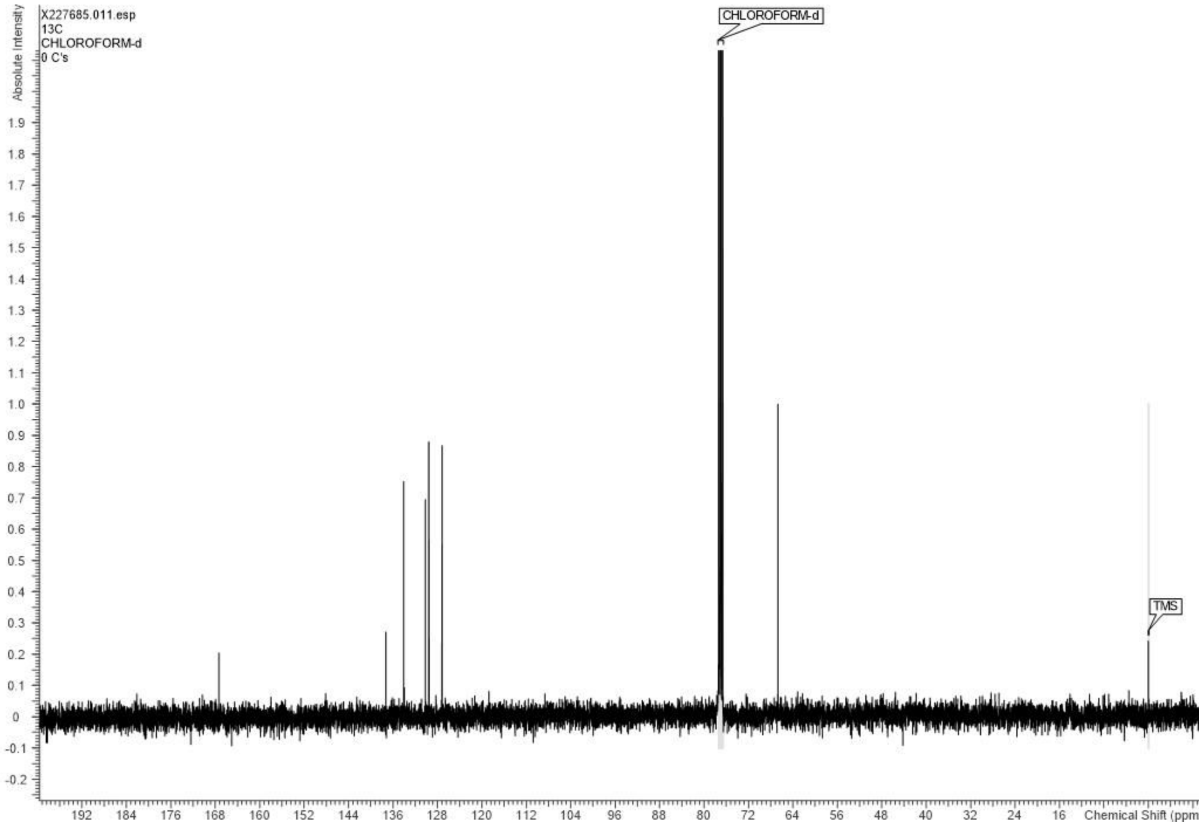
X227685.011.esp
13C
CHLOROFORM-d
0 C's
CHLOROFORM-d
TMS
Absolute Intensity
Chemical Shift (ppm

1e

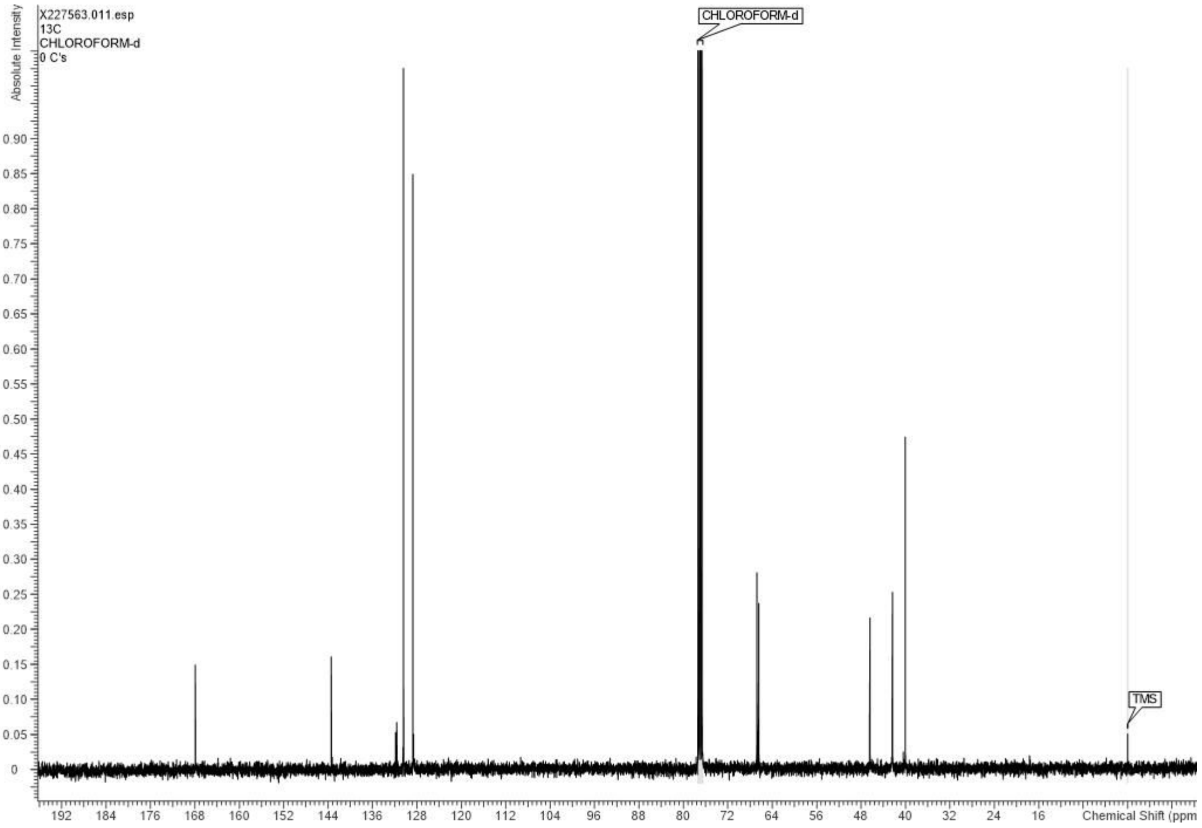
X227563.011.esp
13C
CHLOROFORM-d
0 C's
CHLOROFORM-d
TMS
Absolute Intensity
Chemical Shift (ppm

1f

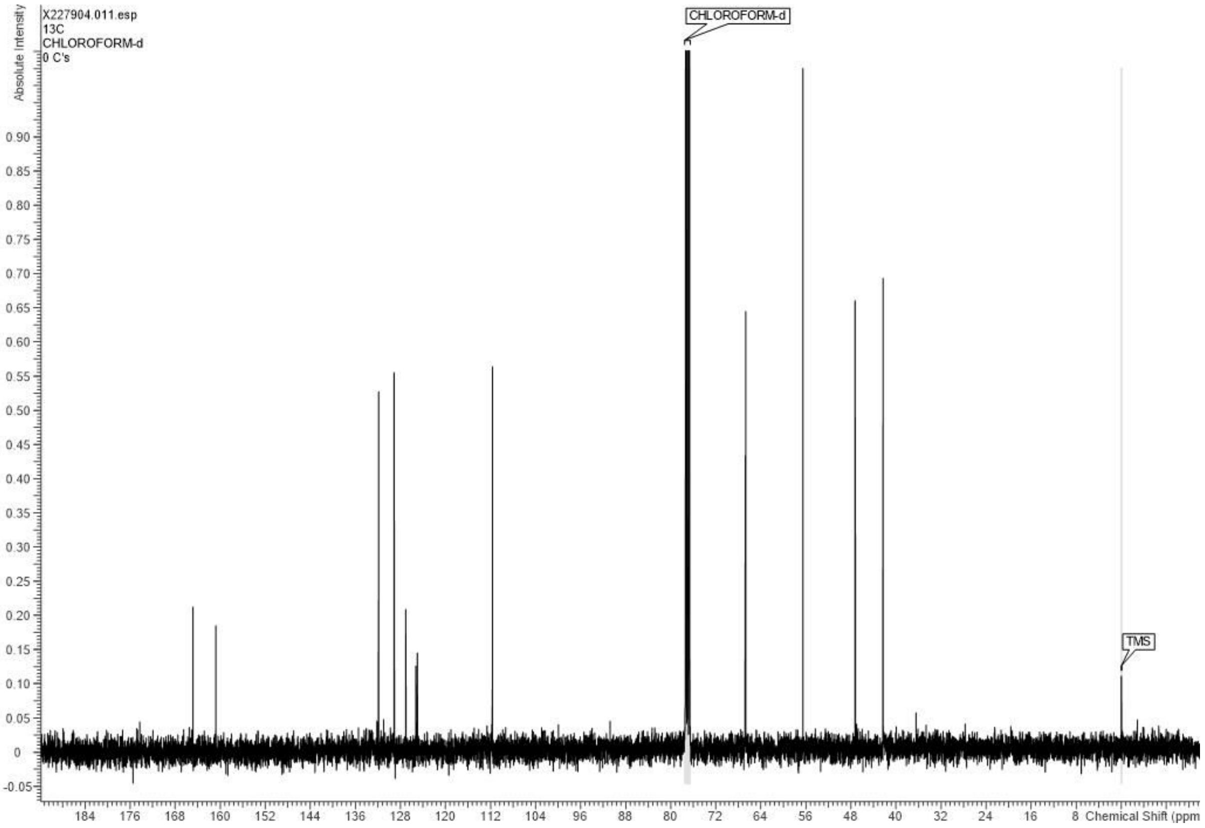
X227904.011.esp
13C
CHLOROFORM-d
0 C's
CHLOROFORM-d
TMS
Absolute Intensity
Chemical Shift (ppm

1g

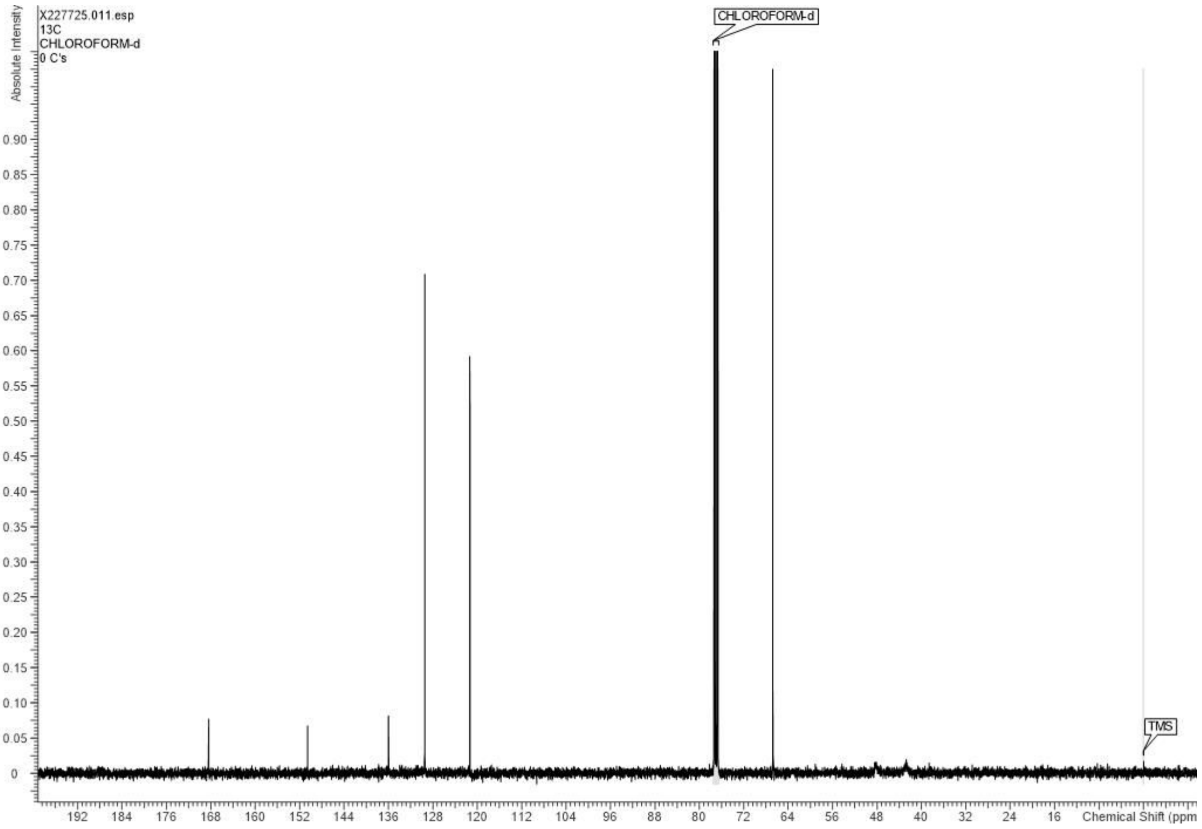
X227725.011.esp
13C
CHLOROFORM-d
0 C's
CHLOROFORM-d
TMS
Absolute Intensity
Chemical Shift (ppm

1h

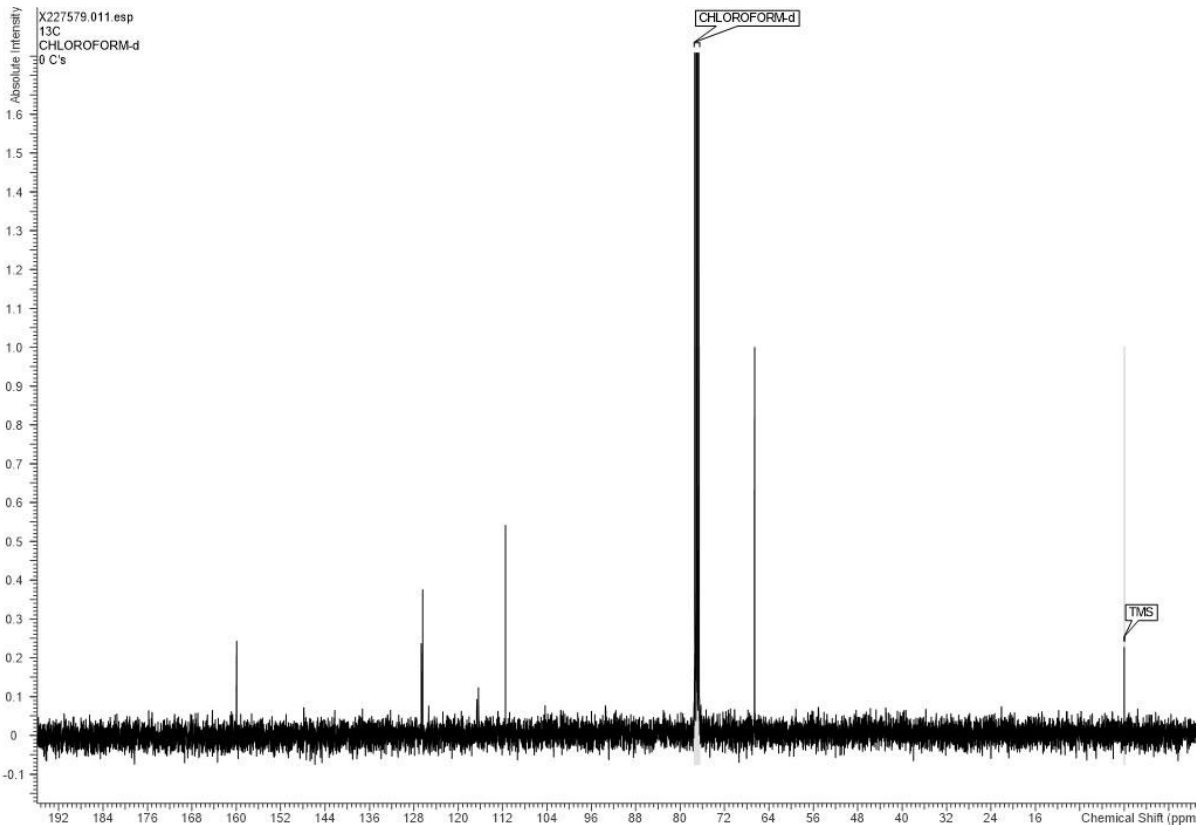
X227579.011.esp
13C
CHLOROFORM-d
0 C's
CHLOROFORM-d
TMS
Absolute Intensity
Chemical Shift (ppm

1i

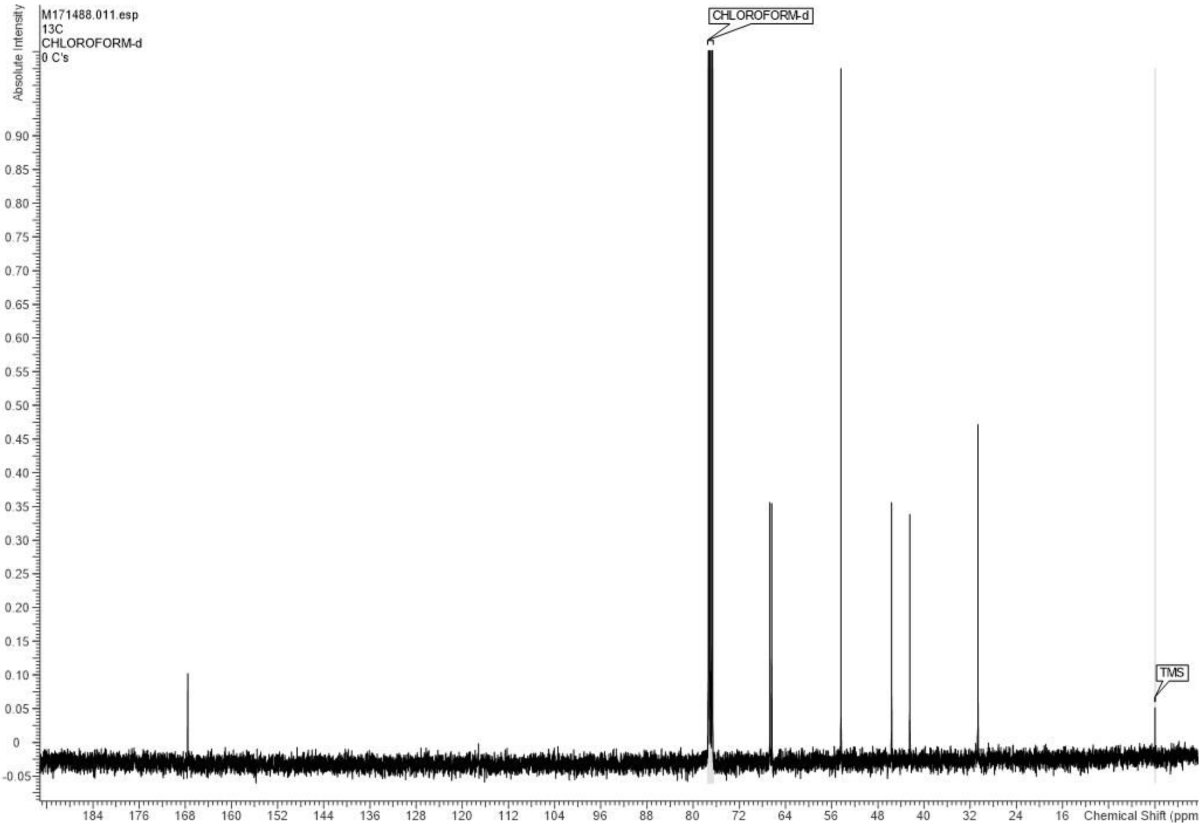

M171488.011.esp
13C
CHLOROFORM-d
0 C's
CHLOROFORM-d
TMS
Absolute Intensity
Chemical Shift (ppm)

2a

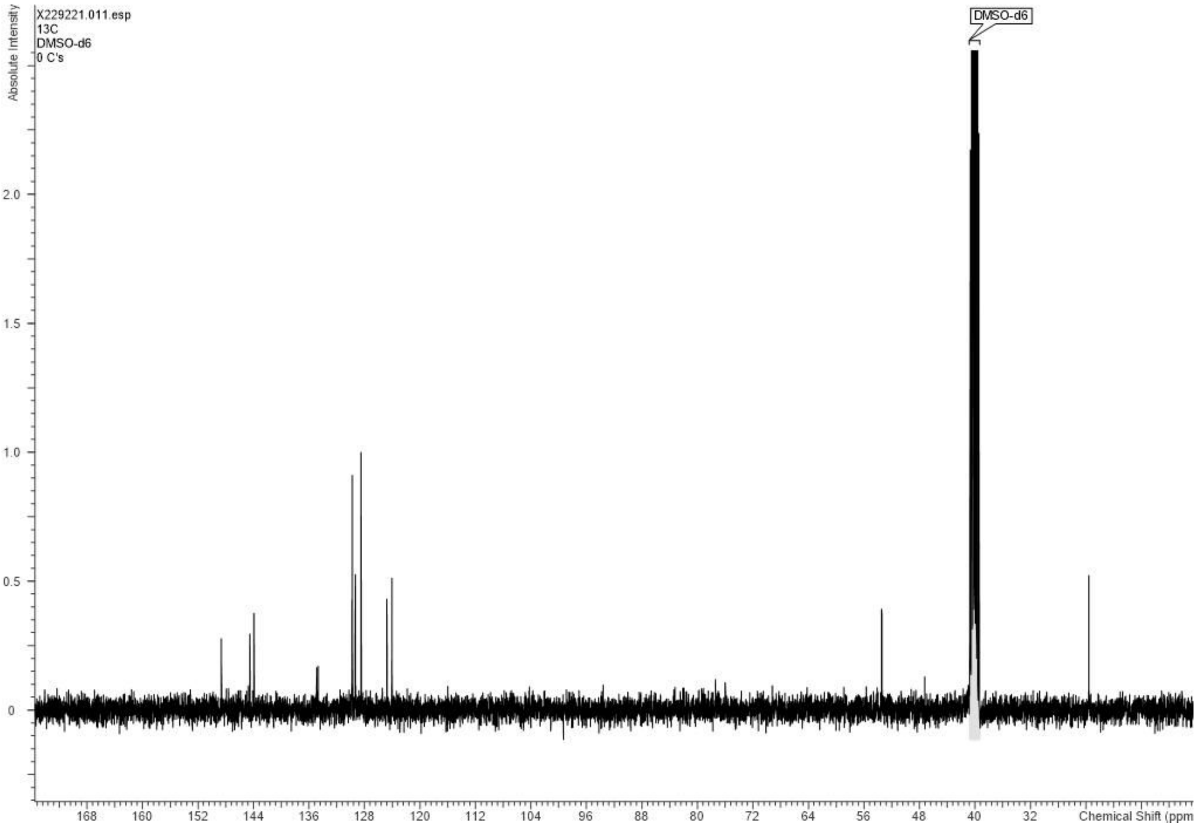

X229221.011.esp
13C
DMSO-d6
0 C's
DMSO-d6
Absolute Intensity
Chemical Shift (ppm)

2b

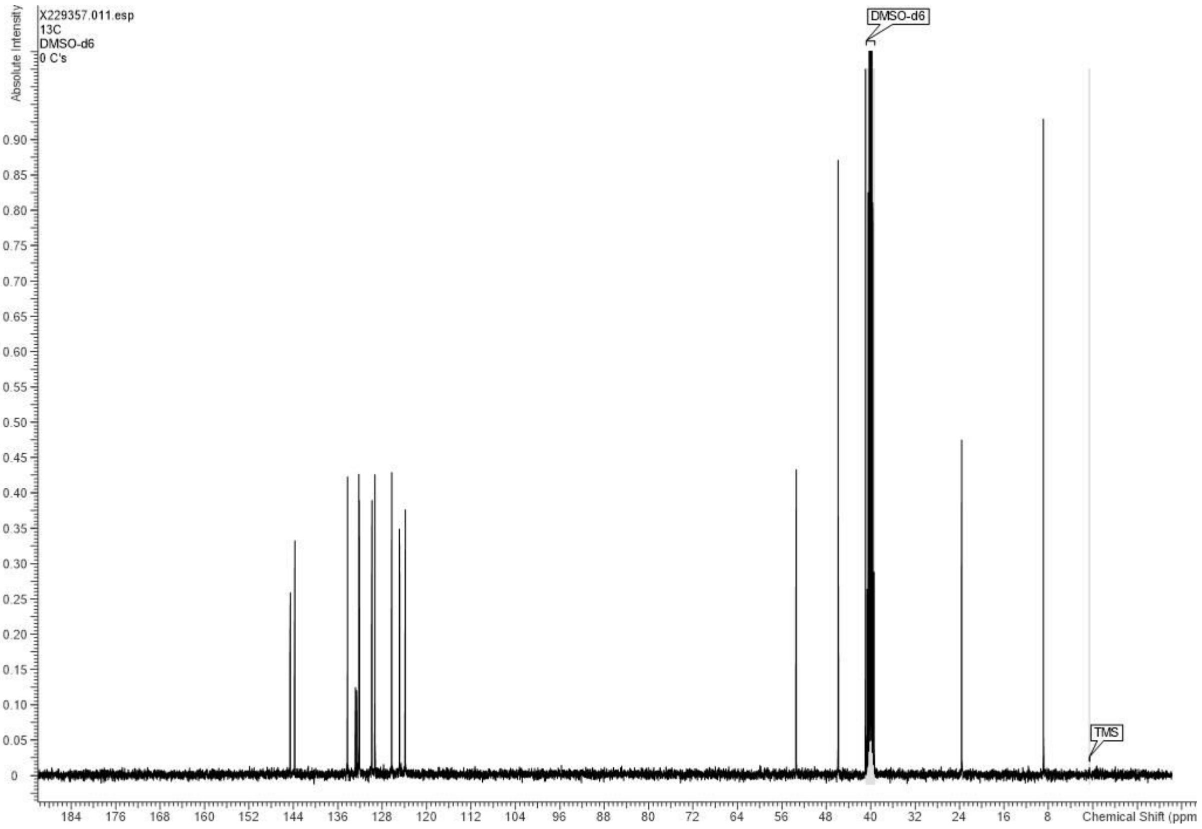

X229357.011.esp
13C
DMSO-d6
0 C's
DMSO-d6
TMS
Absolute Intensity
Chemical Shift (ppm)

2c

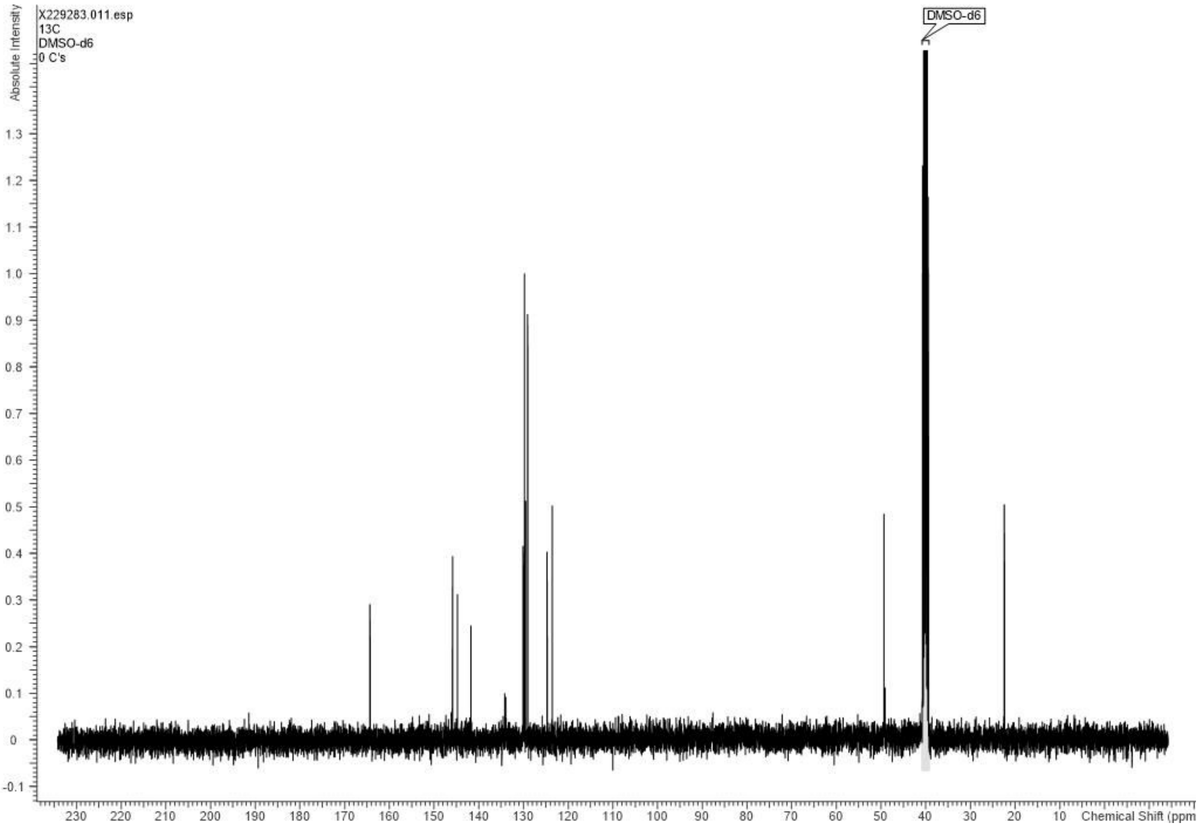

X229283.011.esp
13C
DMSO-d6
0 C's
DMSO-d6
Absolute Intensity
Chemical Shift (ppm)

2d

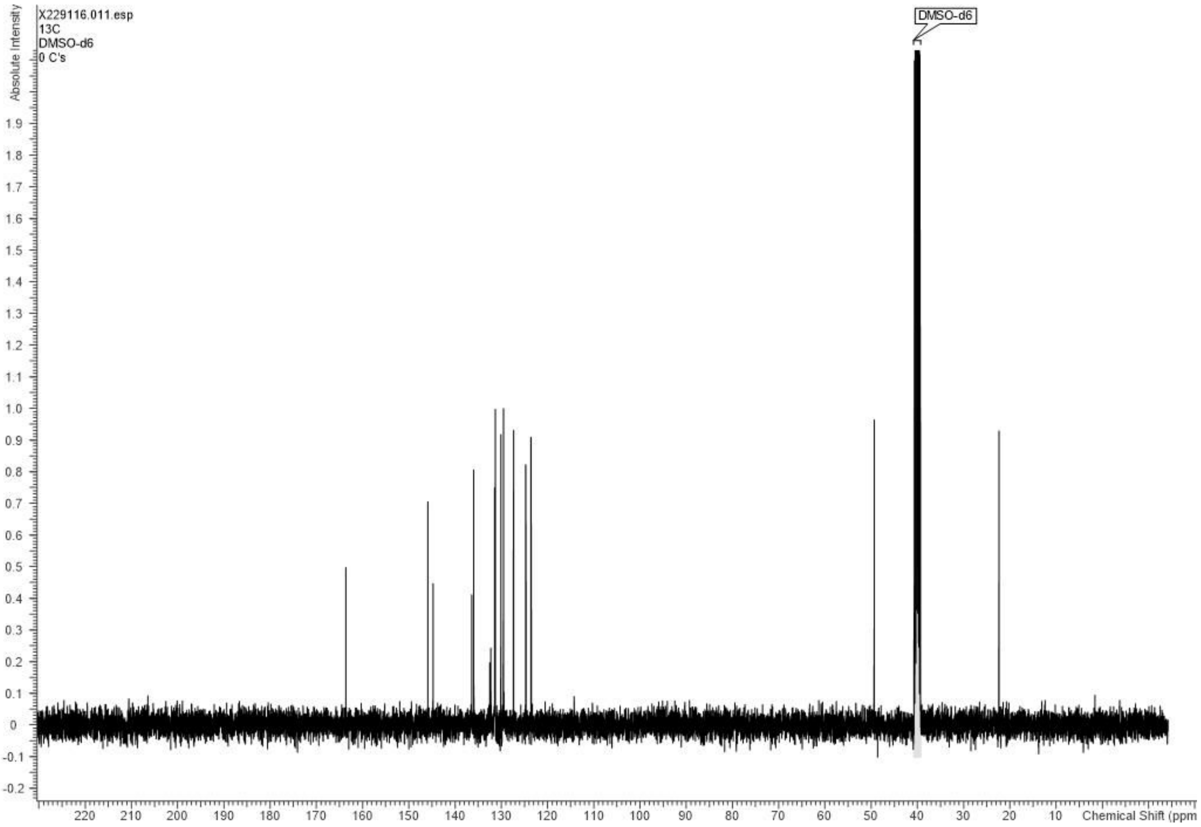
X229116.011.esp
13C
DMSO-d6
0 C's
DMSO-d6
Absolute Intensity
Chemical Shift (ppm)

2e

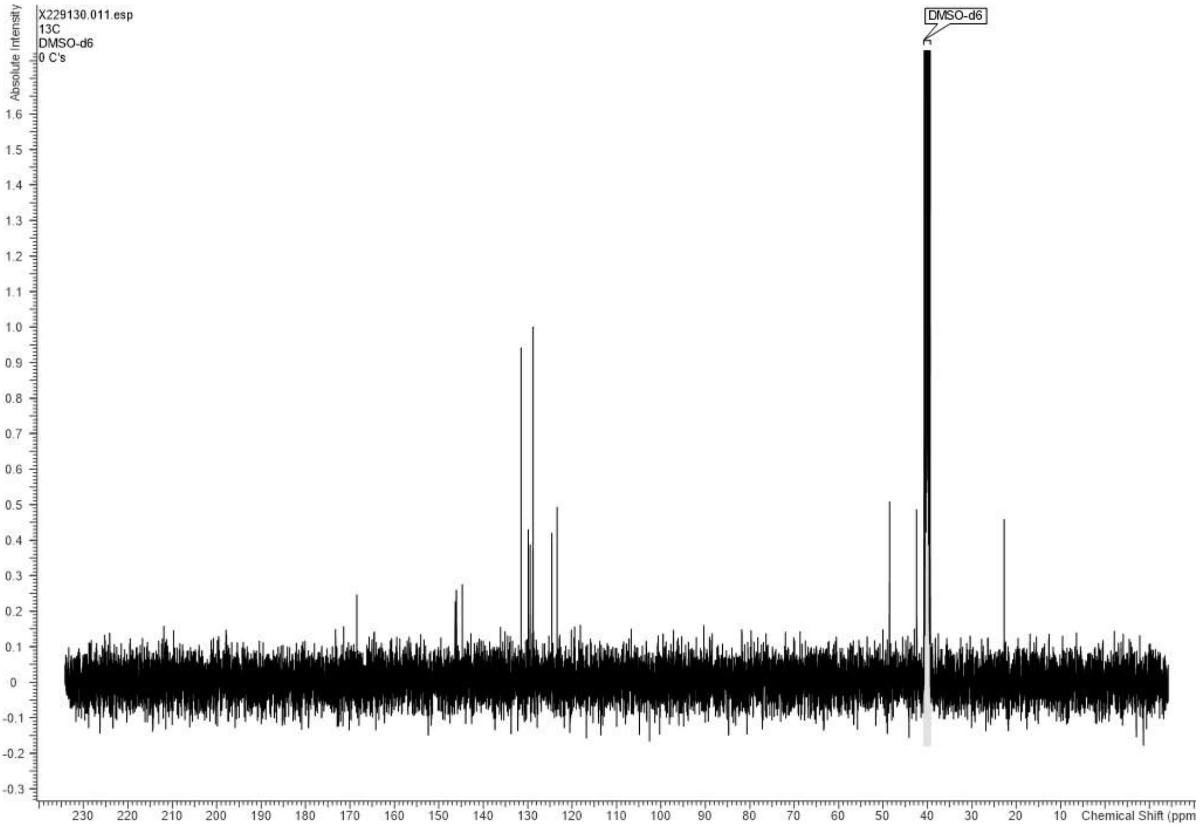
X229130.011.esp
13C
DMSO-d6
0 C's
DMSO-d6
Absolute Intensity
Chemical Shift (ppm)

2f

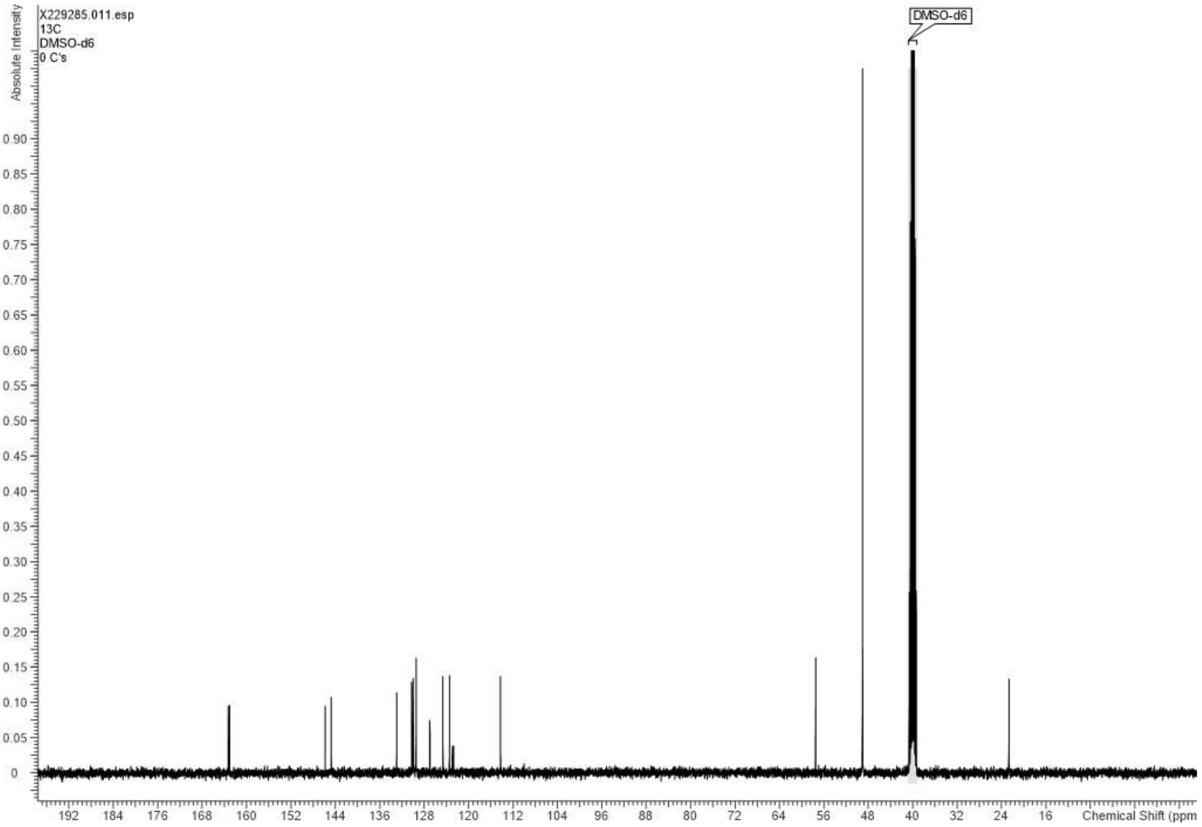
X229285.011.esp
13C
DMSO-d6
0 C's
Absolute Intensity
DMSO-d6
0.90
0.85
0.80
0.75
0.70
0.65
0.60
0.55
0.50
0.45
0.40
0.35
0.30
0.25
0.20
0.15
0.10
0.05
0
192 184 176 168 160 152 144 136 128 120 112 104 96 88 80 72 64 56 48 40 32 24 16
Chemical Shift (ppm

2g

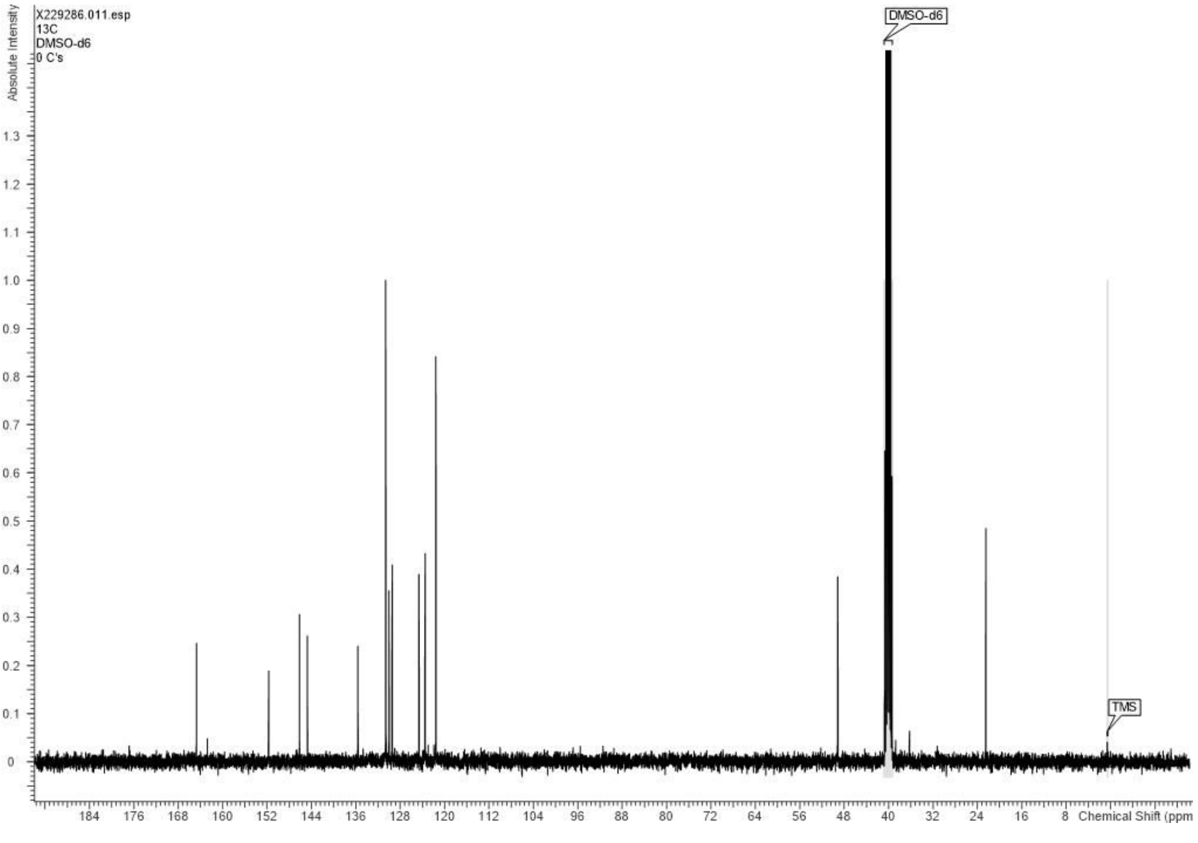
X229286.011.esp
13C
DMSO-d6
0 C's
Absolute Intensity
DMSO-d6
TMS
1.3
1.2
1.1
1.0
0.9
0.8
0.7
0.6
0.5
0.4
0.3
0.2
0.1
0
184 176 168 160 152 144 136 128 120 112 104 96 88 80 72 64 56 48 40 32 24 16 8
Chemical Shift (ppm

2h

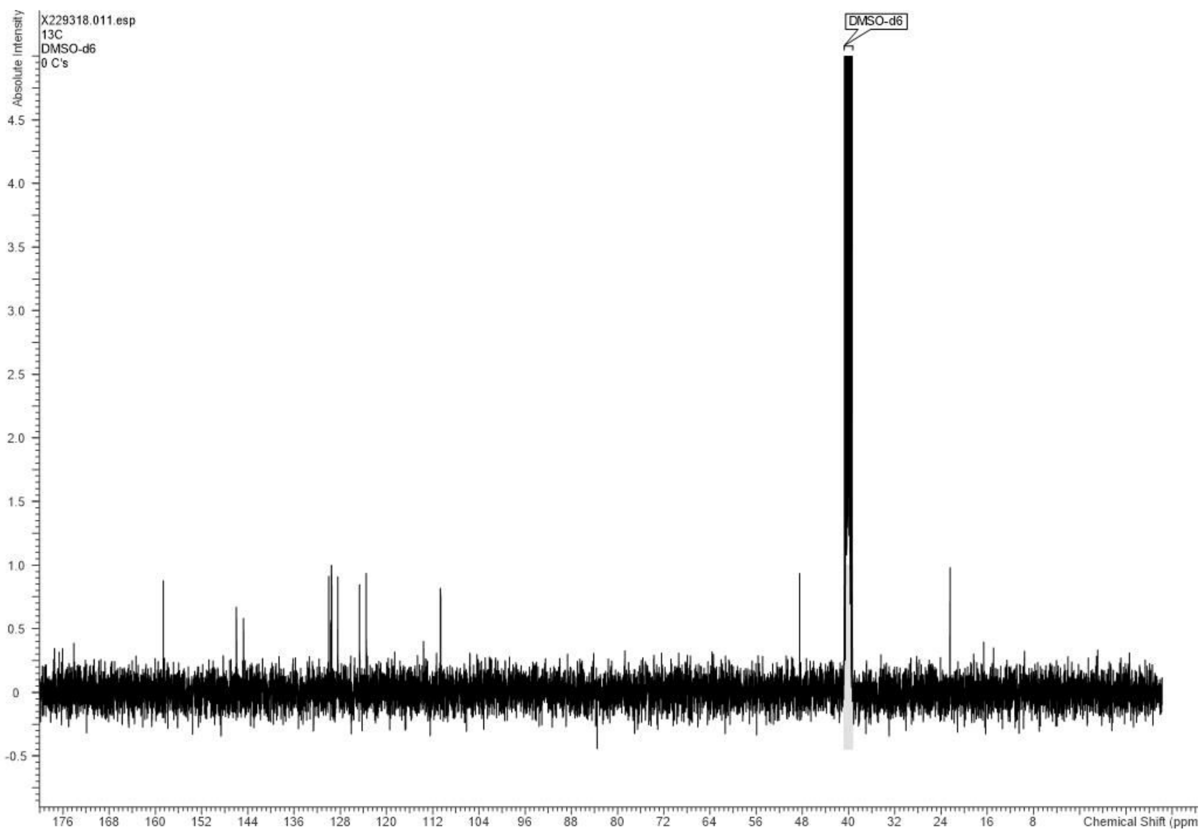
X229318.011.esp
13C
DMSO-d6
0 C's
DMSO-d6
Absolute Intensity
Chemical Shift (ppm)

2i

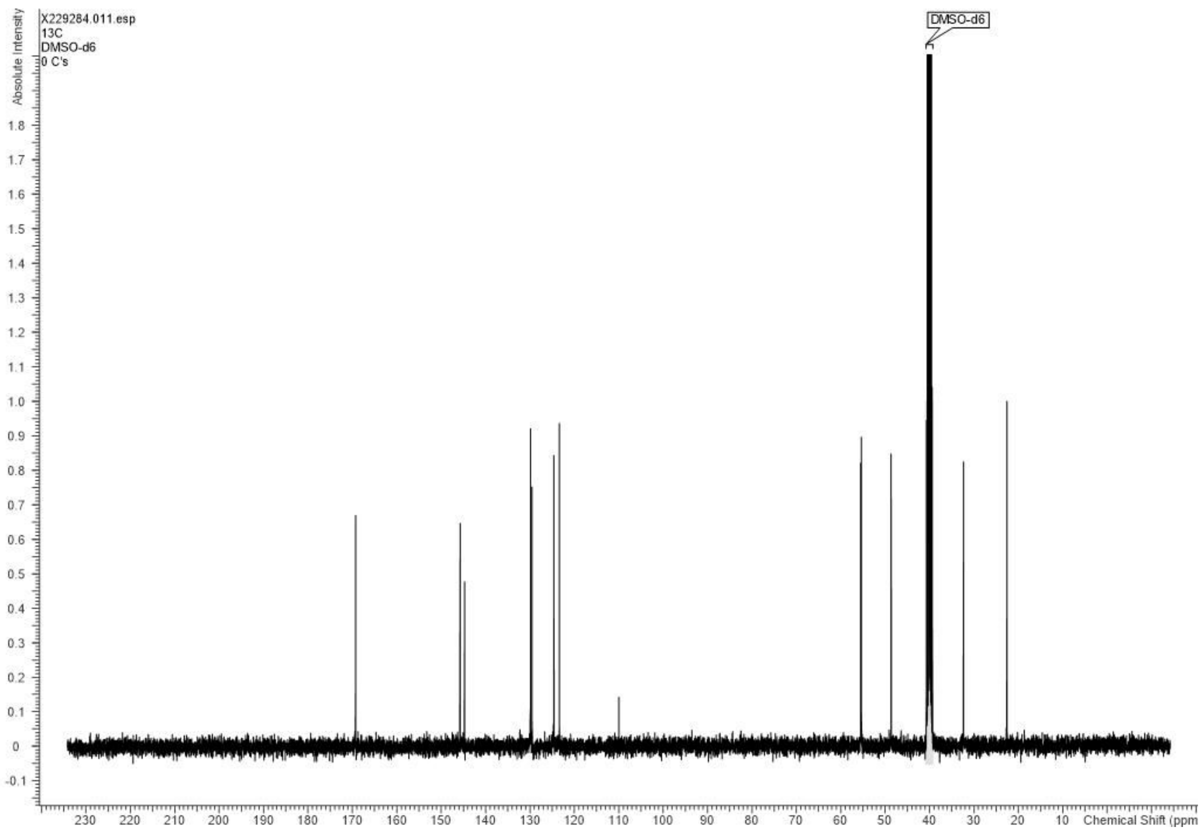
X229284.011.esp
13C
DMSO-d6
0 C's
DMSO-d6
Absolute Intensity
Chemical Shift (ppm)

5a

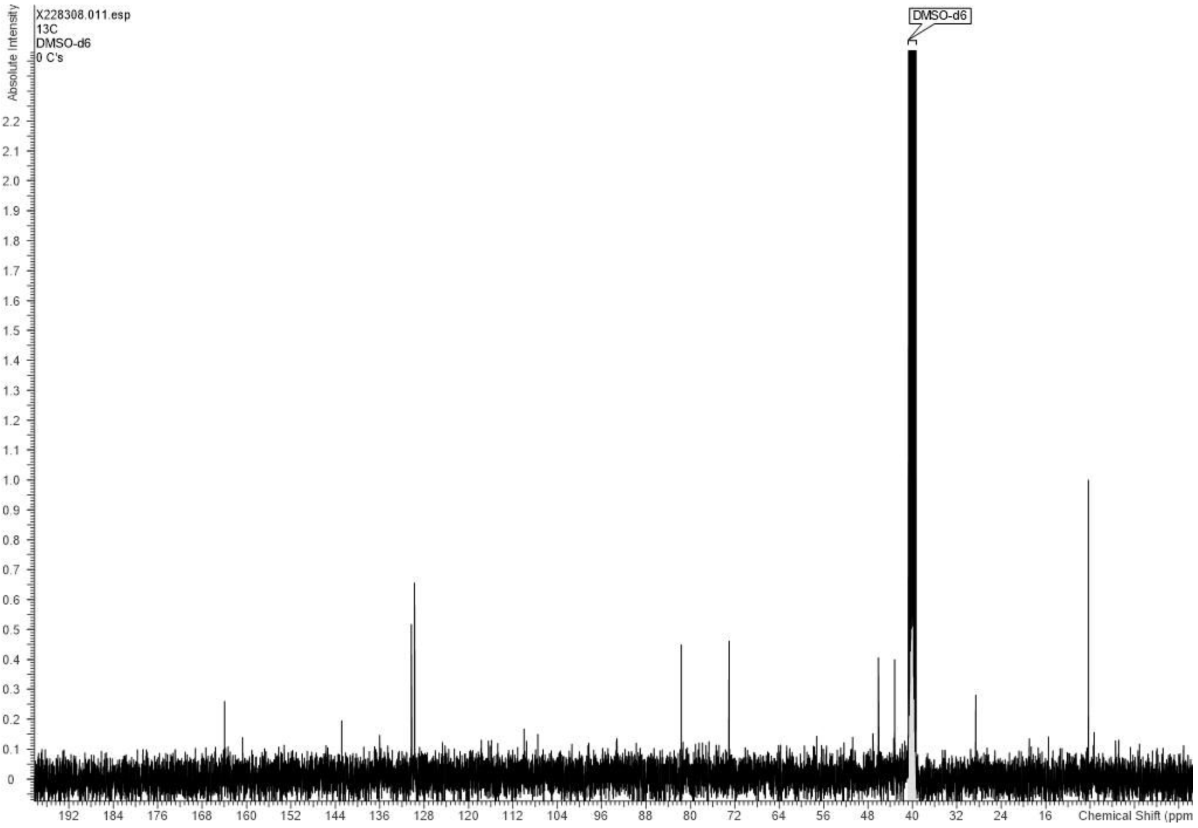
X228308.011.esp
13C
DMSO-d6
0 C's
DMSO-d6
Absolute Intensity
Chemical Shift (ppm

5b

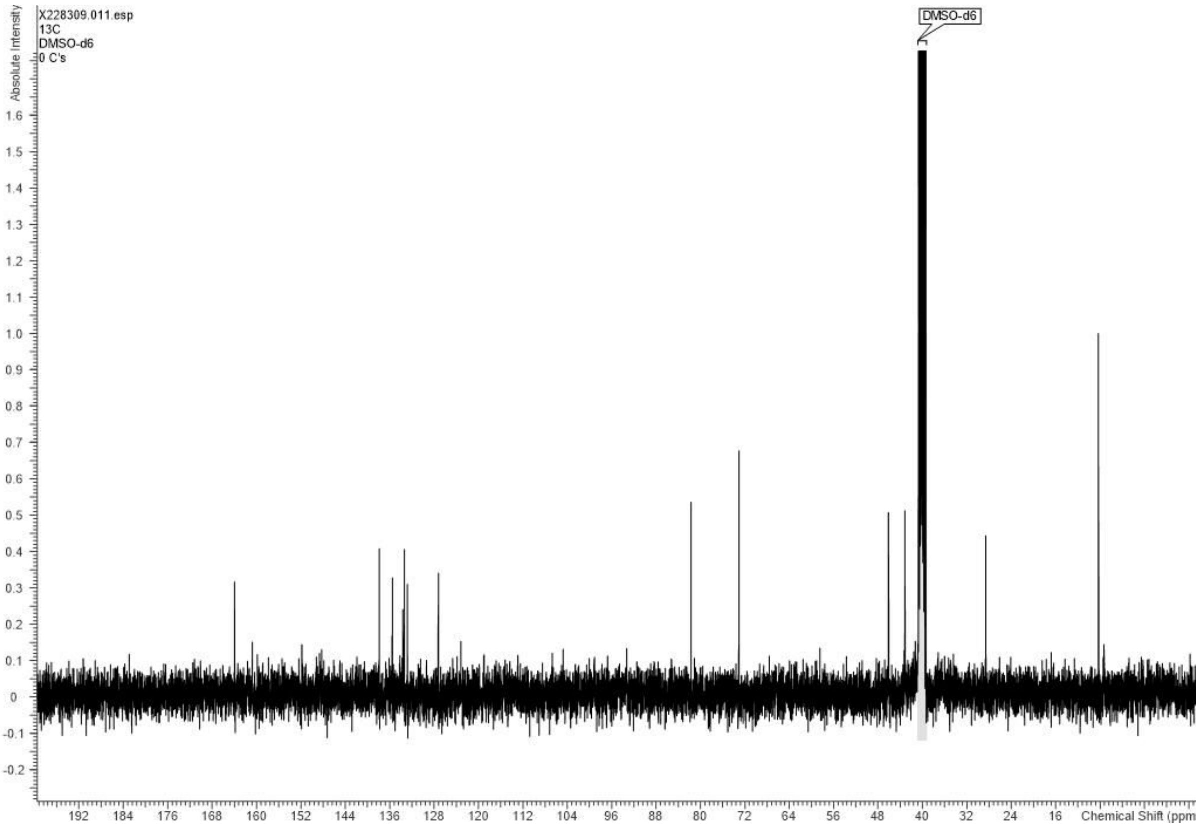
X228309.011.esp
13C
DMSO-d6
0 C's
DMSO-d6
Absolute Intensity
Chemical Shift (ppm

5c

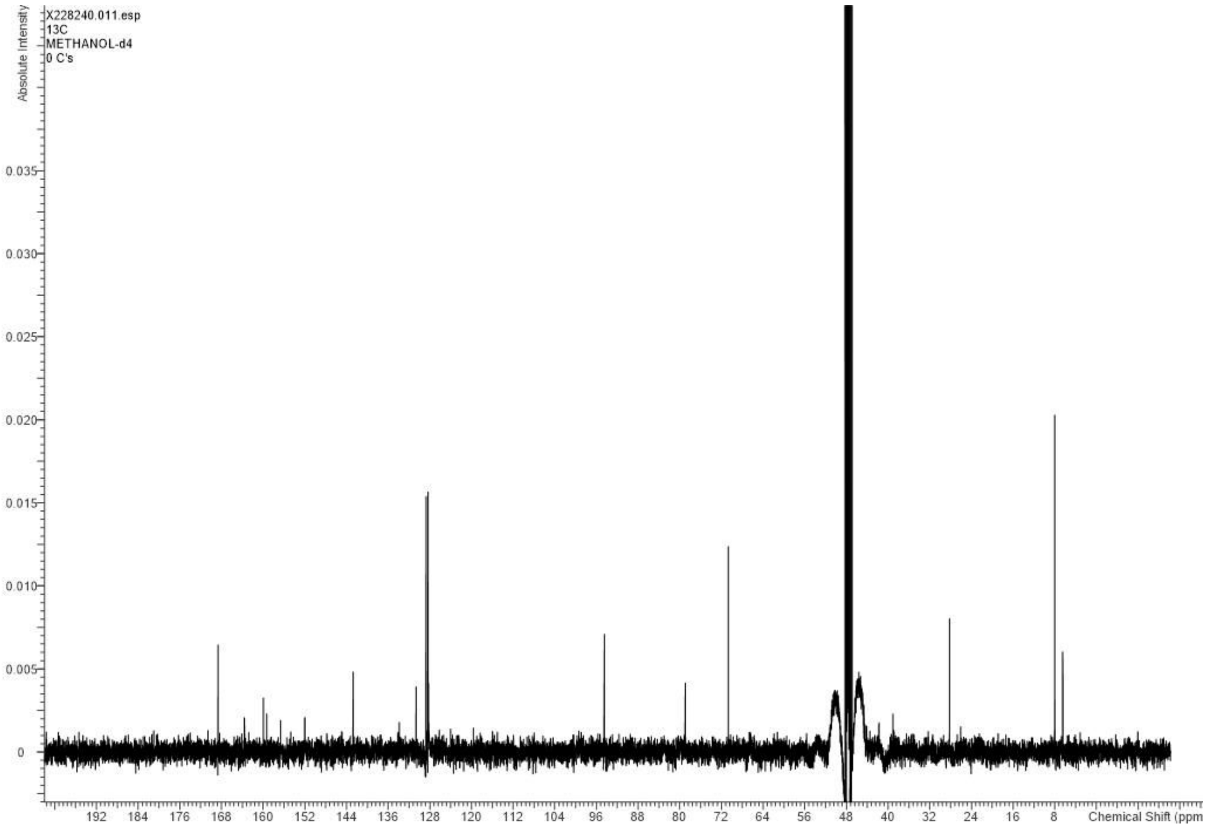

X228240.011.esp
13C
METHANOL-d4
0 C's
Absolute Intensity
Chemical Shift (ppm)

5d

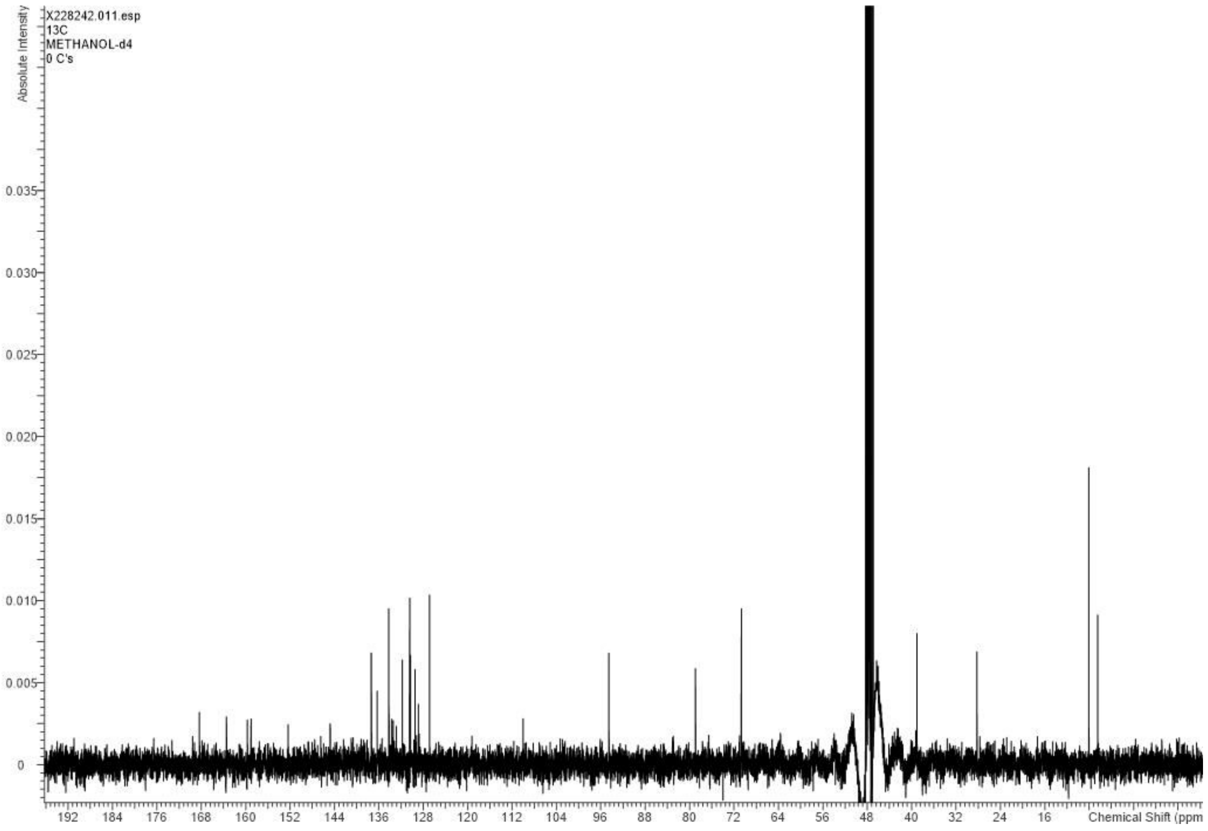

X228242.011.esp
13C
METHANOL-d4
0 C's
Absolute Intensity
Chemical Shift (ppm)

5e

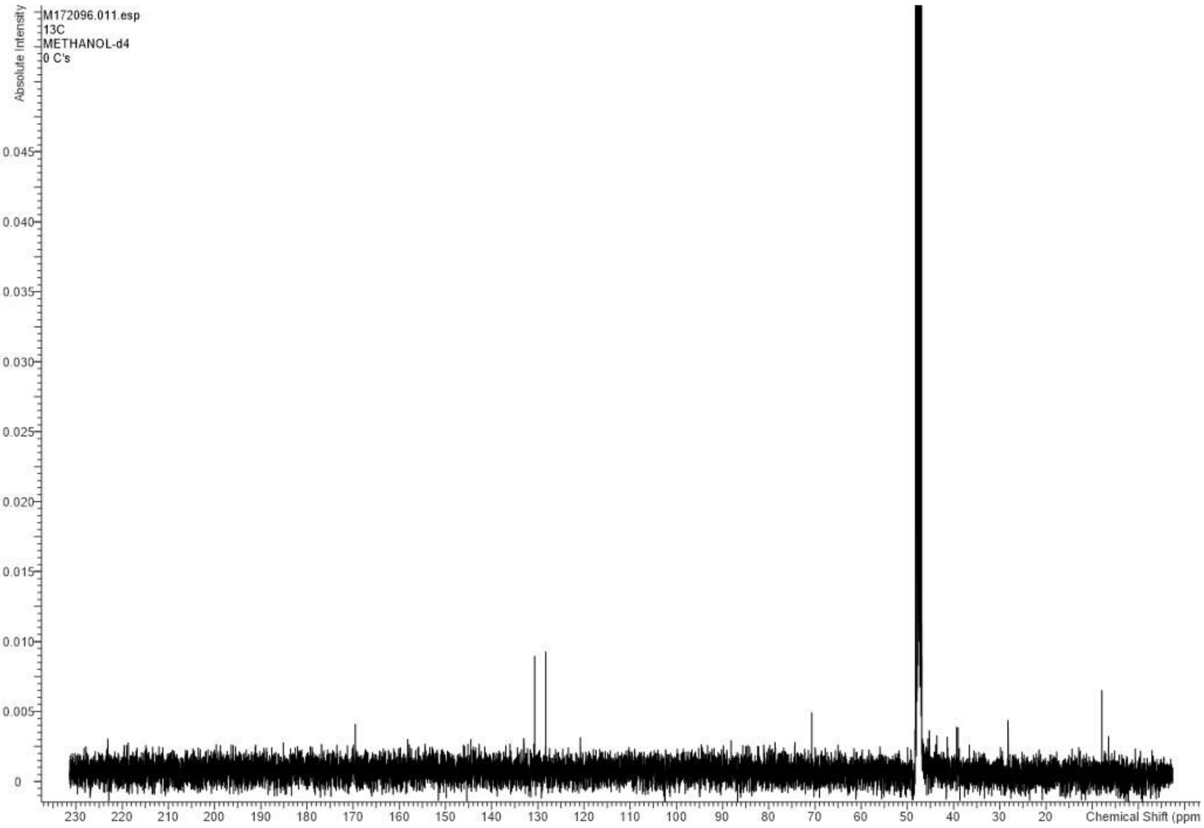

M172096.011.esp
13C
METHANOL-d4
0 C's
Absolute Intensity
Chemical Shift (ppm

5f

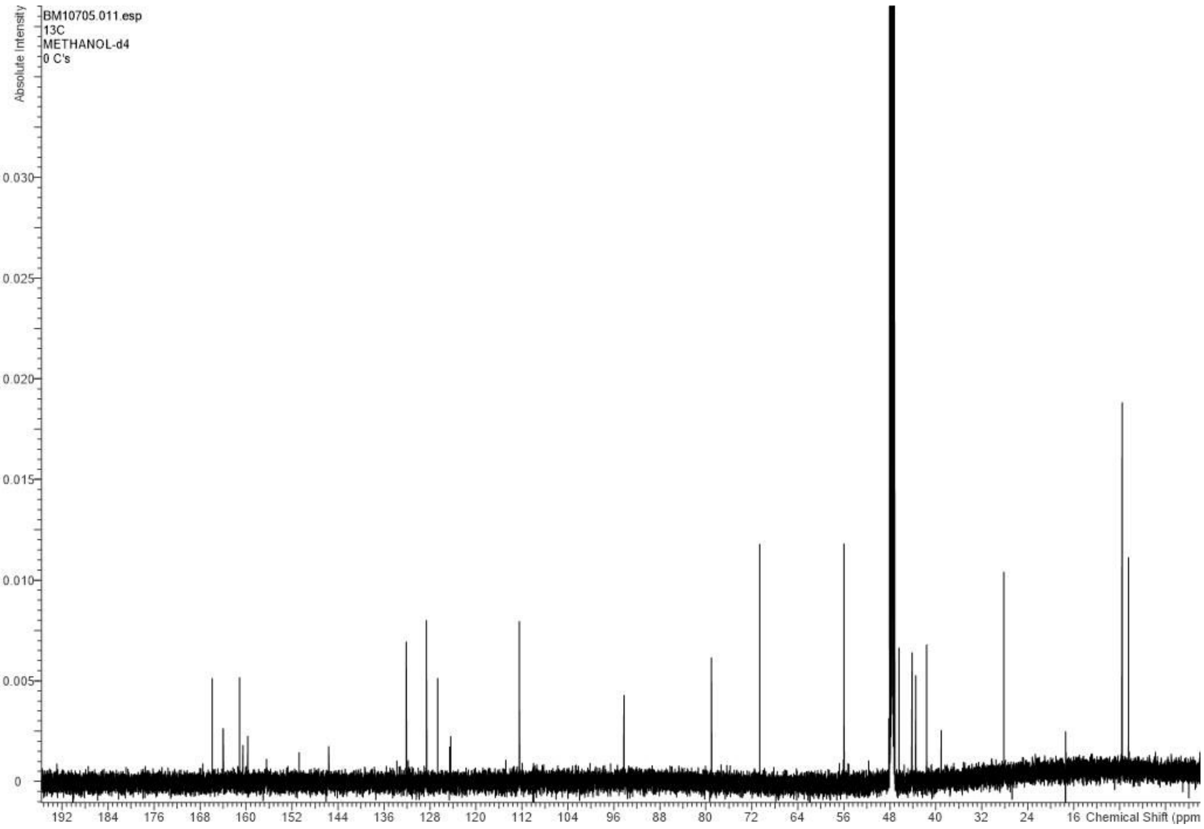

BM10705.011.esp
13C
METHANOL-d4
0 C's
Absolute Intensity
Chemical Shift (ppm

5g

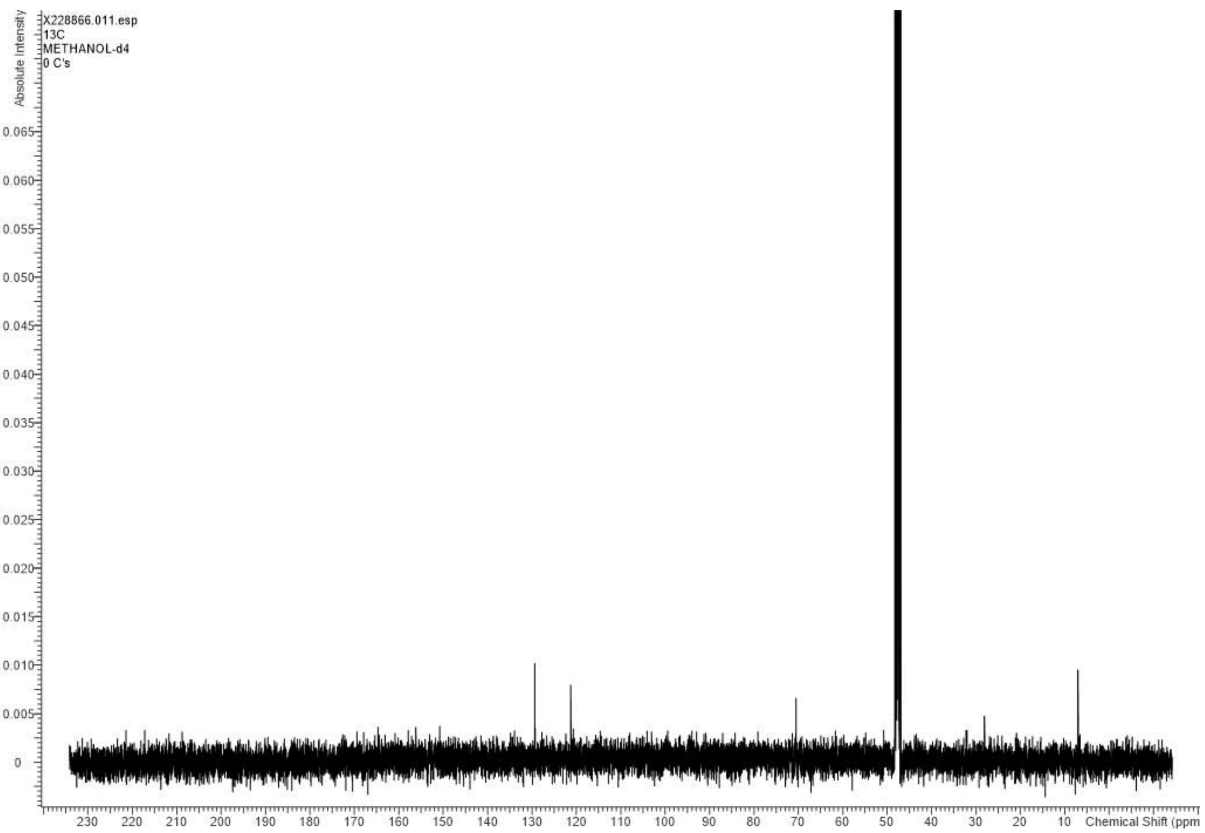
X228866.011.esp
13C
METHANOL-d4
0 C's
Absolute Intensity
0.065
0.060
0.055
0.050
0.045
0.040
0.035
0.030
0.025
0.020
0.015
0.010
0.005
0
230 220 210 200 190 180 170 160 150 140 130 120 110 100 90 80 70 60 50 40 30 20 10
Chemical Shift (ppm

5h

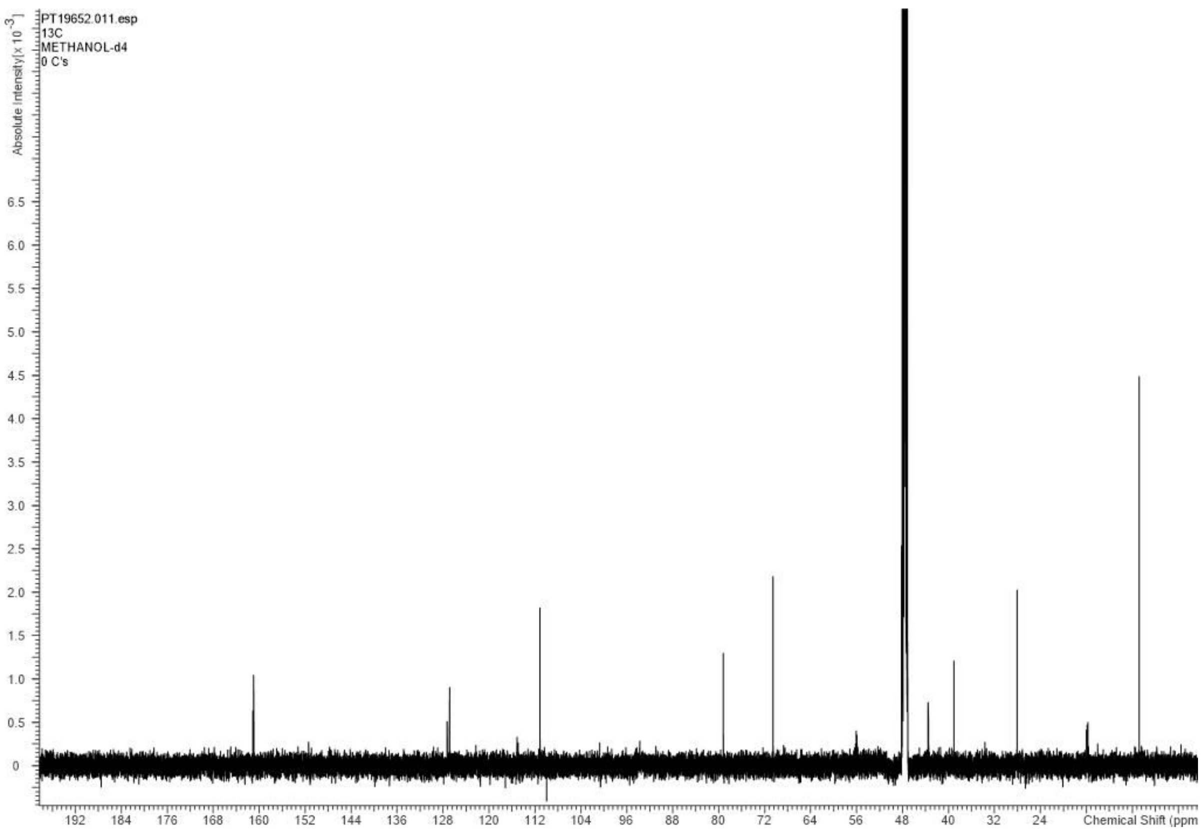
PT19652.011.esp
13C
METHANOL-d4
0 C's
Absolute Intensity(x 10^-3)
6.5
6.0
5.5
5.0
4.5
4.0
3.5
3.0
2.5
2.0
1.5
1.0
0.5
0
192 184 176 168 160 152 144 136 128 120 112 104 96 88 80 72 64 56 48 40 32 24
Chemical Shift (ppm

5i

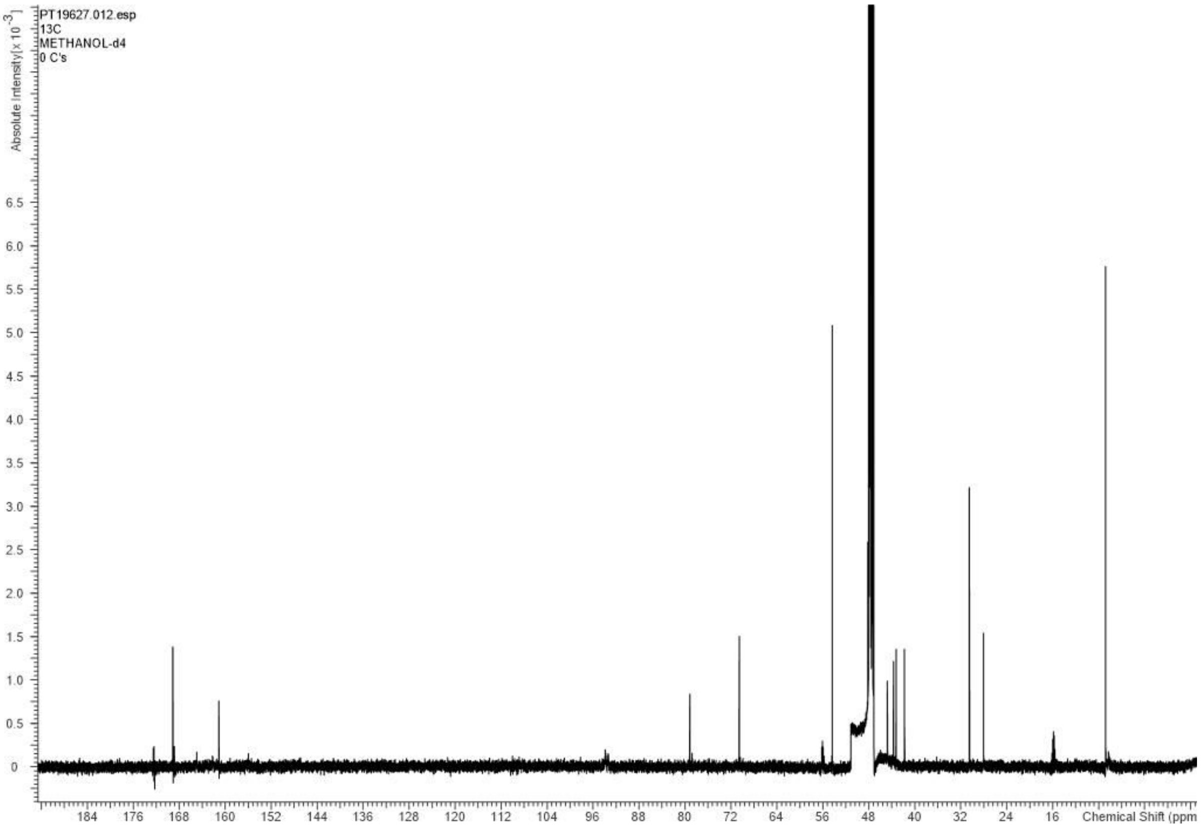
PT19627.012.esp
13C
METHANOL-d4
0 C's
Absolute Intensity[x 10^-3]
6.5
6.0
5.5
5.0
4.5
4.0
3.5
3.0
2.5
2.0
1.5
1.0
0.5
0
184
176
168
160
152
144
136
128
120
112
104
96
88
80
72
64
56
48
40
32
24
16
Chemical Shift (ppm

12. References

(1) Sicilio, F.; Peterson, M. D. Ratio Errors in Pseudo First Order Reactions. *J. Chem. Educ.* **1961**, *38* (11), 576–577. https://doi.org/10.1021/ed038p576.
